# Meditative Movement Therapies and Health-Related Quality-of-Life in Adults: A Systematic Review of Meta-Analyses

**DOI:** 10.1371/journal.pone.0129181

**Published:** 2015-06-08

**Authors:** George A. Kelley, Kristi S. Kelley

**Affiliations:** Department of Biostatistics, West Virginia University, Morgantown, West Virginia, United States of America; Chiba University Graduate School of Medicine, JAPAN

## Abstract

Poor health-related quality-of-life (HRQOL) is a significant public health issue while the use of meditative movement therapies has been increasing. The purpose of this investigation was to carry out a systematic review of previous meta-analyses that examined the effects of meditative movement therapies (yoga, tai chi and qigong) on HRQOL in adults. Previous meta-analyses of randomized controlled trials published up through February, 2014 were included by searching nine electronic databases and cross-referencing. Dual-selection and data abstraction occurred. The Assessment of Multiple Systematic Reviews Instrument (AMSTAR) was used to assess methodological quality. Standardized mean differences that were pooled using random-effects models were included. In addition, 95% prediction intervals were calculated as well as the number needed-to-treat and percentile improvements. Of the 510 citations screened, 10 meta-analyses representing a median of 3 standardized mean differences in 82 to 528 participants (median = 270) with breast cancer, schizophrenia, low back pain, heart failure and diabetes, were included. Median methodological quality was 70%. Median length, frequency and duration of the meditative movement therapies were 12 weeks, 3 times per week, for 71 minutes per session. The majority of results (78.9%) favored statistically significant improvements (non-overlapping 95% confidence intervals) in HRQOL, with standardized mean differences ranging from 0.18 to 2.28. More than half of the results yielded statistically significant heterogeneity (Q ≤ 0.10) and large or very large inconsistency (*I^2^* ≥ 50%). All 95% prediction intervals included zero. The number-needed-to-treat ranged from 2 to 10 while percentile improvements ranged from 9.9 to 48.9. The results of this study suggest that meditative movement therapies may improve HRQOL in adults with selected conditions. However, a need exists for a large, more inclusive meta-analysis (PROSPERO Registration #CRD42014014576).

## Introduction

Poor health-related quality-of-life (HRQOL) is a significant public health issue. For example, in the United States (US), the age-adjusted prevalence of adults 18 years of age and older who rated their health as fair or poor was estimated to be 16.1% [1]. Meditative movement therapies (MMT) such as yoga, tai chi and qigong have become increasingly popular and offer a potential approach for improving HRQOL in adults. In 2007, the age-adjusted prevalence of US adults ages 18 years and older who participated in yoga (12.8 million), tai chi (2.1 million), and qigong (1 million) totaled approximately 15.9 million, an increase of approximately 2 million when compared to 2002 data [2].

Systematic reviews with meta-analysis are considered to be the gold standard for determining the effects of an intervention on an outcome [3,4]. However, a number of previous systematic reviews with meta-analysis now exist on the same topic [5,6]. As a result, it becomes difficult to make confident decisions about the effectiveness of an intervention on a chosen outcome in the population of interest [5,7]. Consequently, it is now necessary to systematically review these previous reviews in order to provide decision-makers and practitioners with the information needed to make evidence-based decisions and recommendations regarding the effects of an intervention on an outcome as well as provide investigators with suggestions for future inquiry [5,7]. To the best of the investigative team’s knowledge, no previous systematic review of systematic reviews with meta-analysis addressing the effects of MMT (yoga, tai chi and qigong) on HRQOL in adults has been conducted. The purpose of this study was to address this gap.

## Methods

### Study Eligibility

This systematic review of previous systematic reviews with meta-analysis is registered in the PROSPERO trial registry (CRD42014014576). Given that no guidelines currently exist for conducting systematic reviews of previous systematic reviews with meta-analysis, the general guidelines of the Preferred Reporting Items for Systematic Reviews and Meta-Analysis (PRISMA) Statement [8], where applicable, was followed. Finally, the methods described below have been previously reported in detail on a different topic addressing the effects of aerobic and strength training exercise on depressive symptoms in adults with arthritis and other rheumatic disease [7].

The *a priori* inclusion criteria for this study were as follows: (1) previous systematic reviews with meta-analysis of randomized controlled intervention trials or data reported separately for randomized controlled trials if the meta-analysis included other study designs, (2) adults 18 years of age and older, (3) yoga, tai chi or qigong as the intervention, (4) published and unpublished (dissertations and master’s theses) studies in any language up through February of 2014, (5) intervention minus control group difference in HRQOL as a primary outcome in the original meta-analysis and reported as the standardized mean difference (SMD) effect size or calculable using the SMD with at least two pooled studies. Meta-analyses were limited to randomized controlled trials because they are the only way to control for unknown confounders as well as the fact that nonrandomized controlled trials tend to overestimate the effects of treatment in healthcare interventions [9,10]. Given the different instruments used to assess HRQOL, the SMD was the metric of choice. Any studies that did not meet all of the above criteria were excluded. Ineligible studies were excluded based on one or more of the following: (1) inappropriate population (for example, children), (2) inappropriate intervention (for example, aerobic exercise), (3) inappropriate comparison (for example, yoga versus drug), (4) inappropriate outcome (for example, depression), (5) inappropriate study type (for example, systematic review without meta-analysis).

### Data Sources

The graphical-user interfaces of the following nine electronic databases were searched from their inception forward for potentially eligible studies: (1) PubMed (1966 to February 26, 2014), (2) Sport Discus (1975 to February 26, 2014), (3) Web of Science (1955 to February 26, 2014), (4) Scopus (1823 to February 27, 2014), (5) PsychInfo (1800’s to February 27, 2014), (6) Cochrane Database of Systematic Reviews (1996 to February 26, 2014), (7) Physiotherapy Evidence Database [(PEDRO) (1929 to March 20, 2014)], (8) Database of Abstract of Reviews of Effects [(DARE) (1991 to February 27, 2014], (9) Proquest (1861 to February 28, 2014). Scopus was included in the database searches because it has been reported to provide coverage of EMBASE, a database that was not available to the investigators [11]. While the specific search strategies varied depending on the database searched, key terms or forms of key terms included yoga, tai chi, qigong, quality of life, randomized, systematic review and meta-analysis. A copy of the search strategies used for each database is shown in S1 File. After removing duplicates, the overall precision of the searches was calculated by dividing the number of studies that met the eligibility criteria by the total number of studies screened [12]. The number needed to read (NNR) was then calculated as the inverse of the precision [12]. In addition to electronic database searches, cross-referencing for potentially eligible meta-analyses from retrieved reviews was also conducted. All studies were stored in Reference Manager, version 12.0 [13].

### Study Selection

All studies were selected by both authors, independent of each other. They then met and reviewed their selections for agreement. Any disagreements were resolved by consensus.

### Data Abstraction

Prior to data abstraction, coding sheets were developed in Microsoft Excel 2010 [14]. The coding sheets could hold up to 180 items from each included meta-analysis. The major categories of variables coded included (1) study characteristics (source, year, etc.), (2) participant characteristics (age, gender, condition, etc.), (3) intervention characteristics (length, frequency, duration, setting, type of MMT, etc.), and (4) results for HRQOL (mean change, precision, z-scores, heterogeneity, inconsistency, publication bias, etc.) Data was abstracted by both authors, independent of each other. Upon completion of coding, all coding sheets were merged into one common codebook and reviewed by both authors for correctness. Disagreements were resolved by consensus. Using Cohen’s kappa statistic [15], the overall agreement rate prior to correcting discrepancies was *k* = 0.95, considered to be “excellent” [16].

### Methodological Quality

The Assessment of Multiple Systematic Reviews (AMSTAR) Instrument was used to assess the methodological quality of each included meta-analysis [17–20]. The AMSTAR instrument was chosen over others [21,22] because of its reported construct validity (intra-class correlation coefficient = 0.84), inter-rater reliability (*k* = 0.70) and feasibility (average of 15 minutes per study to complete) [19]. The 11-item questionnaire is designed to elicit responses of “Yes”, “No”, “Can’t Answer”, or “Not Applicable”. The response “Can’t Answer” is chosen when an item is relevant but not described. The response “Not Applicable” is chosen when an item is not relevant (for example, assessment of publication bias not possible because of the lack of studies) [17–20]. For consistency when summing responses, the following question was modified from “Was the status of publication (i.e. grey literature) used as an inclusion criterion?” to “Was the status of publication (i.e. grey literature) as an inclusion criterion avoided?” Both authors assessed the methodological quality of each study independent of each other. They then met and reviewed every item for agreement. Disagreements were resolved by consensus. The overall agreement rate prior to correcting discrepancies was *k* = 0.80, considered to be “excellent” [16]. The use of a strength of evidence instrument such as the Grading of Recommendations Assessment, Development and Evaluation (GRADE) tool [23] was avoided based on the belief that it may be too conservative, especially for an intervention such as MMT where the chance of adverse events is probably minimal.

To assess the impact of the included meta-analyses, the total number of times that each included meta-analysis was cited as well as the average number of citations per year was estimated. This was accomplished using version 4.4.6 of Publish or Perish (Google Scholar Citation mechanism) [24] on August 24, 2014.

### Data Synthesis

The main results from each meta-analysis were extracted with a focus on random-effects models because they incorporate between-study heterogeneity into the model [25,26]. The SMD, 95% confidence intervals (CIs) and associated z and alpha value for z were abstracted or calculated if sufficient data were available to do so. Standardized mean differences were classified as trivial (<0.20), small (0.20 to 0.49), medium (0.50 to 0.79) or large (≥0.80) [27]. Non-overlapping 95% CIs were considered statistically significant. The Q statistic, a measure of heterogeneity, was also extracted for each outcome. An alpha value ≤ 0.10 was considered to represent statistically significant heterogeneity [28]. Because of issues surrounding the power of the Q statistic, the *I* ^*2*^ statistic, a measure of inconsistency, was also reported if it was provided in the meta-analysis. If *I* ^*2*^ was not reported, it was calculated if sufficient data was available [28]. I-squared values were classified as low (0 to <25%), moderate (25 to <50%), large (50 to <75%) or very large (≥ 75%) inconsistency [28]. An *a priori* decision was made to not pool results from the different meta-analyses because of the expectation that many of the same original studies would be included in the different meta-analyses, thus violating the assumption of independence.

An *a priori* assumption was made that none of the eligible meta-analyses would include 95% prediction intervals (PIs) [29–31]. Therefore, PIs were calculated if the findings were statistically significant and the necessary data from each study included in each meta-analysis were provided [29–31]. Prediction intervals are used to estimate the treatment effect in a new trial [29–31] and may be more appropriate for decision analysis [32].

In order to reinforce practical application, the number-needed-to treat (NNT) was calculated for any overall findings that were reported as statistically significant. This was achieved using the method suggested by the Cochrane Collaboration and was based on a control group risk of 30% [3]. In addition, Cohen’s U_3_ index was used to determine the percentile gain in the intervention group [33]. For example, a SMD of 0.25 suggests that on average, a person in the MMT group would be at approximately the 60^th^ percentile in terms of improving their HRQOL. This translates into being approximately 10 percentiles higher than the control group [34].

If not already provided and if sufficient data and number of effect sizes (N ≥ 10) were available to do so [35], small-study effects (publication bias, etc.) were assessed using the regression-intercept approach of Egger et al. [36]. Non-overlapping, one-tailed 95% CIs were considered to be representative of statistically significant small-study effects. Positive SMD’s were indicative of improvements in HRQOL. Analyses were carried out using Comprehensive Meta-Analysis (version 2.2) [37] and Microsoft Excel 2010 [14].

Finally, post-hoc descriptive analysis according to condition and type of MMT was conducted.

## Results

### Characteristics of Included Meta-Analyses

**Fig 1:**
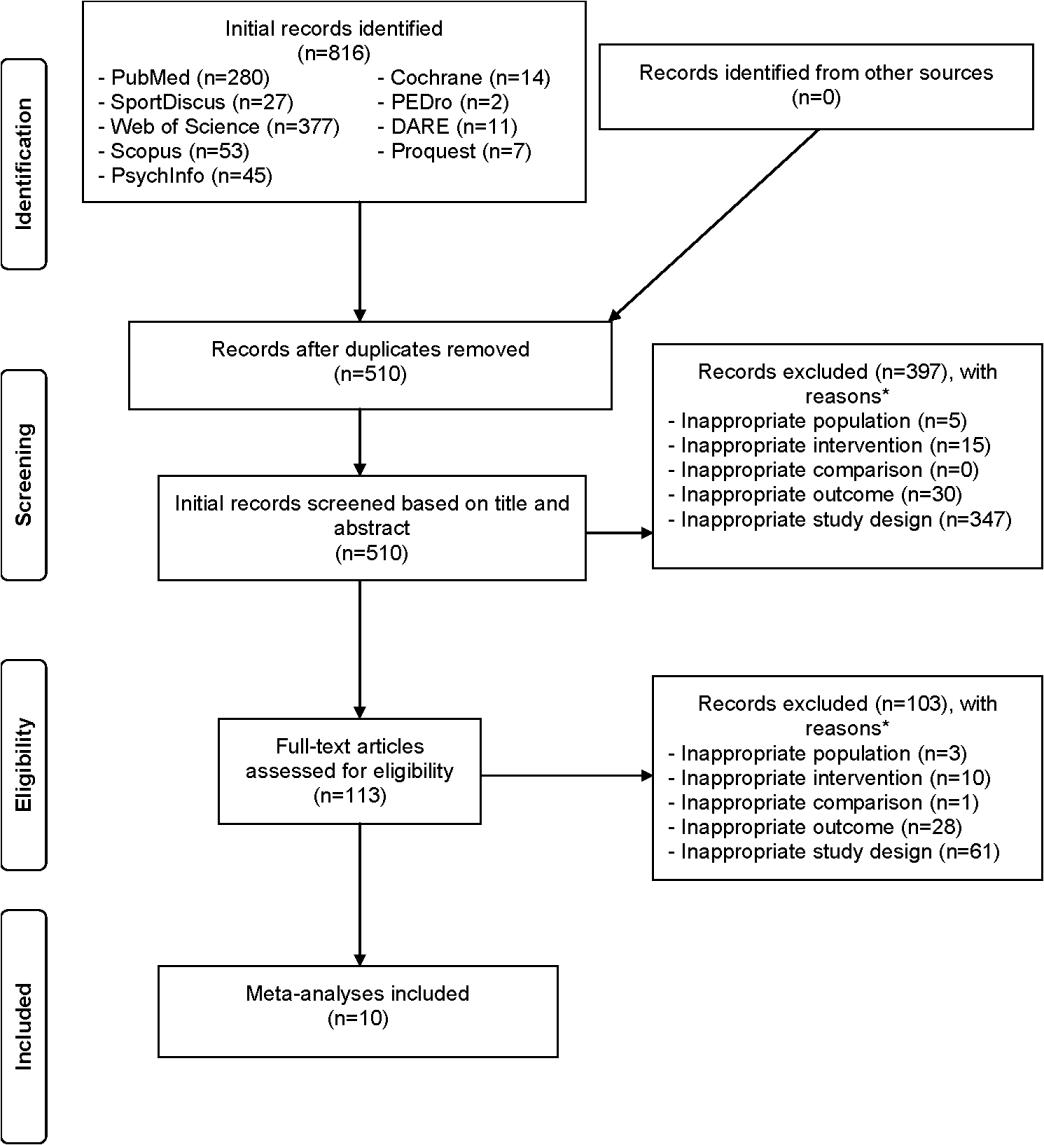
Flow Diagram for the selection of studies. *, number of reasons exceeds the number of studies because some studies were excluded for more than one reason.

A total of 816 references were initially recognized. Post-duplicate removal, 510 (62.5%) remained. Of the 510 screened, 10 meta-analyses, all using the aggregate data approach, met all study eligibility criteria [38–47]. Search precision was 0.02 whilst the NNR was 51. Ineligible studies were excluded based on an inappropriate study design (81.6%) as well as inappropriate outcome (11.6%), intervention (5.0%), population (1.6%), and comparison (0.2%). Two meta-analyses in which results were reported using the original metric were converted to SMDs [43,46]. Fig 1 illustrates the search process while the references for the 500 excluded studies, including the reasons for rejection, can be found in S2 File. As can be seen in Table 1, three studies were conducted in Germany, all by the same investigative team [39–41], three in China [43,46,47], and one each in either the United States [45], United Kingdom [44], Netherlands [38], or Taiwan [42]. Five of the 10 meta-analyses reported receiving funding for their work [38–40,44,45] while nine reported no competing interests [38–41,43–47]. Another study did not provide any information on competing interests [42]. All of the meta-analyses focused on participants with specific conditions [38–47]. With respect to gender, four meta-analyses were limited to women with breast cancer [38,39,42,47] while the remaining six included both men and women [40,41,43–46]. A lack of data was provided for race and ethnicity. For MMT, seven of the 10 meta-analyses were limited to yoga [38–42,44,47], while one each was limited to either tai chi [43], qigong [45], or both [46]. Based on the availability of data, the length of the interventions within each study included in the meta-analyses ranged from 1 to 24 weeks, frequency from 1 to 9 times per week and duration from 30 to 120 minutes per session. The mean length of the interventions for each meta-analysis ranged from 8 to 16 weeks ($\bar{\text{X}}$ ± SD, 11.9 ± 2.6, Median = 12), frequency from 2 to 6 times per week ($\bar{\text{X}}$ ± SD, 3.2 ± 1.5, Median = 3), and duration from 53 to 80 minutes per session ($\bar{\text{X}}$ ± SD, 68.9 ± 10.3, Median = 71). Supervised as well unsupervised MMT sessions occurred at a facility and/or home. Data on compliance to the MMT sessions were lacking. For the four meta-analyses that provided information, no serious adverse events were identified from the studies that included the assessment of HRQOL [38–41]. A total of 12 different instruments were used to assess HRQOL and included both generic and disease-specific questionnaires [38–41].

**Table 1:** General characteristics of included meta-analyses.

Reference	Year	Country	Studies	Participants	Interventions	HRQOL Assessment
Buffart et al.[38]	2012	Netherlands	7	528 women with breast cancer, 28–75 years of age ($\bar{X}\pm$ SD, 52.0 ± 4.5)*	Supervised/unsupervised yoga interventions lasting 6–24 weeks ($\bar{X}\pm$ SD, 10 ± 7), frequency of 3-9x week ($\bar{X}\pm$ SD, 6 ± 2), duration of 60–90 minutes per session ($\bar{X}\pm$ SD, 68 ± 13)	SF-36, EORTC QLQ-C30, FACT G, FLIC
Cramer et al.[39]	2012	Germany	4	274 women with breast cancer (155 yoga, 119 control), age, $\bar{X}\pm$ SD, 57.1 ± 3.1)	Yoga interventions lasting 10–24 weeks ($\bar{X}\pm$ SD, 15 + 8), frequency of 1 to 4x week ($\bar{X}\pm$ SD, 2 ± 2), duration of 30–90 minutes per session,($\bar{X}\pm$ SD, 71 ± 28)	SF-12, FACT B, FACT G, FLIC, FACT-Sp
Cramer et al.[40]	2013	Germany	2	98 men and women (48 yoga, 50 control), all with schizophrenia, age ($\bar{X}\pm$ SD, 36.1 ± 9.5 years	Yoga interventions lasting 8 weeks, frequency of 2-3x week ($\bar{X}\pm$ SD, 3 ± 2), duration of 45 to 60 minutes per session, ($\bar{X}\pm$ SD, 53 ± 11)	GQOLI-74, WHO-QOL-BREF
Cramer et al.[41]	2013	Germany	4	388 men and women with low back pain (187 yoga, 201 control), 44 to 49 years of age ($\bar{X}\pm$ SD, 46.0 ± 1.9) years)	Supervised and unsupervised yoga interventions lasting 1–12 weeks ($\bar{X}\pm$ SD, 9 ± 6) frequency ≤ 7x week, duration of 30 to 75 minutes per session	SF-12, SF-36, EQ5D, WHO-QOL-BREF
Lin et al.[42]	2011	Taiwan	3	191 women with breast cancer (115 yoga, 76 control), 51 to 56 years of age ($\bar{X}\pm$ SD, 54.0 ± 2.0 years)	Supervised and unsupervised yoga interventions lasting 7–12 weeks ($\bar{X}\pm$ SD, 10 ± 3), duration of 75–90 minutes per session,($\bar{X}\pm$ SD, 80 ± 9)	SF-12, FACT B, FACT G, EORTC QLQ-C30
Pan et al.[43]	2013	China	3	182 men and women with heart failure (90 tai chi, 92 control), 64 to 70 years of age ($\bar{X}\pm$ SD, 66.8 ± 3.0 years)	Tai chi interventions lasting 12–16 weeks ($\bar{X}\pm$ SD, 13 ± 2), frequency of 2x week, duration of 55 to 60 minutes per session, ($\bar{X}\pm$ SD, 58 ± 3)	MLHF
Shneerson et al.[44]	2013	United Kingdom	3	153 men and women with cancer, primarily breast cancer (87 yoga, 66 control), 50 to 63 years of age ($\bar{X}\pm$ SD, 57.0 ± 5.0 years)	Supervised yoga interventions lasting 7–24 weeks ($\bar{X}\pm$ SD, 12 ± 7), at least one session per week, duration of 60–90 minutes per session	FACT B, FACT G, EORTC QLQ-C30
Wang et al.[45]	2013	United States	2	172 men and women with diabetes, (120 qi gong, 52 control), 37 to 69 years of age ($\bar{X}\pm$ SD, 57.9 ± 0.1 years)	qigong interventions lasting 16 weeks	DSQL
Zeng et al.[46]	2014	China	5	405 men and women with cancer, (200 tai chi or qigong, 205 control), ≥ 18 years of age	Supervised and unsupervised tai chi and qigong interventions lasting 6–24 weeks ($\bar{X}\pm$ SD, 12 + 7), frequency of 2-7x week ($\bar{X}\pm$ SD, 4 + 2), duration of 40–120 minutes per session ($\bar{X}\pm$ SD, 78 ± 46)	SF-36^a^, FACT G
Zhang et al.[47]	2012	China	4	270 women with breast cancer, (154 yoga, 116 control), 53 to 59 years of age ($\bar{X}\pm$ SD, 55.6 ± 3.0 years)	Yoga interventions lasting 6–24 weeks ($\bar{X}\pm$ SD, 13 ± 8), frequency of 1-5x week ($\bar{X}\pm$ SD, 2 ± 2), duration of 60–90 minutes per session ($\bar{X}\pm$ SD, 75 ± 12)	FACT B, FACT G

Notes: $\bar{X}\pm$ SD, mean + standard deviation; Description of meta-analyses limited to those studies nested within each meta-analysis that met all eligibility criteria for the current study; Data presented limited to what was reported or could be calculated from reported data; Number of participants limited to those in which a SMD was calculated; SF-36, Medical Outcomes Short-form Health Survey-36; EORTC QLQ-C30, European Organization for the Research and Treatment of Cancer-Quality of Life; FACT G, Functional Assessment of Cancer Therapy-General; FLIC, Functional Living Index for Cancer; SF-12, Medical Outcomes Short-form Health Survey-12; FACT B, Functional Assessment of Cancer Therapy-Breast; FACT-Sp, Functional Assessment of Cancer Therapy–Spirituality; GQOLI-74, General Quality of Life Inventory; WHO-QOL-BREF, WHO Quality of Life-BREF quality of life assessment; EQ5D, EuroQol 5 Digit Questionnaire; MLHF, Minnesota Living With Heart Failure Questionnaire; DSQL, Diabetes Specific Quality-of-Life Scale; SF-36^a^ results also reported but excluded because results were for 8 subdomains versus physical and mental component scores.

*, separate sample sizes not available for yoga and control groups.

### Methodological Quality and Impact

S1 Table shows the results for each meta-analysis using the AMSTAR instrument. Across all categories, scores ranged from 50% to 70% ($\bar{\text{X}}$ ± SD, 66.3% ± 6.6%, Median = 70%). All included meta-analyses were considered to have satisfied five of the 11 criteria: (1) “a priori” design, (2) characteristics of studies table, (3) quality/risk of bias assessment, (4) inclusion of quality/risk of bias assessment in formulating conclusions, and (5) methods used for pooling results [38–47]. In contrast, none of the included meta-analyses adequately addressed the question regarding conflict of interest, all because they did not report information on potential sources of support from each of the studies included in their meta-analysis [38–47]. Similarly, none of the meta-analyses adequately addressed the two questions about including all eligible studies regardless of publication status as well as providing a reference list of eligible and ineligible studies, the latter because none provide a list of ineligible studies, including the reasons for exclusion [38–47]. Finally, because of the small number of studies included in each meta-analysis and based on current guidelines that at least 10 effect sizes be available to conduct tests for small-study effects (publication bas, etc.) [35], no “Yes” responses were recorded.

With respect to impact, the total number of times that each meta-analysis was cited ranged from 1 to 63 ($\bar{\text{X}}$ ± SD, 20 ± 20, Median = 12). When adjusted for the number of years that each meta-analysis was available, the total number of times that each meta-analysis was cited ranged from 1 to 43 ($\bar{\text{X}}$ ± SD, 12 ± 13, Median = 8).

### Data Synthesis

**Fig 2:**
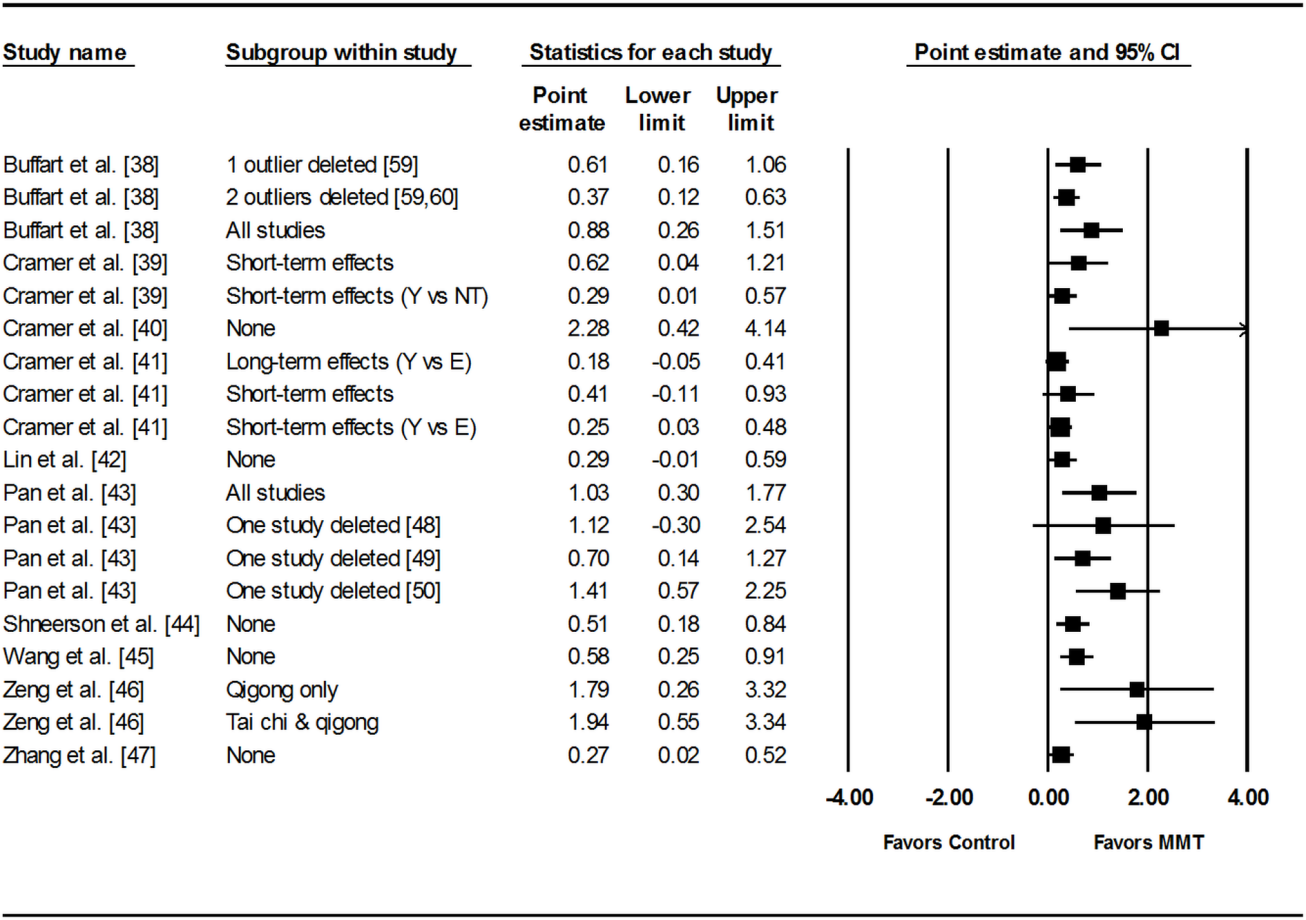
Forest plot for standardized mean difference effect size changes in HRQOL. The black squares represent the pooled standardized mean difference effect size for each analysis while the left and right extremes of the squares represent the corresponding 95% confidence intervals for the pooled standardized mean difference effect size for each analysis. All analyses are based on a random-effects model and not pooled across all analyses because some of the results included the same studies. The numbers in brackets represent reference numbers. Y, Yoga; NT, No Treatment; E, Education; MMT, Meditative Movement Therapies.

Table 2 and Fig 2 show the overall results for the 10 included systematic reviews with meta-analysis [38–47]. The number of SMDs for each HRQOL analysis ranged from 2 to 7 ($\bar{\text{X}}$ ± SD, 3 ± 1, Median = 3) while the number of participants nested within each analysis ranged from 82 to 528 ($\bar{\text{X}}$ ± SD, 269 ± 131, Median = 270). Across all analyses, statistically significant improvements in HRQOL were observed for 15 of the 19 (78.9%) results. Changes in HRQOL ranged from a low of 0.18 (trivial effect) to a high of 2.28 (large effect). For the 15 results that were statistically significant, 10 (66.7%) yielded statistically significant results for heterogeneity based on the Q statistic. In contrast, only 6 of the 15 statistically significant results (46.7%) were considered to display very large inconsistency as indicated by the *I* ^*2*^ statistic. For the nine PI that could be calculated, all included zero (0), i.e., not statistically significant. Publication bias was not examined or calculated because all of the analyses included a sample size of less than 10.

**Table 2:** Overall post-treatment standardized mean difference (SMD) effect sizes for HRQOL from included meta-analyses.

Reference	ES/Participants						
	(No.)	SMD (95% CI)	Z (p)	Q (p)	*I* ^*2*(%)^	T^2^	PI (95%)
Buffart et al.[38]							
-All studies	7/528	**0.88 (0.25, 1.50)** *	2.75 (0.006)	**44.4 (<0.001)** **	87		
- One outlier deleted[59]	6/467	**0.61 (0.16, 1.06)** *	2.50 (0.008)	**16.55 (0.005)** **	70		
- Two outliers deleted[59,60]	5/405	**0.37 (0.11, 0.62)** *	2.85 (0.004)	3.40(0.49)	0	0.00	-0.04, 0.78
Cramer et al.[39]							
-Short-term effects^c^	4/274	**0.62 (0.04, 1.21)** *	2.08 (0.04)	**14.48 (0.002)** **	79	0.28	-1.99, 3.23
-Short-term effects (Y vs NT)	3/212	**0.29 (0.01, 0.57)** *	2.08 (0.04)	0.75 (0.69)	0	0.00	-1.53, 2.11
Cramer et al.[40]	2/98	**2.28 (0.42, 4.14)** *	2.40 (0.02)	**9.01 (0.003)** **	89	1.62	—
Cramer et al.[41]							
-Short-term effects^c^	4/388	0.41 (-0.10, 0.93)	1.54 (0.12)	**10.7 (0.01)** **	72	0.19	NA
-Short-term effects (Y vs E)^c^	3/308	**0.25 (0.02, 0.47)** *	2.17 (0.03)	1.25 (0.54)	0	0.00	-1.21, 1.71
-Long-term effects (Y vs E)^d^	2/287	0.18 (-0.05, 0.41)	1.52 (0.13)	0.10 (0.76)	0	0.00	NA
Lin et al.[42]	3/191	0.29 (-0.01, 0.58)^**a**^	1.91 (0.06)	1.34 (0.51)	0	0.00	NA
Pan et al.[43]							
-All studies	3/190	**1.03 (0.29, 1.76)** * ^**,**^ ^**a**^ ^**,**^ ^**b**^	2.75 (0.01)	**9.64 (0.01)** **	79	0.33	-7.69, 9.75
-One study deleted[48]	2/130	1.12 (-0.29, 2.54) ^**a**^ ^**,**^ ^**b**^	1.55 (0.12)	**8.95 (<0.001)** **	88	0.93	NA
-One study deleted[49]	2/152	**0.70 (0.14, 1.27)** * ^**,**^ ^**a**^ ^**,**^ ^**b**^	2.43 (0.02)	**2.66 (0.10)** **	62	0.11	—
-One study deleted[50]	2/82	1.41 (0.57, 2.25)* ^**,**^ ^**a**^ ^**,**^ ^**b**^	3.29 (0.001)	**2.66 (0.10)** **	62	0.23	—
Shneerson et al.[44]	3/153	**0.51 (0.18, 0.84)** *	3.06 (0.002)	0.33 (0.85)	0	0.00	-1.63, 2.65
Wang et al.[45]	2/172	**0.58 (0.25, 0.91)** * ^**,**^ ^**a**^	3.40 (0.0007)	0.16 (0.69)	0	0.00	—
Zeng et al.[46]							
-Tai chi & qigong	5/405	**1.94 (0.59, 3.38)** * ^**,**^ ^**a**^ ^**,**^ ^**b**^	2.80 (0.005)	**116.9 (<0.001)** **	97	2.33	-3.42, 7.30
-Qigong only	4/395	**1.79 (0.26, 3.32)** * ^**,**^ ^**a**^ ^**,**^ ^**b**^	2.29 (0.02)	**114.4 (<0.001)** **	97	2.38	-5.65, 9.23
Zhang et al.[47]	4/270	**0.27 (0.02, 0.52)** *	2.15 (0.03)	0.88 (0.83)	0	0.00	-0.28, 0.82

Notes: No., Number; ES, effect size; SMD, standardized mean difference effect size; 95% CI, 95% confidence intervals; Z(p), Z-value and probability value for Z; Q(p), Cochran’s Q statistic and associated alpha (p) value for Q; *I* ^*2*^, I-squared statistic for inconsistency; T^2^, tau-squared; PI, prediction intervals, based on a random-effects model; Y vs NT, yoga versus no treatment; Y vs E, yoga versus education;—, Data not provided or insufficient data to calculate; SMD (95% CI) based on random-effects model; NA, not applicable; **Boldfaced**

*, statistically significant non-overlapping confidence intervals

**, statistically significant at an alpha level ≤ 0.10

^a^, Data reverse-scaled to be consistent with other studies in which a positive SMD was indicative of improvements in HRQOL

^b^, Data converted from original metric to standardized mean difference effect size for comparison purposes

^c^, short-term effects, HRQOL assessed closest to the end of the intervention

^d^, long-term effects, HRQOL assessed closest to 12 months after randomization

When examined according to condition, the six studies that focused on cancer participants, primarily breast cancer [38,39,42,44,46,47], yielded nine of 10 (90.0%) statistically significant findings for HRQOL. Across all 10 results, SMD effect sizes ranged from 0.27 (small effect) to 1.94 (large effect). Statistically significant heterogeneity (Q) was observed for five of the 10 (50%) findings while very large inconsistency was observed for four (40%). For schizophrenia, a large and statistically significant SMD along with statistically significant heterogeneity and very large inconsistency was observed for HRQOL [40]. For low back pain, a non-significant SMD was observed for short-term effects, regardless of comparison group, as well as long-term effects when the comparison group was limited to education [41]. In contrast, a small and statistically significant effect was observed for HRQOL along with no statistically significant heterogeneity and inconsistency when results were limited to short-term effects and an education comparison group. For heart failure patients, a large, statistically significant SMD effect size as well as statistically significant heterogeneity and very large amount of inconsistency was observed for HRQOL across all studies [43]. Results were no longer statistically significant when one study was deleted [48] while improvements in HRQOL remained statistically significant along with statistically significant heterogeneity and large inconsistency when either of the other two studies were deleted [49,50]. Finally, for the one study focused on participants with diabetes, a moderate and statistically significant improvement in HRQOL was observed along with no statistically significant heterogeneity or inconsistency [45].

When examined according to type of intervention, 10 of 12 results (83.3%) from seven meta-analyses yielded statistically significant SMD improvements in HRQOL when yoga was used as the intervention [38–42,44,47]. Across all 12 results, SMDs for HRQOL ranged from a low of 0.18 (trivial effect) to a high of 2.28 (large effect). For those results that were statistically significant, five of 10 (50%) yielded statistically significant heterogeneity while three (30%) yielded very large inconsistency. The overall results for the meta-analysis that was limited to tai chi yielded a large and statistically significant improvement in HRQOL along with statistically significant heterogeneity and very large inconsistency [43]. However, when each of the three included studies was deleted from the meta-analysis once [48–50], changes ranged from a non-significant SMD of 1.21 to a statistically significant improvement in HRQOL of 1.41 (large effect) that was accompanied by statistically significant heterogeneity and large inconsistency. For the meta-analysis that included qigong as the only intervention and was limited to two studies [45], a statistically significant SMD improvement of 0.58 (moderate effect) was reported for HRQOL along with no statistically significant heterogeneity or inconsistency. Finally, the meta-analysis that included either tai chi or qigong resulted in a large, statistically significant improvement of 1.94 for HRQOL as well as statistically significant heterogeneity and very large inconsistency [46]. When limited to qigong studies only, similar results were obtained.

Number needed-to-treat estimates for those results that were statistically significant can be found in Table 3. As can be seen, the NNT for improving HRQOL ranged from 2 to 10 across all eligible meta-analyses [38–41,43–47]. When limited to selected conditions, the six studies that focused on cancer participants, primarily breast cancer patients [38,39,42,44,46,47], yielded NNT estimates ranging from 2 to 10. For the remaining meta-analyses, NNT values were 2 (schizophrenia) [40], 10 (low back pain) [41], 2 to 3 (heart failure) [43], and 4 (diabetes) [45]. When partitioned according to type of intervention, the NNT ranged from 2 to 10 for yoga [38–41,44,47], 2 to 3 for tai chi only [43], 2 and 4 for qigong only [45,46], and 2 for tai chi and qigong combined [46].

**Fig 3:**
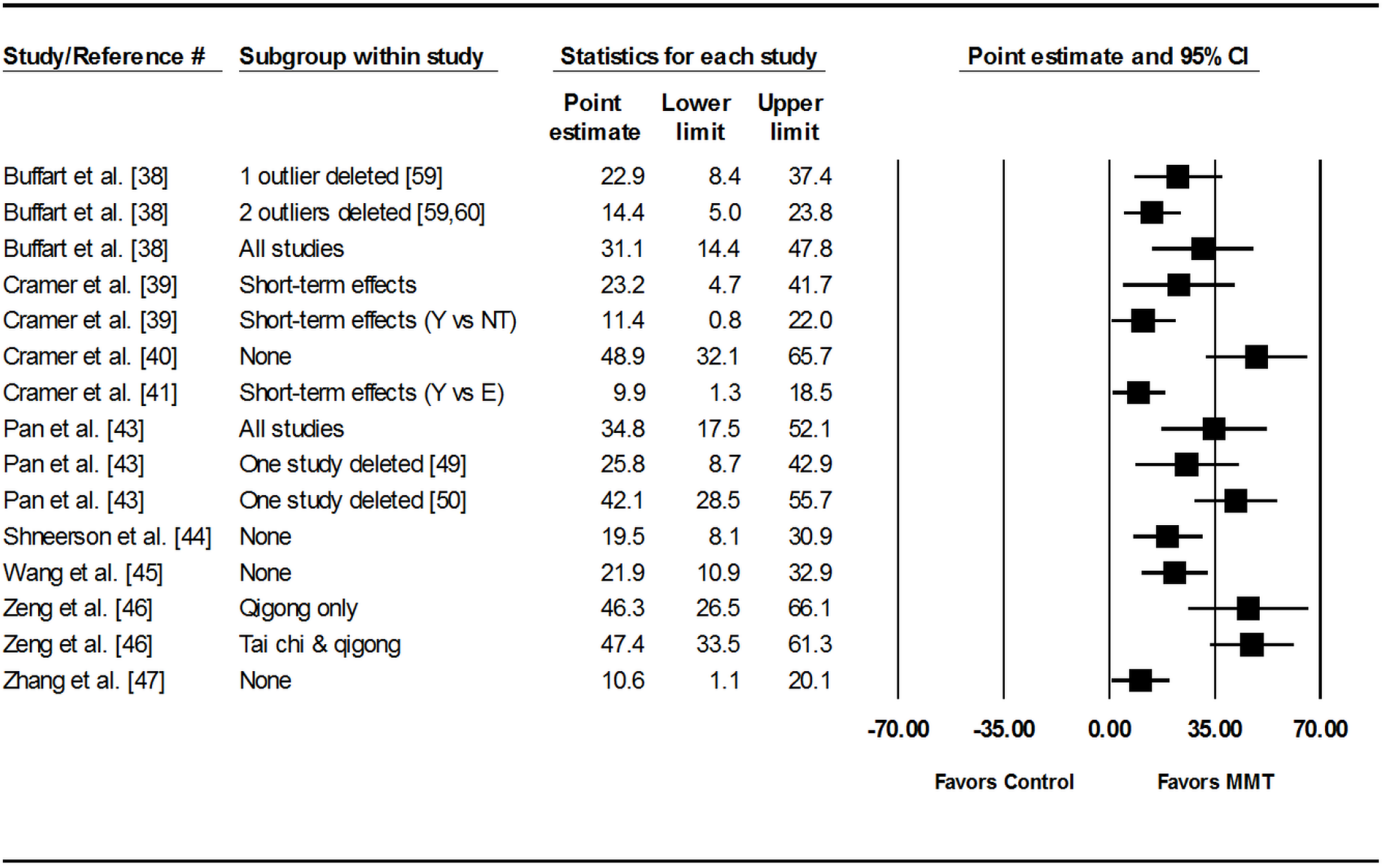
Forest plot for percentile changes in HRQOL. The black squares represent the pooled percentile improvement for each analysis while the left and right extremes of the squares represent the corresponding 95% confidence intervals for percentile improvement for each analysis. All analyses are based on a random-effects model and not pooled across all analyses because some of the results included the same studies. Confidence intervals for each result are not perfectly symmetric because they were calculated separately from the standardized mean difference effect size and corresponding 95%confidence intervals. The numbers in brackets represent reference numbers. Y, Yoga; NT, No Treatment; E, Education; MMT, Meditative Movement Therapies.

Percentile improvements for statistically significant meta-analytic results are also shown in Table 3 and Fig 3. Across all eligible meta-analyses [38–41,43–47], percentile improvements in HRQOL ranged from 9.9 to 48.9. When limited to selected conditions, percentile improvements for the six studies that focused on cancer participants, primarily breast cancer patients [38,39,42,44,46,47], ranged from 10.6 to 46.3. For the remaining meta-analyses, percentile improvements were 48.9 for schizophrenia [40], 9.9 for low back pain [41], 25.8 to 42.1 for heart failure [43] and 21.9 for diabetes [45]. When examined according to type of intervention, percentile improvements ranged from 9.9 to 48.9 for yoga [38–41,44,47], 25.8 to 42.1 for tai chi only [43], 21.9 and 46.3 for qigong only [45,46], and 47.4 for tai chi and qigong combined [46].

**Table 3:** NNT and percentile improvement in HRQOL.

Reference	NNT (95% CI)	U_3_ Index (95% CI)^a^
		(Percentile Improvement)
Buffart et al.[38]		
- All studies	3 (2, 10)	31.1 (9.9, 43.3)
- One outlier deleted[59]	4 (2, 16)	22.9 (6.4, 35.5)
- Two outliers deleted[59,60]	6 (4, 23)	14.4 (4.4, 23.2)
Cramer et al.[39]		
- Short-term effects^b^	4 (2, 65)	23.2 (1.6, 38.7)
- Short-term effects (Y vs NT)^b^	8 (4, 262)	11.4 (0.4, 21.6)
Cramer et al.[40]	2 (1, 6)	48.9 (16.3, 50)
Cramer et al.[41]		
- Short-term effects	NA	NA
- Short-term effects (Y vs E)^b^	10 (5, 131)	9.9 (0.8, 18.1)
- Long-term effects (Y vs E)^c^	NA	NA
Lin et al.[42]	NA	NA
Pan et al.[43]		
- All studies	2 (2, 8)	34.8 (11.4, 46.1)
- One study deleted[48]	NA	NA
- One study deleted[49]	3 (2, 18)	25.8 (5.6, 39.8)
- One study deleted[50]	2 (2, 4)	42.1 (21.6, 48.8)
Shneerson et al.[44]	5 (3, 14)	19.5 (7.1, 30.0)
Wang et al.[45]	4 (3, 10)	21.9 (9.9, 31.9)
Zeng et al.[46]		
-Tai chi & qigong	2 (1, 4)	47.4 (22.2, 50.0)
- Qigong only	2 (1, 9)	46.3 (10.3, 50)
Zhang et al.[47]	9 (4, 131)	10.6 (0.8, 19.8)

Notes: NNT, number needed to-treat, calculated from SMD and 95% confidence intervals for SMD; 95% CI, 95% confidence intervals; Y vs NT, yoga versus no treatment; Y vs E, yoga versus education; NA, not applicable (overlapping 95% confidence intervals for pooled SMD).

^a^, Cohen’s U_3_ Index[33]

^b^, short-term effects, HRQOL assessed closest to the end of the intervention

^c^, long-term effects, HRQOL assessed closest to 12 months after randomization

## Discussion

### Findings

The overall findings of the current study suggest that MMT may have the potential to improve HRQOL in adults with selected conditions. This observation is reinforced by (1) the non-overlapping confidence intervals for the majority (78.9%) of results, (2) low NNT (2 to 10), (3) percentile improvements as a result of MMT (9.9 to 48.9), and (3) good overall quality (median AMSTAR rating = 70%). In contrast, the potentially positive effects of MMT on HRQOL in adults with selected conditions may be weakened by (1) statistically significant heterogeneity for a majority (66.7%) of the positive findings, (2) large to very large inconsistency for more than half (52.6%) of the meta-analyses and (3) overlapping prediction intervals for all of the statistically significant findings.

While the majority of results yielded statistically significant improvements in HRQOL, the magnitude of change varied by 92% across the included conditions (breast cancer, schizophrenia, low back pain, heart failure, diabetes) and interventions (yoga, tai chi, qi gong) [38–47]. Consequently, the NNT and percentile changes also varied widely since they were based on the SMD change in HRQOL. However, whether these wide-ranging changes are the result of the condition, intervention, or some other factor(s), or combination of factors, is not known.

The overall results of the included studies are similar to the effects of traditional types of exercise (aerobic, strength training, etc.) in adults with similar conditions. For example, a meta-analysis of 12 randomized controlled trials of physical exercise (aerobic, strength training, etc.) which did not include any MMT, found a statistically significant SMD improvement of 0.30 (95% CI, 0.12 to 0.48) in HRQOL among breast cancer patients and survivors [51]. This compares to changes in HRQOL ranging from 0.27 to 1.94 for the six breast cancer MMT meta-analyses included in the current study [38,39,42,44,46,47]. For those with schizophrenia or symptoms of schizophrenia, a previous systematic review that was limited to one study found greater increases in HRQOL (SMD = 0.59) when yoga was compared directly to exercise (walking, jogging, etc,) [52]. In comparison, an intervention minus control SMD improvement of 2.28 was found for schizophrenic patients as a result of yoga in the current investigation [40]. Another previous meta-analysis of nine randomized controlled trials in 779 patients with heart failure reported a statistically significant SMD improvement of 0.63 in HRQOL as a result of aerobic and strength training exercise

[53]. For the current systematic review of previous meta-analyses, a SMD improvement of 1.03 was reported for HRQOL for the one meta-analysis that included heart failure patients [43]. Finally, while the investigative team is not aware of any previous meta-analytic research that has examined the effects of exercise on HRQOL in adults with low back pain, a meta-analysis among participants with type 2 diabetes found no statistically significant difference in HRQOL as a result of aerobic exercise for the one trial that was included in their systematic review [54]. In contrast, the one meta-analysis included in the current study found a statistically significant SMD improvement of 0.58 in HRQOL as a result of qigong in participants with diabetes [45].

Based on the previous information, it appears that improvements in HRQOL as a result of MMT are equal to or greater than traditional exercise interventions among adults with breast cancer, schizophrenia, heart failure and diabetes. However, the effects of traditional exercise interventions on HRQOL in adults with low back pain cannot be elucidated given the apparent absence of any previous meta-analytic work in this area. However, when examined irrespective of condition, the results for SMD changes in HRQOL as a result of MMT from the current study (yoga = 0.18 to 2.28, tai chi = 0.70 to 1.41, qigong = 1.79) were larger than those found for a previous meta-analysis of physical activity interventions (0.11) [55].

### Implications for Research

Based on the current study, conducted according to PRISMA guidelines relevant to systematic reviews of previous systematic reviews with meta-analysis (S2 Table), there are at least six inferences for future research using the meta-analytic approach. First, while the median quality of the 10 included meta-analyses was believed to be good [38–47], areas of improvement for future meta-analytic research were noted. These include (1) avoidance of publication status as a criterion for eligibility or providing a strong rationale for not doing so, (2) in addition to providing a reference list of included studies, providing a reference list of excluded studies along with reasons for exclusion, and (3) providing a description of potential conflicts of interest, including potential sources of support, for the studies included in each meta-analysis.

Second, the impact of the included meta-analyses based on citation rates appears to be small. One potential reason for this may be that this work is published in journals that do not have a large readership. A second potential reason may be the lack of universal acceptance of MMT over traditional types of activity such as aerobic exercise and strength training. However, the increasing use of MMT in the United States in recent years is promising [2].

Third, all 10 of the included studies were aggregate data meta-analyses [38–47]. Since an individual-participant data meta-analysis (IPD) has been suggested to be superior to an aggregate data meta-analysis [56], the conduct of an IPD meta-analysis for determining the effects of MMT on HRQOL in adults may be warranted. However, such a decision needs to be made while considering factors such as (1) the ability to retrieve IPD from original study investigators, (2) the increased costs associated with conducting an IPD meta-analysis, and (3) any actual benefit that may be attained by conducting an IPD versus aggregate data meta-analysis [57].

Fourth, since it’s important in meta-analysis to provide practical information for decision-makers (practitioners, policy-makers, etc.), it is suggested that future meta-analytic research on MMT and HRQOL include information such as NNT and/or percentile improvements. While it may also be important to assess the evidence using an instrument such as the Grading of Recommendations Assessment, Development and Evaluation (GRADE) tool [23], this instrument may be too conservative despite its flexibility, especially for an intervention such as MMT where the chance of adverse events is probably minimal. Thus, the use of an instrument such as GRADE may result in a potentially beneficial treatment being inappropriately withheld.

Fifth, it is suggested that PIs be included to enhance the interpretation of findings with respect to the effects of MMT on HRQOL in adults. The use of such cannot only help to determine outcome effects in a new study but also may be more valid for decision-making [31]. However, it’s important to realize that as opposed to CIs, PIs are based on random mean effects [31].

Sixth, all of the meta-analyses included were limited to a small number of randomized controlled trials in participants with certain conditions (breast cancer, schizophrenia, low back pain, heart failure, diabetes), and with the exception of one study [58], a certain type of MMT (yoga, tai chi or qigong). From the investigative team’s perspective, a more powerful and applicable study design would be to conduct a larger meta-analysis of randomized controlled trials that is not limited to either the type of MMT or condition and which also includes healthy adults. One can then perform moderator and/or sensitivity analyses to examine for potential differences in HRQOL according to selected conditions and type of MMT.

Based on the results of the current study, two major recommendations for future randomized controlled trials are suggested. First, given that none of the meta-analyses reported data on the cost-effectiveness of the interventions from the included studies [38–47], it is assumed that the original studies included in each of the meta-analyses did not provide this information. Assuming the former, future intervention studies addressing the effects of MMT on HRQOL should provide this data. Inclusion of this information is critically important to decision-makers when trying to decide which interventions should be prioritized over others.

Second, the dose-response effects of MMT on HRQOL in adults is not known, including what MMT, if any, may be more beneficial for improving HRQOL in adults. Related to this issue is a need to identify what specific types of yoga, tai chi and qigong may be most beneficial given that there are several different types of MMT nested within each of these three modalities. Knowledge of these factors should lead to better treatment in the population of interest.

### Implications for Practice

The results of the current investigation provide important information for practice. First, despite the lack of cost-effectiveness and adverse event data as well as considerable between-study heterogeneity and/or inconsistency for more than half of the reported results, MMT appear to improve HRQOL in the populations studied. While no definitive recommendations can be made and additional research is needed, it would appear both prudent and safe at this time to suggest that HRQOL may be improved by participating in MMT at least 3 times per week for about 71 minutes per session. However, it is important to note that these are general recommendations.

### Strengths and Potential Limitations of Current Study

At least two strengths of the current study were noted. First, the investigative team believes that this is the first systematic review of previous systematic reviews with meta-analysis aimed at determining the effects of MMT on HRQOL in adults, a recent and increasingly necessary method for not only determining the effects of different healthcare interventions, but also for making decisions about the prioritization and use of these interventions [5]. As a result, a summary of previous meta-analyses addressing the effects of MMT on HRQOL is now available to those interested in this topic and from which future research, practice and policy-making may be advanced. Second, the additional analyses conducted based on the available data (NNT, percentile improvement, PIs), aided in strengthening the evidence from which conclusions could be made from the included studies [38–47]. The inclusion of PIs also provides future researchers with data for assisting them in the planning and conduct of randomized controlled studies aimed at determining the effects of MMT on HRQOL in adults.

In addition to the strengths of the current study, at least three *possible* limitations were observed. First, the number of studies included in each meta-analysis was small and limited to very narrowly defined populations. Given the former, the strength of the evidence is less than ideal and may not be generalizable to other populations. The former notwithstanding, it’s important to note that two is the minimum number of studies necessary for conducting a meta-analysis [3]. However, the ability to generalize findings based on such a small number of studies is limited.

Second, it is possible that the results of the included meta-analyses suffered from small-study effects (publication bias, etc.). Unfortunately, the assessment of such was not possible since all of the meta-analysis included less than 10 effect sizes and a minimum of 10 is recommended before any such analyses is performed [35].

Third, biases common to the original meta-analyses, for example, ecologic fallacy and Simpson’s paradox, as well as the randomized controlled trials included in the meta-analyses, may have existed in the current investigation.

## Conclusions

The results of the current review suggest that MMT may improve HRQOL in adults with selected conditions. However, a need exists for a large meta-analysis of randomized controlled trials that is not limited to either the type of MMT or condition, and which also includes healthy adults.

## Supporting Information

S1 File Search strategies used for each database. This file includes the search strategies use for all of our electronic databases searches. These include PubMed, Sport Discus, Web of Science, Scopus, PsychInfo, Cochrane Database of Systematic Reviews, Physiotherapy Evidence Database, Database of Abstract Reviews of Effects and Proquest.
(DOCX)

S2 File Studies excluded, including reasons for exclusion. This file includes a list of all excluded studies, including the specific reasons for their exclusion.
(DOCX)

S1 Table Item by item results using the AMSTAR assessment instrument. This table includes the results of the AMSTAR assessment for each item from each study.
(DOCX)

S2 Table PRISMA Checklist. This table includes the Preferred Reporting Items for Systematic Reviews and Meta-Analysis (PRISMA) checklist relevant to this systematic review of previous systematic reviews with meta-analyses.
(DOC)

## References

[1] Zack MM (2013) Health-related quality of life—United States, 2006 and 2010. Morbidity and mortality weekly report Surveillance summaries (Washington, D C: 2002) 62 Suppl 3: 105–111. 16251867

[2] Barnes PM, Bloom B, Nahin RL (2008) Complementary and alternative medicine use among adults and children: United States, 2007. National health statistics reports 1–23. 19361005

[3] Higgins JPT, Green S (2011) Cochrane Handbook for Systematic Reviews of Interventions Version 5.1.0 [updated March 2011].

[4] Shorten A, Shorten B (2013) What is meta-analysis? Evid Based Nurs 16: 3–4. 10.1136/eb-2012-101118 23178310

[5] Smith V, Devane D, Begley CM, Clarke M (2011) Methodology in conducting a systematic review of systematic reviews of healthcare interventions. BMC Med Res Methodol 11: 15 10.1186/1471-2288-11-15 21291558 PMC3039637

[6] Johnson BT, MacDonald HV, Bruneau ML, Goldsby TU, Brown JC, Huedo-Medina TB, et al (2014) Methodological quality of meta-analyses on the blood pressure response to exercise: A review. J Hypertens 32: 706–723. 10.1097/HJH.0000000000000097 24463936

[7] Kelley GA, Kelley KS (2014) Effects of exercise on depressive symptoms in adults with arthritis and other rheumatic disease: a systematic review of meta-analyses. BMC Musculoskelet Disord 15: 121 10.1186/1471-2474-15-121 24708605 PMC4107718

[8] Liberati A, Altman DG, Tetzlaff J, Mulrow C, Gotzsche PC, Ioannidis JP, et al (2009) The PRISMA statement for reporting systematic reviews and meta-analyses of studies that evaluate health care interventions: explanation and elaboration. Ann Int Med 151: W65–W94. 19622512 10.7326/0003-4819-151-4-200908180-00136

[9] Sacks HS, Chalmers TC, Smith H (1982) Randomized versus historical controls for clinical trials. Am J Med 72: 233–240. 7058834 10.1016/0002-9343(82)90815-4

[10] Schulz KF, Chalmers I, Hayes R, Altman DG (1995) Empirical evidence of bias: Dimensions of methodological quality associated with estimates of treatment effects in controlled trials. JAMA 273: 408–412. 7823387 10.1001/jama.273.5.408

[11] Burnham JF (2006) Scopus database: a review. Biomed Digit Libr 3: 1–8. 16522216 10.1186/1742-5581-3-1 PMC1420322

[12] Lee E, Dobbins M, DeCorby K, Mcrae L, Tirilis D, Husson H (2012) An optimal search filter for retrieving systematic reviews and meta-analyses. BMC Med Res Methodol 12: 51 10.1186/1471-2288-12-51 22512835 PMC3515398

[13] (2009) Reference Manager, version 12.0.3 [computer program]. Philadelphia, PA: Thompson ResearchSoft.

[14] (2010) Microsoft Excel, version 2007 [computer program]. Redmond, WA: Microsoft Corporation.

[15] Cohen J (1968) Weighted kappa: nominal scale agreement with provision for scaled disagreement or partial credit. Psychol Bull 70: 213–220. 19673146 10.1037/h0026256

[16] Cicchetti DV, Sparrow SA (1981) Developing criteria for establishing interrater reliability of specific items: applications to assessment of adaptive behavior. Am J Ment Defic 86: 127–137. 7315877

[17] Shea BJ, Grimshaw JM, Wells GA, Boers M, Andersson N, Hamel C, et al (2007) Development of AMSTAR: a measurement tool to assess the methodological quality of systematic reviews. BMC Med Res Methodol 7: 10 17302989 10.1186/1471-2288-7-10 PMC1810543

[18] Shea BJ, Bouter LM, Peterson J, Boers M, Andersson N, Ortiz Z, et al (2007) External validation of a measurement tool to assess systematic reviews (AMSTAR). PLoS ONE 2: e1350 18159233 10.1371/journal.pone.0001350 PMC2131785

[19] Shea BJ, Hamel C, Wells GA, Bouter LM, Kristjansson E, Grimshaw J, et al (2009) AMSTAR is a reliable and valid measurement tool to assess the methodological quality of systematic reviews. J Clin Epidemiol 62: 1013–1020. 10.1016/j.jclinepi.2008.10.009 19230606

[20] Kang DY, Wu YX, Hu D, Hong Q, Wang JL, Zhang X (2012) Reliability and external validity of AMSTAR in assessing quality of TCM systematic reviews. Evid Based Complement Alternat Med 2012: 1–7. 10.1155/2012/732195 PMC3292204 22454679

[21] Oxman AD, Guyatt GH (1991) Validation of an index of the quality of review articles. J Clin Epidemiol 44: 1271–1278. 1834807 10.1016/0895-4356(91)90160-b

[22] Sacks HS, Berrier J, Reitman D, Ancona-Berk VA, Chalmers TC (1987) Meta-analysis of randomized controlled trials. N Engl J Med 316: 450–455. 3807986 10.1056/NEJM198702193160806

[23] Guyatt GH, Oxman AD, Vist GE, Kunz R, Falck-Ytter Y, Alonso-Coello P, et al (2008) GRADE: an emerging consensus on rating quality of evidence and strength of recommendations. BMJ 336: 924–926. 10.1136/bmj.39489.470347.AD 18436948 PMC2335261

[24] Harzing AW (2007) Harzing's Publish or Perish, version 4.4.7 [computer program].

[25] Mosteller F, Colditz GA (1996) Understanding research synthesis (meta-analysis). Ann Rev Public Health 1–23. 10.1146/annurev.pu.17.050196.000245 8724213

[26] Borenstein M, Hedges L, Higgins J, Rothstein H (2009) Introduction to meta-analysis West Sussex: John Wiley & Sons. 421 p.

[27] Cohen J (1992) A power primer. Psychol Bull 112: 155–159. 19565683 10.1037//0033-2909.112.1.155

[28] Higgins JPT, Thompson SG, Deeks JJ, Altman DG (2003) Measuring inconsistency in meta-analyses. BMJ 327: 557–560. 12958120 10.1136/bmj.327.7414.557 PMC192859

[29] Graham PL, Moran JL (2012) Robust meta-analytic conclusions mandate the provision of prediction intervals in meta-analysis summaries. J Clin Epidemiol 65: 503–510. 10.1016/j.jclinepi.2011.09.012 22265586

[30] Kelley GA, Kelley KS (2009) Impact of progressive resistance training on lipids and lipoproteins in adults: Another look at a meta-analysis using prediction intervals. Prev Med 49: 473–475. 10.1016/j.ypmed.2009.09.018 19804794

[31] Higgins JP, Thompson SG, Spiegelhalter DJ (2009) A re-evaluation of random-effects meta-analysis. J R Stat Soc Ser A 172: 137–159. 19381330 10.1111/j.1467-985X.2008.00552.x PMC2667312

[32] Ades AE, Caldwell DM, Reken S, Welton NJ, Sutton AJ, Dias S (2013) Evidence synthesis for decision making 7: A reviewers checklist. Med Decis Making 33: 679–691. 10.1177/0272989X13485156 23804511 PMC3704204

[33] Cohen J (1988) Statistical power analysis for the behavioral sciences New York: Academic Press.

[34] Durlak JA (2009) How to select, calculate, and interpret effect sizes. J Pediatr Psychol 34: 917–928. 10.1093/jpepsy/jsp004 19223279

[35] Sterne JA, Sutton AJ, Ioannidis JP, Terrin N, Jones DR, Lau J, et al (2011) Recommendations for examining and interpreting funnel plot asymmetry in meta-analyses of randomised controlled trials. BMJ 343: d4002 10.1136/bmj.d4002 21784880

[36] Egger M, Davey Smith G, Schneider M, Minder C (1997) Bias in meta-analysis detected by a simple graphical test. BMJ 315: 629–634. 9310563 10.1136/bmj.315.7109.629 PMC2127453

[37] Biostat. (2006) Comprehensive meta-analysis, version 2.2 [computer program]. Englewood, New Jersey.

[38] Buffart LM, van Uffelen JG, Riphagen II, Brug J, van MW, Brown WJ, et al (2012) Physical and psychosocial benefits of yoga in cancer patients and survivors, a systematic review and meta-analysis of randomized controlled trials. BMC Cancer 12: 559 10.1186/1471-2407-12-559 23181734 PMC3571972

[39] Cramer H, Lange S, Klose P, Paul A, Dobos G (2012) Yoga for breast cancer patients and survivors: a systematic review and meta-analysis. BMC Cancer 12: 412 10.1186/1471-2407-12-412 22988934 PMC3527138

[40] Cramer H, Lauche R, Klose P, Langhorst J, Dobos G (2013) Yoga for schizophrenia: a systematic review and meta-analysis. BMC Psychiatry 13: 32 10.1186/1471-244X-13-32 23327116 PMC3608162

[41] Cramer H, Lauche R, Haller H, Dobos G (2013) A Systematic review and meta-analysis of yoga for low back pain. Clin J Pain 29: 450–460. 10.1097/AJP.0b013e31825e1492 23246998

[42] Lin KY, Hu YT, Chang KJ, Lin HF, Tsauo JY (2011) Effects of yoga on psychological health, quality of life, and physical health of patients with cancer: a meta-analysis. Evid Based Complement Alternat Med 2011: 659876 10.1155/2011/659876 21437197 PMC3062158

[43] Pan L, Yan J, Guo Y, Yan J (2013) Effects of Tai Chi training on exercise capacity and quality of life in patients with chronic heart failure: a meta-analysis. Eur J Heart Fail 15: 316–323. 10.1093/eurjhf/hfs170 23099355

[44] Shneerson C, Taskila T, Gale N, Greenfield S, Chen YF (2013) The effect of complementary and alternative medicine on the quality of life of cancer survivors: a systematic review and meta-analyses. Complement Ther Med 21: 417–429. 10.1016/j.ctim.2013.05.003 23876573

[45] Wang F, Man JK, Lee EK, Wu T, Benson H, Fricchione GL, et al (2013) The effects of qigong on anxiety, depression, and psychological well-being: a systematic review and meta-analysis. Evid Based Complement Alternat Med 2013: 152738 10.1155/2013/152738 23401706 PMC3557628

[46] Zeng Y, Luo T, Xie H, Huang M, Cheng AS (2014) Health benefits of qigong or tai chi for cancer patients: a systematic review and meta-analyses. Complement Ther Med 22: 173–186. 10.1016/j.ctim.2013.11.010 24559833

[47] Zhang J, Yang KH, Tian JH, Wang CM (2012) Effects of yoga on psychologic function and quality of life in women with breast cancer: a meta-analysis of randomized controlled trials. J Altern Complement Med 18: 994–1002. 10.1089/acm.2011.0514 22909345

[48] Barrow DE, Bedford A, Ives G, O'Toole L, Channer KS (2007) An evaluation of the effects of Tai Chi Chuan and Chi Kung training in patients with symptomatic heart failure: a randomised controlled pilot study. Postgrad Med J 83: 717–721. 17989272 10.1136/pgmj.2007.061267 PMC2659967

[49] Yeh GY, Wood MJ, Lorell BH, Stevenson LW, Eisenberg DM, Wayne PM, et al (2004) Effects of tai chi mind-body movement therapy on functional status and exercise capacity in patients with chronic heart failure: a randomized controlled trial. Am J Med 117: 541–548. 15465501 10.1016/j.amjmed.2004.04.016

[50] Yeh GY, McCarthy EP, Wayne PM, Stevenson LW, Wood MJ, Forman D, et al (2011) Tai chi exercise in patients with chronic heart failure: a randomized clinical trial. Arch Int Med 171: 750–757. 10.1001/archinternmed.2011.150 21518942 PMC3277798

[51] Duijts SF, Faber MM, Oldenburg HS, van BM, Aaronson NK (2011) Effectiveness of behavioral techniques and physical exercise on psychosocial functioning and health-related quality of life in breast cancer patients and survivors—a meta-analysis. Psychooncology 20: 115–126. 10.1002/pon.1728 20336645

[52] Gorczynski P, Faulkner G (2010) Exercise therapy for schizophrenia. Cochrane Database Syst Rev. 10.1002/14651858.CD004412.pub2 PMC4164954 20464730

[53] Davies EJ, Moxham T, Rees K, Singh S, Coats AJ, Ebrahim S, et al (2010) Exercise training for systolic heart failure: Cochrane systematic review and meta-analysis. Eur J Heart Fail 12: 706–715. 10.1093/eurjhf/hfq056 20494922 PMC2891490

[54] Thomas D, Elliott E, Naughton G (2006) Exercise for type 2 diabetes mellitus. Cochrane Database Syst Rev Issue 3: CD002968 16855995 10.1002/14651858.CD002968.pub2 PMC8989410

[55] Conn VS, Hafdahl AR, Brown LM (2009) Meta-analysis of quality-of-life outcomes from physical activity interventions. Nurs Res 58: 175–183. 10.1097/NNR.0b013e318199b53a 19448521 PMC3159686

[56] Riley RD, Lambert PC, Abo-Zaid G (2010) Meta-analysis of individual participant data: rationale, conduct, and reporting. BMJ 340: c221 10.1136/bmj.c221 20139215

[57] Cooper H, Patall EA (2009) The relative benefits of meta-analysis conducted with individual participant data versus aggregated data. Psychol Methods 14: 165–176. 10.1037/a0015565 19485627

[58] Zeng Y, Luo T, Xie H, Huang M, Cheng AS (2014) Health benefits of qigong or tai chi for cancer patients: a systematic review and meta-analyses. Complement Ther Med 22: 173–186. 10.1016/j.ctim.2013.11.010 24559833

[59] Chandwani KD, Thornton B, Perkins GH, Arun B, Raghuram NV, Nagendra HR, et al (2010) Yoga improves quality of life and benefit finding in women undergoing radiotherapy for breast cancer. J Soc Integr Oncol 8: 43–55. 20388445

[60] Raghavendra RM, Nagarathna R, Nagendra HR, Gopinath KS, Srinath BS, Ravi BD, et al (2007) Effects of an integrated yoga programme on chemotherapy-induced nausea and emesis in breast cancer patients. Eur J Cancer Care 16: 462–474. 17944760 10.1111/j.1365-2354.2006.00739.x

