# Supplementary material for: Meditative Movement Therapies and Health-Related Quality-of-Life in Adults: A Systematic Review of Meta-Analyses
Source: PLoS One. 2015 Jun 8;10(6):e0129181. doi: 10.1371/journal.pone.0129181 (PMC4459806; doi:10.1371/journal.pone.0129181)
Supplement: S1 File — This file includes the search strategies use for all of our electronic databases searches. These include PubMed, Sport Discus, Web of Science, Scopus, PsychInfo, Cochrane Database of Systematic Reviews, Physiotherapy Evidence Database, Database of Abstract Reviews of Effects and Proquest. (DOCX) [file pone.0129181.s001.docx]

**File S1. Search strategies used for each database.**

PubMed

Sport Discus

Web of Science

Scopus

PsychInfo

Cochrane Database of Systematic Reviews

Physiotherapy Evidence Database (PEDRO)

Database of Abstract Reviews of Effects (DARE)

ProQuest
