# Supplementary material for: Meditative Movement Therapies and Health-Related Quality-of-Life in Adults: A Systematic Review of Meta-Analyses
Source: PLoS One. 2015 Jun 8;10(6):e0129181. doi: 10.1371/journal.pone.0129181 (PMC4459806; doi:10.1371/journal.pone.0129181)
Supplement: S2 File — This file includes a list of all excluded studies, including the specific reasons for their exclusion. (DOCX) [file pone.0129181.s002.docx]

**File S2. Studies excluded, including reasons for exclusion.**

(1) MEDITATION RESEARCH SUMMARIES. Yoga Ther Pract 2006 December;2(4):3-6. Inappropriate Study Design
Ref ID: 411

(2) Yoga aids breast cancer patients and lowers hypertension. Research Review (International Council on Active Aging) 2007 September 5;7(32):3. Inappropriate Study Design
Ref ID: 413

(3) Physical activity improves quality of life during menopause. Research Review (International Council on Active Aging) 2007 May 2;7(15):3-4. Inappropriate Study Design
Ref ID: 412

(4) Abbott RB, Hui KK, Hays RD, Li MD, Pan T. A randomized controlled trial of tai chi for tension headaches. Evid Based Complement Alternat Med 2007 March;4(1):107-13. Inappropriate Study Design
Ref ID: 239

(5) Abrantes AM, Friedman JH, Brown RA, Strong DR, Desaulniers J, Ing E, Saritelli J, Riebe D. Physical activity and neuropsychiatric symptoms of Parkinson disease. J Geriatr Psychiatry Neurol 2012;25(3):138-45. Inappropriate Study Design
Ref ID: 439

(6) Afonso RF, Hachul H, Kozasa EH, Oliveira DD, Goto V, Rodrigues D, Tufik S, Leite JR. Yoga decreases insomnia in postmenopausal women: a randomized clinical trial. Menopause 2012;19(2):186-93. Inappropriate Study Design
Ref ID: 440

(7) Agarwal BB. Do dietary spices impair the patient-reported outcomes for stapled hemorrhoidopexy? A randomized controlled study. Surg Endosc 2011 May;25(5):1535-40. Inappropriate Outcomes
Ref ID: 159

(8) Ahmadi A, Nikbakh M, Arastoo AA, Habibi AH. The effects of a yoga intervention on balance, speed and endurance of walking, fatigue and quality of life in people with multiple sclerosis. J Hum Kinet 2010;23:71-8. Inappropriate Study Design
Ref ID: 441

(9) Ahmadi A, Arastoo AA, Nikbakht M, Zahednejad S, Rajabpour M. Comparison of the effect of 8 weeks aerobic and yoga training on ambulatory function, fatigue and mood status in MS patients. Iran Red Crescent Med J 2013;15(6):449-54. Inappropriate Study Design
Ref ID: 442

(10) Al-Azri M, Al-Awisi H, Al-Moundhri M. Coping with a diagnosis of breast cancer-Literature review and implications for developing countries. Breast J 2009;15(6):615-22. Inappropriate Outcomes
Ref ID: 443

(11) Albrecht TA, Taylor AG. Physical activity in patients with advanced-stage cancer: a systematic review of the literature. Clin J Oncol Nurs 2012 June 1;16(3):293-300. Inappropriate Outcomes
Ref ID: 91

(12) Alcantara-Silva TRD, Freitas R, Freitas NMA, Machado GDP. Fatigue related to radiotherapy for breast and/or gynaecological cancer: a systematic review. J Clin Nurs 2013;22(19-20):2679-86. Inappropriate Intervention
Ref ID: 444

(13) Allen LB, Tsao JCI, Hayes LP, Zeltzer LK. Peer mentorship to promote effective pain management in adolescents: study protocol for a randomised controlled trial. Trials 2011;12. Inappropriate Population
Ref ID: 445

(14) Alp A, Cansever S, Gorgec N, Yurtkuran M, Topsac T. Effects of tai chi exercise on functional and life quality assessments in senile osteoporosis. Turkiye Klinikleri Tip Bilimleri Dergisi 2009;29(3):687-95. Inappropriate Study Design
Ref ID: 446

(15) Alrwaily M, Whitney SL. Vestibular rehabilitation of older adults with dizziness. Otolaryngol Clin North Am 2011;44(2):473-+. Inappropriate Intervention
Ref ID: 447

(16) Amano S, Nocera JR, Vallabhajosula S, Juncos JL, Gregor RJ, Waddell DE, Wolf SL, Hass CJ. The effect of Tai Chi exercise on gait initiation and gait performance in persons with Parkinson's disease. Parkinsonism Relat Disord 2013;19(11):955-60. Inappropriate Study Design
Ref ID: 448

(17) Andersen SR, Wurtzen H, Steding-Jessen M, Christensen J, Andersen KK, Flyger H, Mitchelmore C, Johansen C, Dalton SO. Effect of mindfulness-based stress reduction on sleep quality: Results of a randomized trial among Danish breast cancer patients. Acta Oncologica 2013;52(2):336-44. Inappropriate Study Design
Ref ID: 449

(18) Arias AJ, Steinberg K, Banga A, Trestman RL. Systematic review of the efficacy of meditation techniques as treatments for medical illness. J Altern Complement Med 2006;12(8):817-32. Inappropriate Study Design
Ref ID: 450

(19) Arko D. Treatment possibilities of menopausal symptoms in breast cancer patients. Zdravniski Vestnik-Slovenian Med J 2009;78:201-5. Inappropriate Outcomes
Ref ID: 451

(20) Audette JF, Jin YS, Newcomer R, Stein L, Duncan G, Frontera WR. Tai Chi versus brisk walking in elderly women. Age Ageing 2006;35(4):388-93. Inappropriate Study Design
Ref ID: 452

(21) Babbar S, Parks-Savage AC, Chauhan SP. Yoga during pregnancy: a review. Am J Perinatol 2012 June;29(6):459-64. Inappropriate Study Design
Ref ID: 101

(22) Balk J, Bernardo LM. Using yoga to promote bone health and reduce fracture risk in the geriatric population. Top Geriatr Rehabil 2011;27(2):116-23. Inappropriate Study Design
Ref ID: 454

(23) Balk J. Yoga for quality of life and balance in postmenopausal osteoporosis. Altern Med Alert 2010 October;13(10):113-5. Inappropriate Study Design
Ref ID: 414

(24) Banasik J, Williams H, Haberman M, Blank SE, Bendel R. Effect of Iyengar yoga practice on fatigue and diurnal salivary cortisol concentration in breast cancer survivors. J Am Acad Nurse Pract 2011;23(3):135-42. Inappropriate Study Design
Ref ID: 455

(25) Barbat-Artigas S, Filion ME, Dupontgand S, Karelis AD, Aubertin-Leheudre M. Effects of tai chi training in dynapenic and nondynapenic postmenopausal women. Menopause 2011;18(9):974-9. Inappropriate Study Design
Ref ID: 456

(26) Barrow DE, Bedford A, Ives G, O'Toole L, Channer KS. An evaluation of the effects of tai chi chuan and chi kung training in patients with symptomatic heart failure: a randomised controlled pilot study. Postgrad Med J 2007 November;83(985):717-21. Inappropriate Study Design
Ref ID: 226

(27) Benito-Leon J, Morales JM, Rivera-Navarro J, Mitchell AJ. A review about the impact of multiple sclerosis on health-related quality of life. Disabil Rehabil 2003;25(22):1291-303. Inappropriate Intervention
Ref ID: 360

(28) Bennell KL, Hinman RS. A review of the clinical evidence for exercise in osteoarthritis of the hip and knee. J Sci Med Sport 2011;14(1):4-9. Inappropriate Study Design
Ref ID: 459

(29) Bernardi MLD, Amorim MHC, Zandonade E, Santaella DF, Barbosa JDN. The effects of hatha yoga exercises on stress and anxiety levels in mastectomized women. Cien Saude Colet 2013;18(12):3621-32. Inappropriate Study Design
Ref ID: 460

(30) Bidwell AJ, Yazel B, Davin D, Fairchild TJ, Kanaley JA. Yoga training improves quality of life in women with asthma. J Altern Complement Med 2012 August;18(8):749-55. Inappropriate Study Design
Ref ID: 85

(31) Blom K, How M, Dai M, Baker B, Irvine J, Abbey S, Abramson BL, Myers M, Perkins N, Tobe SW. Hypertension Analysis of stress Reduction using Mindfulness meditation and Yoga (The HARMONY Study): Study protocol of a randomised control trial. BMJ Open 2012;2(2). Inappropriate Study Design
Ref ID: 462

(32) Blumenthal JA, Emery CF, Madden DJ, George LK, Coleman RE, Riddle MW, McKee DC, Reasoner J, Williams RS. Cardiovascular and behavioral effects of aerobic exercise training in healthy older men and women. J Gerontol 1989 September;44(5):M147-M157. Inappropriate Study Design
Ref ID: 280

(33) Boehm K, Ostermann T, Milazzo S, Bussing A. Effects of yoga interventions on fatigue: A meta-analysis. Evid Based Complement Alternat Med 2012. Inappropriate Outcomes
Ref ID: 463

(34) Booth-Laforce C, Thurston RC, Taylor MR. A pilot study of a Hatha yoga treatment for menopausal symptoms. Maturitas 2007;57(3):286-95. Inappropriate Study Design
Ref ID: 464

(35) Bower JE, Garet D, Sternlieb B. Yoga for persistent fatigue in breast cancer survivors: results of a pilot study. Evid Based Complement Alternat Med 2011;2011:623168. Inappropriate Study Design
Ref ID: 146

(36) Bremander A, Bergman S. Non-pharmacological management of musculoskeletal disease in primary care. Best Pract Res Clin Rheumatol 2008;22(3):563-77. Inappropriate Intervention
Ref ID: 467

(37) Brotto LA, Mehak L, Kit C. Yoga and sexual functioning: A review. J Sex Marital Ther 2009;35(5):378-90. Inappropriate Study Design
Ref ID: 468

(38) Bruton A, Thomas M. The role of breathing training in asthma management. Curr Opin Allergy Clin Immunol 2011;11(1):53-7. Inappropriate Outcomes
Ref ID: 469

(39) Burgess J, Ekanayake B, Lowe A, Dunt D, Thien F, Dharmage SC. Systematic review of the effectiveness of breathing retraining in asthma management. Expert Rev Respir Med 2011;5(6):789-807. Inappropriate Outcomes
Ref ID: 471

(40) Burini D, Farabollini B, Iacucci S, Rimatori C, Riccardi G, Capecci M, Provinciali L, Ceravolo MG. A randomised controlled cross-over trial of aerobic training versus Qigong in advanced Parkinson's disease. Eura Medicophys 2006 September;42(3):231-8. Inappropriate Study Design
Ref ID: 243

(41) Busch AJ, Webber SC, Brachaniec M, Bidonde J, Dal Bello-Haas V, Danyliw AD, Overend TJ, Richards RS, Sawant A, Schachter CL. Exercise therapy for fibromyalgia. Curr Pain Headache Rep 2011;15(5):358-67. Inappropriate Outcomes
Ref ID: 472

(42) Bussing A, Michalsen A, Khalsa SB, Telles S, Sherman KJ. Effects of yoga on mental and physical health: a short summary of reviews. Evid Based Complement Alternat Med 2012;2012:165410. Inappropriate Study Design
Ref ID: 282

(43) Cade WT, Reeds DN, Mondy KE, Overton ET, Grassino J, Tucker S, Bopp C, Laciny E, Hubert S, Lassa-Claxton S, Yarasheski KE. Yoga lifestyle intervention reduces blood pressure in HIV-infected adults with cardiovascular disease risk factors*. HIV Med 2010;11(6):379-88. Inappropriate Study Design
Ref ID: 474

(44) Cadmus-Bertram L, Littman AJ, Ulrich CM, Stovall R, Ceballos RM, McGregor BA, Wang CY, Ramaprasad J, McTiernan A. Predictors of adherence to a 26-week viniyoga intervention among post-treatment breast cancer survivors. J Altern Complement Med 2013 September;19(9):751-8. Inappropriate Study Design
Ref ID: 283

(45) Cadore EL, Rodriguez-Manas L, Sinclair A, Izquierdo M. Effects of different exercise interventions on risk of falls, gait ability, and balance in physically frail older adults: A systematic review. Rejuvenation Res 2013;16(2):105-14. Inappropriate Outcomes
Ref ID: 475

(46) Caminiti G, Volterrani M, Marazzi G, Cerrito A, Massaro R, Arisi A, Franchini A, Sposato B, Rosano G. Tai chi enhances the effects of endurance training in the rehabilitation of elderly patients with chronic heart failure. Rehabil Res Pract 2011;2011:761958. Inappropriate Study Design
Ref ID: 110

(47) Campo RA, O'Connor K, Light KC, Nakamura Y, Lipschitz DL, Lastayo PC, Pappas L, Boucher K, Irwin MR, Agarwal N, Kinney AY. Feasibility and acceptability of a tai chi chih randomized controlled trial in senior female cancer survivors. Integr Cancer Ther 2013 November;12(6):464-74. Inappropriate Study Design
Ref ID: 284

(48) Campo RA, Agarwal N, Lastayo PC, O'Connor K, Pappas L, Boucher KM, Gardner J, Smith S, Light KC, Kinney AY. Levels of fatigue and distress in senior prostate cancer survivors enrolled in a 12-week randomized controlled trial of qigong. J Cancer Surviv 2014 March;8(1):60-9. Inappropriate Study Design
Ref ID: 285

(49) Carayol M, Bernard P, Boiche J, Riou F, Mercier B, Cousson-Gelie F, Romain AJ, Delpierre C, Ninot G. Psychological effect of exercise in women with breast cancer receiving adjuvant therapy: what is the optimal dose needed? Ann Oncol 2013;24(2):291-300. Inappropriate Outcomes
Ref ID: 477

(50) Carbonell-Baeza A, Romero A, Aparicio VA, Ortega FB, Tercedor P, Delgado-Fernandez M, Ruiz JR. Preliminary findings of a 4-month tai chi intervention on tenderness, functional capacity, symptomatology, and quality of life in men with fibromyalgia. Am J Mens Health 2011;5(5):421-9. Inappropriate Study Design
Ref ID: 478

(51) Carlson LE, Bultz BD. Mind-body interventions in oncology. Curr Treat Options Oncol 2008 June;9(2-3):127-34. Inappropriate Study Design
Ref ID: 212

(52) Carlson LE, Doll R, Stephen J, Faris P, Tamagawa R, Drysdale E, Speca M. Randomized controlled trial of Mindfulness-based cancer recovery versus supportive expressive group therapy for distressed survivors of breast cancer. J Clin Oncol 2013 September 1;31(25):3119-26. Inappropriate Study Design
Ref ID: 286

(53) Carson JW, Carson KM, Porter LS, Keefe FJ, Shaw H, Miller JM. Yoga for women with metastatic breast cancer: Results from a pilot study. J Pain Symptom Manage 2007;33(3):331-41. Inappropriate Study Design
Ref ID: 480

(54) Carson JW, Carson KM, Porter LS, Keefe FJ, Seewaldt VL. Yoga of awareness program for menopausal symptoms in breast cancer survivors: results from a randomized trial. Support Care Cancer 2009 October;17(10):1301-9. Inappropriate Study Design
Ref ID: 203

(55) Carson JW, Carson KM, Jones KD, Mist SD, Bennett RM. Follow-up of yoga of awareness for fibromyalgia: results at 3 months and replication in the wait-list group. Clin J Pain 2012 November;28(9):804-13. Inappropriate Study Design
Ref ID: 87

(56) Casden DR. The effects of Ashtanga yoga on autonomic, respiratory and cognitive functioning; psychological symptoms and somatic complaints: A controlled study. US: ProQuest Information & Learning; 2005.

Inappropriate Study Design
Ref ID: 34

(57) Cassileth BR, Gubili J, Yeung KS. Integrative medicine: complementary therapies and supplements. Nat Rev Urol 2009;6(4):228-33. Inappropriate Study Design
Ref ID: 481

(58) Chan AW, Lee A, Suen LK, Tam WW. Effectiveness of a tai chi qigong program in promoting health-related quality of life and perceived social support in chronic obstructive pulmonary disease clients. Qual Life Res 2010 June;19(5):653-64. Inappropriate Study Design
Ref ID: 175

(59) Chan AW, Lee A, Lee DT, Sit JW, Chair SY. Evaluation of the sustaining effects of tai chi qigong in the sixth month in promoting psychosocial health in COPD patients: a single-blind, randomized controlled trial. Scientif World J 2013;2013:425082. Inappropriate Study Design
Ref ID: 287

(60) Chan AWK, Lee A, Lee DTF, Suen LKP, Tam WWS, Chair SY, Griffiths P. The sustaining effects of tai chi qigong on physiological health for COPD patients: A randomized controlled trial. Complement Ther Med 2013;21(6):585-94. Inappropriate Study Design
Ref ID: 483

(61) Chan CL, Wang CW, Ho RT, Ho AH, Ziea ET, Taam Wong VC, Ng SM. A systematic review of the effectiveness of qigong exercise in cardiac rehabilitation. Am J Chin Med 2012;40(2):255-67. Inappropriate Outcomes
Ref ID: 100

(62) Chan CLW, Wang CW, Ho RTH, Ng SM, Chan JSM, Ziea ETC, Wong VCW. A systematic review of the effectiveness of qigong exercise in supportive cancer care. Support Care Cancer 2012;20(6):1121-33. Inappropriate Study Design
Ref ID: 485

(63) Chan W, Immink MA, Hillier S. Yoga and exercise for symptoms of depression and anxiety in people with poststroke disability: a randomized, controlled pilot trial. Altern Ther Health Med 2012 May;18(3):34-43. Inappropriate Study Design
Ref ID: 80

(64) Chan WK. evaluation of a tai chi qigong program in promoting physiological and psychosocial health statuses in chronic obstructive pulmonary disease clients. Ann Arbor: The Chinese University of Hong Kong (Hong Kong); 2011.

Inappropriate Study Design
Ref ID: 26

(65) Chandwani KD, Thornton B, Perkins GH, Arun B, Raghuram NV, Nagendra HR, Wei Q, Cohen L. Yoga improves quality of life and benefit finding in women undergoing radiotherapy for breast cancer. J Soc Integr Oncol 2010;8(2):43-55. Inappropriate Study Design
Ref ID: 173

(66) Chandwani KD, Ryan JL, Peppone LJ, Janelsins MM, Sprod LK, Devine K, Trevino L, Gewandter J, Morrow GR, Mustian KM. Cancer-related stress and complementary and alternative medicine: A review. Evid Based Complement Alternat Med 2012. Inappropriate Study Design
Ref ID: 488

(67) Chard SE, Stuart M. An ecological perspective on the community translation of exercise research for older adults. J Appl Gerontol 2012;31(1):28-51. Inappropriate Study Design
Ref ID: 489

(68) Cheema BS, Marshall PW, Chang D, Colagiuri B, Machliss B. Effect of an office worksite-based yoga program on heart rate variability: A randomized controlled trial. BMC Public Health 2011;11. Inappropriate Study Design
Ref ID: 490

(69) Cheema BS, Houridis A, Busch L, Raschke-Cheema V, Melville GW, Marshall PW, Chang D, Machliss B, Lonsdale C, Bowman J, Colagiuri B. Effect of an office worksite-based yoga program on heart rate variability: Outcomes of a randomized controlled trial. BMC Complement Altern Med 2013;13:82. Inappropriate Study Design
Ref ID: 288

(70) Chen KM, Chen MH, Lin MH, Fan JT, Lin HS, Li CH. Effects of yoga on sleep quality and depression in elders in assisted living facilities. J Nurs Res 2010 March;18(1):53-61. Inappropriate Study Design
Ref ID: 176

(71) Chen KW, Berger CC, Manheimer E, Forde D, Magidson J, Dachman L, Lejuez CW. meditative therapies for reducing anxiety: a systematic review and meta-analysis of randomized controlled trials. Depress Anxiety 2012;29(7):545-62. Inappropriate Outcomes
Ref ID: 493

(72) Chi I, Jordan-Marsh M, Guo M, Xie B, Bai ZG. Tai chi and reduction of depressive symptoms for older adults: A meta-analysis of randomized trials. Geriatr Gerontol Int 2013;13(1):3-12. Inappropriate Outcomes
Ref ID: 495

(73) Choi JY. Complementary therapy for improvement of quality of life in cancer patients. J Korean Am Med Assoc 2008;51(5):435-48. Inappropriate Study Design
Ref ID: 496

(74) Chou CH, Hwang CL, Wu YT. Effect of exercise on physical function, daily living activities, and quality of life in the frail older adults: a meta-analysis. Arch Phys Med Rehabil 2012;93(2):237-44. Inappropriate Intervention
Ref ID: 497

(75) Chow YWY, Tsang HWH. Biopsychosocial effects of qigong as a mindful exercise for people with anxiety disorders: A speculative review. J Altern Complement Med 2007;13(8):831-9. Inappropriate Study Design
Ref ID: 498

(76) Chow YWY, Dorcas A, Siu AMH. The effects of qigong on reducing stress and anxiety and enhancing body mind well-being. Mindfulness 2012 March;3(1):51-9. Inappropriate Study Design
Ref ID: 35

(77) Chow YWY. The effects of qigong on reducing stress, anxiety and enhancing body-mind wellbeing. US: ProQuest Information & Learning; 2013.

Inappropriate Study Design
Ref ID: 36

(78) Chuang LH, Soares MO, Tilbrook H, Cox H, Hewitt CE, Aplin J, Semlyen A, Trewhela A, Watt I, Torgerson DJ. A pragmatic multicentered randomized controlled trial of yoga for chronic low back pain: economic evaluation. Spine 2012 August 15;37(18):1593-601. Inappropriate Study Design
Ref ID: 99

(79) Chung SC. Health related quality of life in clinical studies for chronic diseases-Design and analytical considerations. US: ProQuest Information & Learning; 2011.

Inappropriate Study Design
Ref ID: 47

(80) Church J, Goodall S, Norman R, Haas M. The cost-effectiveness of falls prevention interventions for older community-dwelling Australians. Aust N Z J Public Health 2012;36(3):241-8. Inappropriate Outcomes
Ref ID: 500

(81) Chyu MC, James CR, Sawyer SF, Brismee JM, Xu KT, Poklikuha G, Dunn DM, Shen CL. Effects of tai chi exercise on posturography, gait, physical function and quality of life in postmenopausal women with osteopaenia: a randomized clinical study. Clin Rehabil 2010;24(12):1080-90. Inappropriate Study Design
Ref ID: 501

(82) Chyu MC, von B, V, Brismee JM, Zhang Y, Yeh JK, Shen CL. Complementary and alternative exercises for management of osteoarthritis. Arthritis 2011;2011:364319. Inappropriate Study Design
Ref ID: 117

(83) Clark PG, Cortese-Jimenez G, Cohen E. Effects of reiki, yoga, or meditation on the physical and psychological symptoms of chemotherapy-induced peripheral neuropathy: A randomized pilot study. J Evid Based Complementary Altern Med 2012 October;17(3):161-71. Inappropriate Study Design
Ref ID: 49

(84) Cohen L, Chen Z, Arun B, Shao ZM, Dryden M, Xu LH, Le-Petross C, Dogan B, McKenna BJ, Markman M, Babiera G. External qigong therapy for women with breast cancer prior to surgery. Integr Cancer Ther 2010;9(4):348-53. Inappropriate Study Design
Ref ID: 503

(85) Comans TA, Brauer SG, Haines TP. Randomized trial of domiciliary versus center-based rehabilitation: which is more effective in reducing falls and improving quality of life in older fallers? J Gerontol A Biol Sci Med Sci 2010;65(6):672-9. Inappropriate Study Design
Ref ID: 504

(86) Coote S, Garrett M, Hogan N, Larkin A, Saunders J. Getting the balance right: a randomised controlled trial of physiotherapy and exercise interventions for ambulatory people with multiple sclerosis. BMC Neurol 2009;9. Inappropriate Study Design
Ref ID: 505

(87) Cote A, Daneault S. Effect of yoga on patients with cancer: Our current understanding. Can Fam Physician 2012;58(9):E475-E479. Inappropriate Study Design
Ref ID: 506

(88) Craft LL, VanIterson EH, Helenowski IB, Rademaker AW, Courneya KS. Exercise effects on depressive symptoms in cancer survivors: A systematic review and meta-analysis. Cancer Epidemiol Biomarkers Prev 2012;21(1):3-19. Inappropriate Outcomes
Ref ID: 507

(89) Cramer H, Lauche R, Hohmann C, Langhorst J, Dobos G. Yoga for chronic neck pain: a 12-month follow-up. Pain Med 2013 April;14(4):541-8. Inappropriate Study Design
Ref ID: 293

(90) Cramer H, Lauche R, Haller H, Langhorst J, Dobos G, Berger B. "I'm more in balance": a qualitative study of yoga for patients with chronic neck pain. J Altern Complement Med 2013 June;19(6):536-42. Inappropriate Study Design
Ref ID: 294

(91) Cramer H, Lauche R, Hohmann C, Ludtke R, Haller H, Michalsen A, Langhorst J, Dobos G. Randomized-controlled trial comparing yoga and home-based exercise for chronic neck pain. Clin J Pain 2013 March;29(3):216-23. Inappropriate Study Design
Ref ID: 296

(92) Cramer H, Lauche R, Langhorst J, Dobos G. Yoga for depression: A systematic review and meta-analysis. Depress Anxiety 2013 November;30(11):1068-83. Inappropriate Outcomes
Ref ID: 292

(93) Cramer H, Lauche R, Haller H, Dobos G, Michalsen A. A systematic review of yoga for heart disease. Eur J Prev Cardiol 2014 February 3. Inappropriate Study Design
Ref ID: 298

(94) Cramer H, Lauche R, Hohmann C, Langhorst J, Dobos G. Yoga for chronic neck pain: A 12-month follow-up. Pain Med 2013 April;14(4):541-8. Inappropriate Study Design
Ref ID: 416

(95) Craske NJ, Turner W, Zammit-Maempe J, Lee MS. Qigong ameliorates symptoms of chronic fatigue: a pilot uncontrolled study. Evid Based Complement Alternat Med 2009 June;6(2):265-70. Inappropriate Study Design
Ref ID: 208

(96) Culos-Reed SN, Carlson LE, Daroux LM, Hately-Aldous S. A pilot study of yoga for breast cancer survivors: physical and psychological benefits. Psychooncology 2006 October;15(10):891-7. Inappropriate Study Design
Ref ID: 251

(97) Culos-Reed SN, Mackenzie MJ, Sohl SJ, Jesse MT, Zahavich ANR, Danhauer SC. Yoga & cancer interventions: A review of the clinical significance of patient reported outcomes for cancer survivors. Evid Based Complement Alternat Med 2012. Inappropriate Study Design
Ref ID: 516

(98) Curtis K, Weinrib A, Katz J. Systematic review of yoga for pregnant women: current status and future directions. Evid Based Complement Alternat Med 2012;2012:715942. Inappropriate Study Design
Ref ID: 299

(99) Daley A, Stokes-Lampard H, Macarthur C. Exercise for vasomotor menopausal symptoms. Cochrane Database Syst Rev 2011;(5). Inappropriate Outcomes
Ref ID: 518

(100) Danhauer SC, Mihalko SL, Russell GB, Campbell CR, Felder L, Daley K, Levine EA. Restorative yoga for women with breast cancer: findings from a randomized pilot study. Psychooncology 2009 April;18(4):360-8. Inappropriate Study Design
Ref ID: 199

(101) Danucalov MA, Kozasa EH, Ribas KT, Galduroz JC, Garcia MC, Verreschi IT, Oliveira KC, Romani de OL, Leite JR. A yoga and compassion meditation program reduces stress in familial caregivers of Alzheimer's disease patients. Evid Based Complement Alternat Med 2013;2013:513149. Inappropriate Study Design
Ref ID: 300

(102) Davidoff J, Christensen S, Khalili DN, Nguyen J, Ishak WW. Quality of life in panic disorder: looking beyond symptom remission. Qual Life Res 2012;21(6):945-59. Inappropriate Study Design
Ref ID: 522

(103) Day L, Hill KD, Jolley D, Cicuttini F, Flicker L, Segal L. Impact of tai chi on impairment, functional limitation, and disability among preclinically disabled older people: a randomized controlled trial. Arch Phys Med Rehabil 2012 August;93(8):1400-7. Inappropriate Study Design
Ref ID: 98

(104) Dechamps A, Onifade C, Decamps A, Bourdel-Marchasson I. Health-related quality of life in frail institutionalized elderly: Effects of a cognition-action intervention and tai chi. J Aging Phys Act 2009;17(2):236-48. Inappropriate Study Design
Ref ID: 524

(105) Dechamps A, Diolez P, Thiaudiere E, Tulon A, Onifade C, Vuong T, Helmer C, Bourdel-Marchasson I. Effects of exercise programs to prevent decline in health-related quality of life in highly deconditioned institutionalized elderly persons: A randomized controlled trial. Arch Intern Med 2010 January 25;170(2):162-9. Inappropriate Study Design
Ref ID: 181

(106) Devereux K, Robertson D, Briffa NK. Effects of a water-based program on women 65 years and over: A randomised controlled trial. Aust J Physiother 2005;51(2):102-8. Inappropriate Study Design
Ref ID: 526

(107) Dhruva A, Miaskowski C, Abrams D, Acree M, Cooper B, Goodman S, Hecht FM. Yoga breathing for cancer chemotherapy-associated symptoms and quality of life: results of a pilot randomized controlled trial. J Altern Complement Med 2012 May;18(5):473-9. Inappropriate Study Design
Ref ID: 95

(108) Ding M. Tai Chi for stroke rehabilitation: a focused review. Am J Phys Med Rehabil 2012 December;91(12):1091-6. Inappropriate Study Design
Ref ID: 301

(109) Ding M, Zhang W, Li K, Chen X. Effectiveness of tai chi and qigong on chronic obstructive pulmonary disease: A systematic review and meta-analysis. J Altern Complement Med 2014 February;20(2):79-86. Inappropriate Study Design
Ref ID: 302

(110) Donesky-Cuenco D, Nguyen HQ, Paul S, Carrieri-Kohlman V. Yoga therapy decreases dyspnea-related distress and improves functional performance in people with chronic obstructive pulmonary disease: a pilot study. J Altern Complement Med 2009 March;15(3):225-34. Inappropriate Study Design
Ref ID: 198

(111) Donesky D, Melendez M, Nguyen HQ, Carrieri-Kohlman V. A responder analysis of the effects of yoga for individuals with COPD: who benefits and how? Int J Yoga Therap 2012;(22):23-36. Inappropriate Study Design
Ref ID: 303

(112) Doulatabad SN, Nooreyan K, Doulatabad AN, Noubandegani ZM. The effects of pranayama, hatha and raja yoga on physical pain and the quality of life of women with multiple sclerosis. Afr J Tradit Complement Altern Med 2012;10(1):49-52. Inappropriate Study Design
Ref ID: 108

(113) Duraiswamy G, Thirthalli J, Nagendra HR, Gangadhar BN. Yoga therapy as an add-on treatment in the management of patients with schizophrenia--a randomized controlled trial. Acta Psychiatr Scand 2007 September;116(3):226-32. Inappropriate Study Design
Ref ID: 232

(114) Ebben MR, Narizhnaya M. Cognitive and behavioral treatment options for insomnia. Mt Sinai J Med 2012;79(4):512-23. Inappropriate Outcomes
Ref ID: 532

(115) Ebnezar J, Nagarathna R, Bali Y, Nagendra HR. Effect of an integrated approach of yoga therapy on quality of life in osteoarthritis of the knee joint: A randomized control study. Int J Yoga 2011 July;4(2):55-63. Inappropriate Study Design
Ref ID: 122

(116) Eichenberger PA, Diener SN, Kofmehl R, Spengler CM. Effects of exercise training on airway hyperreactivity in asthma: a systematic review and meta-analysis. Sports Med 2013 November;43(11):1157-70. Inappropriate Intervention
Ref ID: 304

(117) El-Khoury F, Cassou B, Charles MA, Dargent-Molina P. The effect of fall prevention exercise programmes on fall induced injuries in community dwelling older adults: systematic review and meta-analysis of randomised controlled trials. Br Med J (Clin Res Ed) 2013;347. Inappropriate Outcomes
Ref ID: 534

(118) Elavsky S, McAuley E. Physical activity and mental health outcomes during menopause: a randomized controlled trial. Ann Behav Med 2007 April;33(2):132-42. Inappropriate Study Design
Ref ID: 237

(119) Elavsky S. Physical activity, menopause, and quality of life: the role of affect and self-worth across time. Menopause 2009 March;16(2):265-71. Inappropriate Study Design
Ref ID: 204

(120) Elavsky S. Physical activity and quality of life during menopause. Ann Arbor: University of Illinois at Urbana-Champaign; 2006.

Inappropriate Study Design
Ref ID: 27

(121) Elinoff V, Lynn SJ, Ochiai H, Hallquist M. The efficacy of kiko exercises on the prevention of migraine headaches: a pilot study. Am J Chin Med 2009;37(3):459-70. Inappropriate Study Design
Ref ID: 192

(122) Elkins G, Fisher W, Johnson A. Mind-body therapies in integrative oncology. Curr Treat Options Oncol 2010;11(3-4):128-40. Inappropriate Study Design
Ref ID: 538

(123) Escalante Y, Saavedra JM, Garcia-Hermoso A, Silva AJ, Barbosa TM. Physical exercise and reduction of pain in adults with lower limb osteoarthritis: A systematic review. J Back Musculoskelet Rehabil 2010;23(4):175-86. Inappropriate Study Design
Ref ID: 539

(124) Esch T, Duckstein J, Welke J, Stefano GB, Braun V. Mind/body techniques for physiological and psychological stress reduction: Stress management via tai chi training - a pilot study. Med Sci Monit2007;13(11):CR488-CR497. Inappropriate Study Design
Ref ID: 540

(125) Finnegan-John J, Molassiotis A, Richardson A, Ream E. A systematic review of complementary and alternative medicine interventions for the management of cancer-related fatigue. Integr Cancer Ther 2013;12(4):276-90. Inappropriate Study Design
Ref ID: 543

(126) Flegal KE, Kishiyama S, Zajdel D, Haas M, Oken BS. Adherence to yoga and exercise interventions in a 6-month clinical trial. BMC Complement Altern Med 2007;7:37. Inappropriate Study Design
Ref ID: 225

(127) Forbes D, Forbes S, Morgan DG, Markle-Reid M, Wood J, Culum I. Physical activity programs for persons with dementia. Cochrane Database Syst Rev 2008;(3):CD006489. Inappropriate Outcomes
Ref ID: 214

(128) Forbes D, Thiessen EJ, Blake CM, Forbes SC, Forbes S. Exercise programs for people with dementia. Cochrane Database Syst Rev 2013;12:CD006489. Inappropriate Outcomes
Ref ID: 305

(129) Forster A, Lambley R, Hardy J, Young J, Smith J, Green J, Burns E. Rehabilitation for older people in long-term care. Cochrane Database Syst Rev 2009;(1). Inappropriate Outcomes
Ref ID: 367

(130) Freitas DA, Holloway EA, Bruno SS, Chaves-Gabriela SS, Fregonezi-Guilherme AF, Mendonça-Karla MPP. Breathing exercises for adults with asthma. Cochrane Database Syst Rev 2013. Inappropriate Intervention
Ref ID: 9

(131) Frick KD, Kung JY, Parrish JM, Narrett MJ. Evaluating the cost-effectiveness of fall prevention programs that reduce fall-related hip fractures in older adults. J Am Geriatr Soc 2010 January;58(1):136-41. Inappropriate Outcomes
Ref ID: 180

(132) Fu FH, Guo LX, Zang YP. An overview of health fitness studies of Hong Kong residents from 2005 to 2011. J Exerc Sci Fit 2012;10(2):45-63. Inappropriate Study Design
Ref ID: 547

(133) Fulambarker A, Farooki B, Kheir F, Copur AS, Srinivasan L, Schultz S. Effect of yoga in chronic obstructive pulmonary disease. Am J Ther 2012 March;19(2):96-100. Inappropriate Study Design
Ref ID: 157

(134) Galantino ML, Bzdewka TM, Eissler-Russo JL, Holbrook ML, Mogck EP, Geigle P, Farrar JT. The impact of modified Hatha yoga on chronic low back pain: a pilot study. Altern Ther Health Med 2004 March;10(2):56-9. Inappropriate Study Design
Ref ID: 269

(135) Galantino ML, Shepard K, Krafft L, Laperriere A, Ducette J, Sorbello A, Barnish M, Condoluci D, Farrar JT. The effect of group aerobic exercise and tai chi on functional outcomes and quality of life for persons living with acquired immunodeficiency syndrome. J Altern Complement Med 2005 December;11(6):1085-92. Inappropriate Study Design
Ref ID: 250

(136) Galantino ML, Galbavy R, Quinn L. Therapeutic effects of yoga for children: a systematic review of the literature. Pediatr Phys Ther 2008;20(1):66-80. Inappropriate Population
Ref ID: 221

(137) Galantino ML, Greene L, Daniels L, Dooley B, Muscatello L, O'Donnell L. Longitudinal impact of yoga on chemotherapy-related cognitive impairment and quality of life in women with early stage breast cancer: A case series. Explore (NY) 2012;8(2):127-35. Inappropriate Study Design
Ref ID: 551

(138) Galantino ML, Greene L, Archetto B, Baumgartner M, Hassall P, Murphy JK, Umstetter J, Desai K. A qualitative exploration of the impact of yoga on breast cancer survivors with aromatase inhibitor-associated arthralgias. Explore (NY) 2012;8(1):40-7. Inappropriate Study Design
Ref ID: 552

(139) Galantino ML, Desai K, Greene L, Demichele A, Stricker CT, Mao JJ. Impact of yoga on functional outcomes in breast cancer survivors with aromatase inhibitor-associated arthralgias. Integr Cancer Ther 2012 December;11(4):313-20. Inappropriate Study Design
Ref ID: 130

(140) Galantino ML, Greene L, Daniels L, Dooley B, Muscatello L, O'Donnell L. Longitudinal impact of yoga on chemotherapy-related cognitive impairment and quality of life in women with early stage breast cancer: a case series. Explore (NY) 2012 March;8(2):127-35. Inappropriate Study Design
Ref ID: 103

(141) Galantino MLA. Blending traditional and alternative strategies for rehabilitation: Measuring functional outcomes and quality of life issues in an AIDS population. Ann Arbor: Temple University; 1997.

Inappropriate Study Design
Ref ID: 28

(142) Garrett M, Hogan N, Larkin A, Saunders J, Jakeman P, Coote S. Exercise in the community for people with minimal gait impairment due to MS: an assessor-blind randomized controlled trial. Mult Scler 2013 May;19(6):782-9. Inappropriate Study Design
Ref ID: 306

(143) Gaught AMH, Carneiro KA. Evidence for determining the exercise prescription in patients with osteoarthritis. Phys Sportsmed 2013;41(1):58-65. Inappropriate Study Design
Ref ID: 554

(144) Geyer R, Lyons A, Amazeen L, Alishio L, Cooks L. Feasibility study: the effect of therapeutic yoga on quality of life in children hospitalized with cancer. Pediatr Phys Ther 2011;23(4):375-9. Inappropriate Population
Ref ID: 112

(145) Gillespie LD, Robertson MC, Gillespie WJ, Lamb SE, Gates S, Cumming RG, Rowe BH. Interventions for preventing falls in older people living in the community. Cochrane Database Syst Rev 2009;(2). Inappropriate Outcomes
Ref ID: 556

(146) Gogulla S, Lemke N, Hauer K. [Effects of physical activity and physical training on the psychological status of older persons with and without cognitive impairment]. Z Gerontol Geriatr 2012 June;45(4):279-89. Inappropriate Study Design
Ref ID: 94

(147) Gorczynski P, Faulkner G. Exercise therapy for schizophrenia. Schizophr Bull 2010 July;36(4):665-6. Inappropriate Intervention
Ref ID: 169

(148) Gorczynski P, Faulkner G. Exercise therapy for schizophrenia. Cochrane Database Syst Rev 2010;(5):CD004412. Inappropriate Intervention
Ref ID: 171

(149) Granath J, Ingvarsson S, von TU, Lundberg U. Stress management: a randomized study of cognitive behavioural therapy and yoga. Cogn Behav Ther 2006;35(1):3-10. Inappropriate Study Design
Ref ID: 247

(150) Grazina R, Massano J. Physical exercise and Parkinson's disease: influence on symptoms, disease course and prevention. Rev Neurosci 2013;24(2):139-52. Inappropriate Study Design
Ref ID: 560

(151) Greendale GA, Huang MH, Karlamangla AS, Seeger L, Crawford S. Yoga decreases kyphosis in senior women and men with adult-onset hyperkyphosis: results of a randomized controlled trial. J Am Geriatr Soc 2009 September;57(9):1569-79. Inappropriate Study Design
Ref ID: 189

(152) Greenfield RH. A cool head: hypnosis and hot flashes. Altern Med Alert 2011 December;14(12):143-4. Inappropriate Study Design
Ref ID: 418

(153) Greenspan AI, Wolf SL, Kelley ME, O'Grady M. Tai chi and perceived health status in older adults who are transitionally frail: A randomized controlled trial. Phys Ther 2007;87(5):525-35. Inappropriate Study Design
Ref ID: 562

(154) Griebling TL. Re: Tai chi for lower urinary tract symptoms and quality of life in elderly patients with benign prostate hypertrophy: a randomized controlled trial. J Urol 2012 July;188(1):227. Inappropriate Study Design
Ref ID: 90

(155) Griffith JM, Hasley JP, Liu H, Severn DG, Conner LH, Adler LE. Qigong stress reduction in hospital staff. J Altern Complement Med 2008 October;14(8):939-45. Inappropriate Study Design
Ref ID: 209

(156) Groessl EJ, Weingart KR, Aschbacher K, Pada L, Baxi S. Yoga for veterans with chronic low-back pain. J Altern Complement Med 2008 November;14(9):1123-9. Inappropriate Study Design
Ref ID: 207

(157) Groessl EJ, Weingart KR, Johnson N, Baxi S. The benefits of yoga for women veterans with chronic low back pain. J Altern Complement Med 2012;18(9):832-8. Inappropriate Study Design
Ref ID: 565

(158) Gschwind YJ, Wolf I, Bridenbaugh SA, Kressig RW. Basis for a Swiss perspective on fall prevention in vulnerable older people. Swiss Med Wkly 2011;141. Inappropriate Study Design
Ref ID: 566

(159) Gusi N, Adsuar JC, Corzo H, del Pozo-Cruz B, Olivares PR, Parraca JA. Balance training reduces fear of falling and improves dynamic balance and isometric strength in institutionalised older people: A randomised trial. J Physiother 2012;58(2):97-104. Inappropriate Study Design
Ref ID: 567

(160) Haak T, Scott B. The effect of qigong on fibromyalgia (FMS): a controlled randomized study. Disabil Rehabil 2008;30(8):625-33. Inappropriate Study Design
Ref ID: 229

(161) Hall A, Maher C, Latimer J, Ferreira M. The effectiveness of tai chi for chronic musculoskeletal pain conditions: a systematic review and meta-analysis. Arthritis Rheum 2009 June 15;61(6):717-24. Inappropriate Study Design
Ref ID: 194

(162) Hall AM, Maher CG, Latimer J, Ferreira ML, Lam P. A randomized controlled trial of tai chi for long-term low back pain (TAI CHI): study rationale, design, and methods. BMC Musculoskelet Disord 2009;10:55. Inappropriate Study Design
Ref ID: 195

(163) Han A, Judd M, Welch V, Wu T, Tugwell P, Wells GA. Tai chi for treating rheumatoid arthritis. Cochrane Database Syst Rev 2004. Inappropriate Study Design
Ref ID: 3

(164) Harder H, Parlour L, Jenkins V. Randomised controlled trials of yoga interventions for women with breast cancer: a systematic literature review. Support Care Cancer 2012 December;20(12):3055-64. Inappropriate Study Design
Ref ID: 307

(165) Harinath K, Malhotra AS, Pal K, Prasad R, Kumar R, Kain TC, Rai L, Sawhney RC. Effects of Hatha yoga and Omkar meditation on cardiorespiratory performance, psychologic profile, and melatonin secretion. J Altern Complement Med 2004 April;10(2):261-8. Inappropriate Study Design
Ref ID: 264

(166) Hariprasad VR, Sivakumar PT, Koparde V, Varambally S, Thirthalli J, Varghese M, Basavaraddi IV, Gangadhar BN. Effects of yoga intervention on sleep and quality-of-life in elderly: A randomized controlled trial. Indian J Psychiatry 2013 July;55(Suppl 3):S364-S368. Inappropriate Study Design
Ref ID: 308

(167) Harris LR, Roberts L. Treatments for irritable bowel syndrome: patients' attitudes and acceptability. BMC Complement Altern Med 2008;8:65. Inappropriate Study Design
Ref ID: 206

(168) Hawker GA, Mian S, Bednis K, Stanaitis I. Osteoarthritis year 2010 in review: non-pharmacologic therapy. Osteoarthritis Cartilage 2011;19(4):366-74. Inappropriate Intervention
Ref ID: 571

(169) Heitkamp HC. Cardiac rehabilitation outpatient therapy - the current stage. Herz 1999;24(3):242-9. Inappropriate Outcomes
Ref ID: 572

(170) Heiwe S, Jacobson SH. Exercise training for adults with chronic kidney disease. Cochrane Database Syst Rev 2011;(10):CD003236. Inappropriate Study Design
Ref ID: 126

(171) Hill K, Smith R, Fearn M, Rydberg M, Oliphant R. Physical and psychological outcomes of a supported physical activity program for older carers. J Aging Phys Act 2007;15(3):257-71. Inappropriate Study Design
Ref ID: 574

(172) Hiller Scott A, Butin DN, Tewfik D, Burkhardt A, Mandel D, Nelson L. Occupational therapy as a means to wellness with the elderly. Phys Occup Ther Geriatr 2001;18(4):3-22. Inappropriate Study Design
Ref ID: 419

(173) Hillier SL, Hollohan V. Vestibular rehabilitation for unilateral peripheral vestibular dysfunction. Cochrane Database Syst Rev 2007;(4). Inappropriate Intervention
Ref ID: 575

(174) Hillier SL, McDonnell M. Vestibular rehabilitation for unilateral peripheral vestibular dysfunction. Cochrane Database Syst Rev 2011;(2). Inappropriate Intervention
Ref ID: 576

(175) Ho RTH, Yeung FSWA, Lo PHY, Law KY, Wong KOK, Cheung IKM, Ng SM. Tai-chi for residential patients with schizophrenia on movement coordination, negative symptoms, and functioning: A pilot randomized controlled trial. Evid Based Complement Alternat Med 2012. Inappropriate Study Design
Ref ID: 578

(176) Ho RTH, Chan JSM, Wang CW, Lau BWM, So KF, Yuen LP, Sham JST, Chan CLW. A randomized controlled trial of qigong exercise on fatigue symptoms, functioning, and telomerase activity in persons with chronic fatigue or chronic fatigue syndrome. Ann Behav Med 2012;44(2):160-70. Inappropriate Study Design
Ref ID: 577

(177) Ho TJ, Liang WM, Lien CH, Ma TC, Kuo HW, Chu BC, Chang HW, Lai JS, Lin JG. Health-related quality of life in the elderly practicing tai chi chuan. J Altern Complement Med 2007 December;13(10):1077-83. Inappropriate Study Design
Ref ID: 222

(178) Holland AE, Hill CJ, Jones AY, McDonald CF. Breathing exercises for chronic obstructive pulmonary disease. Cochrane Database Syst Rev 2012;10:CD008250. Inappropriate Study Design
Ref ID: 309

(179) Holloway EA, West RJ. Integrated breathing and relaxation training (the Papworth method) for adults with asthma in primary care: a randomised controlled trial. Thorax 2007;62(12):1039-42. Inappropriate Study Design
Ref ID: 580

(180) Howe TE, Rochester L, Neil F, Skelton DA, Ballinger C. Exercise for improving balance in older people. Cochrane Database Syst Rev 2011;(11):CD004963. Inappropriate Intervention
Ref ID: 114

(181) Huang TT, Yang LH, Liu CY. Reducing the fear of falling among community-dwelling elderly adults through cognitive-behavioural strategies and intense Tai Chi exercise: a randomized controlled trial. J Adv Nurs 2011 May;67(5):961-71. Inappropriate Study Design
Ref ID: 149

(182) Hubert M. [Physical therapy for Parkinson's disease]. Rev Med Brux 2011 September;32(4):388-92. Inappropriate Study Design
Ref ID: 119

(183) Hwa-Jin L, Hi-Joon P, Younbyoung C, Song-Yi K, Seung-Nam K, Seung-Tae K, Je-Ho K, Chang-Shik Y, Hyejung L. Tai chi qigong for the quality of life of patients with knee osteoarthritis: a pilot, randomized, waiting list controlled trial. Clin Rehabil 2009 June;23(6):504-11. Inappropriate Study Design
Ref ID: 420

(184) Hwang EY, Chung SY, Cho JH, Song MY, Kim S, Kim JW. Effects of a brief Qigong-based stress reduction program (BQSRP) in a distressed Korean population: a randomized trial. BMC Complement Altern Med 2013;13:113. Inappropriate Study Design
Ref ID: 310

(185) Ide MR, Laurindo IMM, Rodrigues AL, Tanaka C. Effect of aquatic respiratory exercise-based program in patients with fibromyalgia. Int J Rheum Dis 2008;11(2):131-40. Inappropriate Study Design
Ref ID: 585

(186) Ikai S, Uchida H, Suzuki T, Tsunoda K, Mimura M, Fujii Y. Effects of yoga therapy on postural stability in patients with schizophrenia-spectrum disorders: a single-blind randomized controlled trial. J Psychiatr Res 2013 November;47(11):1744-50. Inappropriate Study Design
Ref ID: 311

(187) Innes KE, Selfe TK, Taylor AG. Menopause, the metabolic syndrome, and mind-body therapies. Menopause 2008;15(5):1005-13. Inappropriate Study Design
Ref ID: 587

(188) Innes KE, Selfe TK, Vishnu A. Mind-body therapies for menopausal symptoms: A systematic review. Maturitas 2010;66(2):135-49. Inappropriate Study Design
Ref ID: 588

(189) Innes KE, Selfe TK. The effects of a gentle yoga program on sleep, mood, and blood pressure in older women with restless legs syndrome (rls): A preliminary randomized controlled trial. Evid Based Complement Alternat Med 2012. Inappropriate Study Design
Ref ID: 589

(190) Jacobs BP, Mehling W, Avins AL, Goldberg HA, Acree M, Lasater JH, Cole RJ, Riley DS, Maurer S. Feasibility of conducting a clinical trial on Hatha yoga for chronic low back pain: methodological lessons. Altern Ther Health Med 2004 March;10(2):80-3. Inappropriate Study Design
Ref ID: 267

(191) Jahnke R, Larkey L, Rogers C, Etnier J, Lin F. A comprehensive review of health benefits of qigong and tai chi. Am J Health Promot 2010 July;24(6):e1-e25. Inappropriate Study Design
Ref ID: 167

(192) Jain S, Mills PJ. Biofield Therapies: Helpful or Full of Hype? A Best Evidence Synthesis. Int J Behav Med 2010;17(1):1-16. Inappropriate Study Design
Ref ID: 590

(193) Janelsins MC, Davis PG, Wideman L, Katula JA, Sprod LK, Peppone LJ, Palesh OG, Heckler CE, Williams JP, Morrow GR, Mustian KM. effects of tai chi chuan on insulin and cytokine levels in a randomized controlled pilot study on breast cancer survivors. Clin Breast Cancer 2011 June;11(3):161-70. Inappropriate Study Design
Ref ID: 135

(194) Jellesma FC, Cornelis J. Mind magic: a pilot study of preventive mind-body-based stress reduction in behaviorally inhibited and activated children. J Holist Nurs 2012 March;30(1):55-62. Inappropriate Study Design
Ref ID: 120

(195) Jimenez-Martin PJs, Melendez-Ortega An, Albers U, Lopez-Diaz A. Beneficios del tai chi chuan en la osteoartritis, el equilibrio y la calidad de vida. / tai chi chuan benefits on osteoarthritis, balance and quality of life. RICYDE Rev Int Cienc Deporte 2013 April;9(32):181-99. Inappropriate Study Design
Ref ID: 421

(196) Jimenez PJ, Melendez A, Albers U. Psychological effects of tai chi chuan. Arch Gerontol Geriatr 2012;55(2):460-7. Inappropriate Study Design
Ref ID: 592

(197) Joshi S, Khandwe R, Bapat D, Deshmukh U. Effect of yoga on menopausal symptoms. Menopause Int 2011 September;17(3):78-81. Inappropriate Study Design
Ref ID: 128

(198) Jung S, Lee EN, Lee SR, Kim MS, Lee MS. Tai chi for lower urinary tract symptoms and quality of life in elderly patients with benign prostate hypertrophy: a randomized controlled trial. Evid Based Complement Alternat Med 2012;2012:624692. Inappropriate Study Design
Ref ID: 125

(199) Jyotsna VP, Joshi A, Ambekar S, Kumar N, Dhawan A, Sreenivas V. Comprehensive yogic breathing program improves quality of life in patients with diabetes. Indian J Endocrinol Metab 2012 May;16(3):423-8. Inappropriate Study Design
Ref ID: 93

(200) Kanaya AM, Araneta MR, Pawlowsky SB, Barrett-Connor E, Grady D, Vittinghoff E, Schembri M, Chang A, Carrion-Petersen ML, Coggins T, Tanori D, Armas JM, Cole RJ. Restorative yoga and metabolic risk factors: The Practicing Restorative Yoga vs. Stretching for the Metabolic Syndrome (PRYSMS) randomized trial. J Diabetes Complications 2013 December 10. Inappropriate Study Design
Ref ID: 313

(201) Kanitz JL, Pretzer K, Reif M, Witt K, Reulecke S, Voss A, Langler A, Henze G, Seifert G. The impact of eurythmy therapy on fatigue in healthy adults-A controlled trial. Eur J Integr Med 2012;4(3):E289-E297. Inappropriate Study Design
Ref ID: 595

(202) Kanitz JL, Camus MEM, Seifert G. Keeping the balance - an overview of mind-body therapies in pediatric oncology. Complement Ther Med 2013;21:S20-S25. Inappropriate Population
Ref ID: 596

(203) Karlsson MK, Magnusson H, von Schewelov T, Rosengren BE. Prevention of falls in the elderly-a review. Osteoporos Int 2013;24(3):747-62. Inappropriate Study Design
Ref ID: 598

(204) Karlsson MK, Vonschewelov T, Karlsson C, Coster M, Rosengen BE. Prevention of falls in the elderly: A review. Scand J Public Health 2013;41(5):442-54. Inappropriate Study Design
Ref ID: 597

(205) Kenne SE, Martensson LB, Holmberg SB, Andersson BA, Oden A, Bergh I. Mindfulness based stress reduction study design of a longitudinal randomized controlled complementary intervention in women with breast cancer. BMC Complement Altern Med 2013;13:248. Inappropriate Study Design
Ref ID: 314

(206) Kessel B, Kronenberg F. The role of complementary and alternative medicine in management of menopausal symptoms. Endocrinol Metab Clin North Am 2004;33(4):717-+. Inappropriate Outcomes
Ref ID: 599

(207) Khalsa SB, Shorter SM, Cope S, Wyshak G, Sklar E. Yoga ameliorates performance anxiety and mood disturbance in young professional musicians. Appl Psychophysiol Biofeedback 2009 December;34(4):279-89. Inappropriate Study Design
Ref ID: 190

(208) Kim HD, Je HD, Jeong JH, Ma SY. Effects of Tai Chi Training on Obstacle Avoidance and Gait Initiation by Older People. J Phys Ther Sci 2013;25(2):193-8. Inappropriate Study Design
Ref ID: 600

(209) Kim SD, Allen NE, Canning CG, Fung VSC. Postural instability in patients with parkinson's disease epidemiology, pathophysiology and management. CNS Drugs 2013;27(2):97-112. Inappropriate Outcomes
Ref ID: 601

(210) King MT, Bell ML, Costa D, Butow P, Oh B. The Quality of Life Questionnaire Core 30 (QLQ-C30) and Functional Assessment of Cancer-General (FACT-G) differ in responsiveness, relative efficiency, and therefore required sample size. J Clin Epidemiol 2014 January;67(1):100-7. Inappropriate Study Design
Ref ID: 315

(211) Kligler B, Homel P, Blank AE, Kenney J, Levenson H, Merrell W. Randomized trial of the effect of an integrative medicine approach to the management of asthma in adults on disease-related quality of life and pulmonary function. Altern Ther Health Med 2011 January;17(1):10-5. Inappropriate Study Design
Ref ID: 137

(212) Kligler B, McKee MD, Sackett E, Levenson H, Kenney J, Karasz A. An integrative medicine approach to asthma: who responds? J Altern Complement Med 2012 October;18(10):939-45. Inappropriate Study Design
Ref ID: 316

(213) Kohn M, Persson LU, Bryngelsson IL, Anderzen-Carlsson A, Westerdahl E. Medical yoga for patients with stress-related symptoms and diagnoses in primary health care: a randomized controlled trial. Evid Based Complement Alternat Med 2013;2013:215348. Inappropriate Study Design
Ref ID: 317

(214) Kovacic T, Kovacic M. Impact of relaxation training according to yoga in daily life (r) system on self-esteem after breast cancer surgery. J Altern Complement Med 2011;17(12):1157-64. Inappropriate Study Design
Ref ID: 606

(215) Kovacic T, Kovacic M. Impact of relaxation training according to yoga in daily life (r) system on perceived stress after breast cancer surgery. Integr Cancer Ther 2011;10(1):16-26. Inappropriate Study Design
Ref ID: 607

(216) Kozasa EH, Hachul H, Monson C, Pinto L, Garcia MC, Mello LEDM, Tufik S. Mind-body interventions for the treatment of insomnia: a review. Rev Bras Psiquiatr 2010;32(4):437-43. Inappropriate Outcomes
Ref ID: 608

(217) Krein SL, Kadri R, Hughes M, Kerr EA, Piette JD, Holleman R, Kim HM, Richardson CR. Pedometer-based internet-mediated intervention for adults with chronic low back pain: Randomized controlled trial. J Med Internet Res 2013;15(8). Inappropriate Study Design
Ref ID: 609

(218) Krisanaprakornkit T, Ngamjarus C, Witoonchart C, Piyavhatkul N. Meditation therapies for attention-deficit/hyperactivity disorder (ADHD). Cochrane Database Syst Rev 2010;(6). Inappropriate Study Design
Ref ID: 610

(219) Kronenberg F, Fugh-Berman A. Complementary and alternative medicine for menopausal symptoms: A review of randomized, controlled trials. Ann Intern Med2002;137(10):805-13. Inappropriate Outcomes
Ref ID: 611

(220) Kutner NG. How can exercise be incorporated into the routine care of patients on dialysis? Int Urol Nephrol 2007;39(4):1281-5. Inappropriate Study Design
Ref ID: 612

(221) Kvillemo P, Branstrom R. Experiences of a mindfulness-based stress-reduction intervention among patients with cancer. Cancer Nurs 2011 January;34(1):24-31. Inappropriate Study Design
Ref ID: 168

(222) Lan C, Chen SY, Lai JS, Wong AMK. Tai chi chuan in medicine and health promotion. Evid Based Complement Alternat Med 2013. Inappropriate Study Design
Ref ID: 614

(223) Lan C, Chen SY, Wong MK, Lai JS. Tai chi chuan exercise for patients with cardiovascular disease. Evid Based Complement Alternat Med 2013. Inappropriate Study Design
Ref ID: 613

(224) Langhorst J, Klose P, Dobos GJ, Bernardy K, Hauser W. Efficacy and safety of meditative movement therapies in fibromyalgia syndrome: a systematic review and meta-analysis of randomized controlled trials. Rheumatol Int 2013;33:193-207. Inappropriate Population
Ref ID: 20

(225) Lansinger B, Carlsson JY, Kreuter M, Taft C. Health-related quality of life in persons with long-term neck pain after treatment with qigong and exercise therapy respectively. European J Physiother 2013 September;15(3):111-7. Inappropriate Study Design
Ref ID: 422

(226) Lau HL, Kwong JS, Yeung F, Chau PH, Woo J. Yoga for secondary prevention of coronary heart disease. Cochrane Database Syst Rev 2012;12:CD009506. Inappropriate Study Design
Ref ID: 318

(227) Lauche R, Langhorst J, Dobos G, Cramer H. A systematic review and meta-analysis of Tai Chi for osteoarthritis of the knee. Complement Ther Med 2013 August;21(4):396-406. Inappropriate Outcomes
Ref ID: 320

(228) Lauche R, Cramer H, Hauser W, Dobos G, Langhorst J. A systematic review and meta-analysis of qigong for the fibromyalgia syndrome. Evid Based Complement Alternat Med 2013;2013:635182. Inappropriate Population
Ref ID: 319

(229) Lavretsky H, Alstein LL, Olmstead RE, Ercoli LM, Riparetti-Brown M, Cyr NS, Irwin MR. Complementary use of tai chi chih augments escitalopram treatment of geriatric depression: a randomized controlled trial. Am J Geriatr Psychiatry 2011 October;19(10):839-50. Inappropriate Study Design
Ref ID: 145

(230) Lazaridou A, Philbrook P, Tzika AA. Yoga and mindfulness as therapeutic interventions for stroke rehabilitation: a systematic review. Evid Based Complement Alternat Med 2013;2013:357108. Inappropriate Study Design
Ref ID: 321

(231) Lee HJ, Park HJ, Chae Y, Kim SY, Kim SN, Kim ST, Kim JH, Yin CS, Lee H. Tai chi qigong for the quality of life of patients with knee osteoarthritis: a pilot, randomized, waiting list controlled trial. Clin Rehabil 2009 June;23(6):504-11. Inappropriate Study Design
Ref ID: 196

(232) Lee HY, Hale CA, Hemingway B, Woolridge MW. Tai chi exercise and auricular acupressure for people with rheumatoid arthritis: an evaluation study. J Clin Nurs 2012 October;21(19-20):2812-22. Inappropriate Study Design
Ref ID: 84

(233) Lee LY, Lee DT, Woo J. Effect of tai chi on state self-esteem and health-related quality of life in older Chinese residential care home residents. J Clin Nurs 2007 August;16(8):1580-2. Inappropriate Study Design
Ref ID: 233

(234) Lee LYK, Lee DTF, Woo J. Tai chi and health-related quality of life in nursing home residents. J Nurs Sch 2009;41(1):35-43. Inappropriate Study Design
Ref ID: 624

(235) Lee LYK, Lee DTF, Woo J. The psychosocial effect of tai chi on nursing home residents. J Clin Nurs 2010;19(7-8):927-38. Inappropriate Study Design
Ref ID: 625

(236) Lee MS, Lee MS, Choi ES, Chung HT. Effects of qigong on blood pressure, blood pressure determinants and ventilatory function in middle-aged patients with essential hypertension. Am J Chin Med 2003;31(3):489-97. Inappropriate Study Design
Ref ID: 626

(237) Lee MS, Lee MS, Kim HJ, Choi ES. Effects of qigong on blood pressure, high-density lipoprotein cholesterol and other lipid levels in essential hypertension patients. Int J Neurosci 2004;114(7):777-86. Inappropriate Study Design
Ref ID: 627

(238) Lee MS, Pittler MH, Ernst E. Tai chi for rheumatoid arthritis: systematic review. Rheumatology (Oxford) 2007 November;46(11):1648-51. Inappropriate Study Design
Ref ID: 235

(239) Lee MS, Pittler MH, Ernst E. Is Tai chi an effective adjunct in cancer care? A systematic review of controlled clinical trials. Support Care Cancer 2007 June;15(6):597-601. Inappropriate Study Design
Ref ID: 322

(240) Lee MS, Chen KW, Sancier KM, Ernst E. Qigong for cancer treatment: a systematic review of controlled clinical trials. Acta Oncol 2007;46(6):717-22. Inappropriate Outcomes
Ref ID: 234

(241) Lee MS, Choi TY, Ernst E. Tai chi for breast cancer patients: a systematic review. Breast Cancer Res Treat 2010 April;120(2):309-16. Inappropriate Outcomes
Ref ID: 179

(242) Lee MS, Choi TY, Lim HJ, Ernst E. Tai chi for management of type 2 diabetes mellitus: a systematic review. Chin J Integr Med 2011 October;17(10):789-93. Inappropriate Study Design
Ref ID: 129

(243) Lee SW, Mancuso CA, Charlson ME. Prospective study of new participants in a community-based mind-body training program. J Gen Intern Med 2004 July;19(7):760-5. Inappropriate Study Design
Ref ID: 261

(244) Lee YK. Promoting psychosocial health of elderly residential care home residents: Implementation of a tai chi program. Ann Arbor: The Chinese University of Hong Kong (Hong Kong); 2006.

Inappropriate Study Design
Ref ID: 29

(245) Lengacher CA, Reich RR, Post-White J, Moscoso M, Shelton MM, Barta M, Le N, Budhrani P. Mindfulness based stress reduction in post-treatment breast cancer patients: an examination of symptoms and symptom clusters. J Behav Med 2012 February;35(1):86-94. Inappropriate Study Design
Ref ID: 141

(246) Leung RW, Alison JA, McKeough ZJ, Peters MJ. A study design to investigate the effect of short-form Sun-style tai chi in improving functional exercise capacity, physical performance, balance and health related quality of life in people with chronic obstructive pulmonary disease (COPD). Contemp Clin Trials 2011 March;32(2):267-72. Inappropriate Study Design
Ref ID: 156

(247) Leung RW, McKeough ZJ, Peters MJ, Alison JA. Short-form Sun-style tai chi as an exercise training modality in people with COPD. Eur Respir J 2013 May;41(5):1051-7. Inappropriate Study Design
Ref ID: 79

(248) Li AW, Goldsmith CA. The effects of yoga on anxiety and stress. Altern Med Rev 2012 March;17(1):21-35. Inappropriate Study Design
Ref ID: 96

(249) Li DX, Zhuang XY, Zhang YP, Guo H, Wang Z, Zhang Q, Feng YM, Yao YG. Effects of tai chi on the protracted abstinence syndrome: a time trial analysis. Am J Chin Med 2013;41(1):43-57. Inappropriate Study Design
Ref ID: 635

(250) Li F, Harmer P, McAuley E, Fisher KJ, Duncan TE, Duncan SC. Tai Chi, self-efficacy, and physical function in the elderly. Prev Sci 2001 December;2(4):229-39. Inappropriate Study Design
Ref ID: 276

(251) Li F, Harmer P, McAuley E, Duncan TE, Duncan SC, Chaumeton N, Fisher KJ. An evaluation of the effects of tai chi exercise on physical function among older persons: a randomized controlled trial. Ann Behav Med 2001;23(2):139-46. Inappropriate Study Design
Ref ID: 278

(252) Li F, Fisher KJ, Harmer P, McAuley E. Delineating the impact of tai chi training on physical function among the elderly. Am J Prev Med 2002 August;23(2 Suppl):92-7. Inappropriate Study Design
Ref ID: 274

(253) Li F, Fisher KJ, Harmer P, Irbe D, Tearse RG, Weimer C. Tai chi and self-rated quality of sleep and daytime sleepiness in older adults: a randomized controlled trial. J Am Geriatr Soc 2004 June;52(6):892-900. Inappropriate Study Design
Ref ID: 265

(254) Li F, Harmer P, Glasgow R, Mack KA, Sleet D, Fisher KJ, Kohn MA, Millet LM, Mead J, Xu J, Lin ML, Yang T, Sutton B, Tompkins Y. Translation of an effective tai chi intervention into a community-based falls-prevention program. Am J Public Health 2008;98(7):1195-8. Inappropriate Study Design
Ref ID: 384

(255) Li FZ, Harmer P, Chaumeton NR, Duncan TE, Duncan SC. Tai chi as a means to enhance self-esteem: A randomized controlled trial. J Appl Gerontol 2002;21(1):70-89. Inappropriate Study Design
Ref ID: 636

(256) Li FZ, Fisher KJ, Harmer P. Improving physical function and blood pressure in older adults through cobblestone mat walking: A randomized trial. J Am Geriatr Soc 2005;53(8):1305-12. Inappropriate Study Design
Ref ID: 637

(257) Li FZ, Harmer P, Fisher KJ, McAuley E, Chaumeton N, Eckstrom E, Wilson NL. Tai Chi and fall reductions in older adults: A randomized controlled trial. J Gerontol A Biol Sci Med Sci 2005;60(2):187-94. Inappropriate Study Design
Ref ID: 638

(258) Li F, Harmer P, McAuley E, Fisher KJ, Duncan TE, Duncan SC. Tai chi, self-efficacy, and physical function in the elderly. Prev Sci 2001 December;2(4):229-39. Inappropriate Study Design
Ref ID: 62

(259) Linder K, Svardsudd K. [Qigong has a relieving effect on stress]. Lakartidningen 2006 June 14;103(24-25):1942-5. Inappropriate Study Design
Ref ID: 244

(260) Littman AJ, Bertram LC, Ceballos R, Ulrich CM, Ramaprasad J, McGregor B, McTiernan A. Randomized controlled pilot trial of yoga in overweight and obese breast cancer survivors: effects on quality of life and anthropometric measures. Support Care Cancer 2012 February;20(2):267-77. Inappropriate Study Design
Ref ID: 150

(261) Liu J, Li BQ, Shnider R. Effects of tai chi training on improving physical function in patients with coronary heart diseases. J Exerc Sci Fit 2010;8(2):78-84. Inappropriate Study Design
Ref ID: 642

(262) Liu X, Miller YD, Burton NW, Chang JH, Brown WJ. The effect of tai chi on health-related quality of life in people with elevated blood glucose or diabetes: a randomized controlled trial. Qual Life Res 2013 September;22(7):1783-6. Inappropriate Study Design
Ref ID: 323

(263) Liu XD, Jin HZ, Ng BHP, Gu YH, Wu YC, Lu G. Therapeutic effects of qigong in patients with COPD: a randomized controlled trial. Hong Kong J Occup Ther 2012;22(1):38-46. Inappropriate Study Design
Ref ID: 644

(264) Liu X, Miller YD, Burton NW, Chang JH, Brown WJ. The effect of tai chi on health-related quality of life in people with elevated blood glucose or diabetes: A randomized controlled trial. Qual Life Res 2013 September;22(7):1783-6. Inappropriate Study Design
Ref ID: 33

(265) Logghe IHJ, Zeeuwe PEM, Verhagen AP, Wijnen-Sponselee RMT, Willemsen SP, Bierma-Zeinstra SMA, Van Rossum E, Faber MJ, Koes BW. Lack of effect of tai chi chuan in preventing falls in elderly people living at home: A randomized clinical trial. J Am Geriatr Soc 2009 January;57(1):70-5. Inappropriate Study Design
Ref ID: 63

(266) Logghe IHJ, Verhagen AP, Rademaker ACHJ, Bierma-Zeinstra SMA, Van Rossum E, Faber MJ, Koes BW. The effects of tai chi on fall prevention, fear of falling and balance in older people: A meta-analysis. Prev Med 2010 September;51(3-4):222-7. Inappropriate Outcomes
Ref ID: 37

(267) Logghe IHJ, Verhagen AP, Rademaker ACHJ, Zeeuwe PEM, Bierma-Zeinstra SMA, Van Rossum E, Faber MJ, Van Haastregt JCM, Koes BW. Explaining the ineffectiveness of a tai chi fall prevention training for community-living older people: A process evaluation alongside a randomized clinical trial (RCT). Arch Gerontol Geriatr 2011 May;52(3):357-62. Inappropriate Study Design
Ref ID: 64

(268) Loh SY, Lee SY, Quek KF, Murray L. Barriers to participation in a randomized controlled trial of qigong exercises amongst cancer survivors: Lessons learnt. Asian Pac J Cancer Prev 2012;13(12):6337-42. Inappropriate Study Design
Ref ID: 645

(269) Long AR, Rouster-Stevens KA. The role of exercise therapy in the management of juvenile idiopathic arthritis. Curr Opin Rheumatol 2010;22(2):213-7. Inappropriate Population
Ref ID: 646

(270) Loudon A, Barnett T, Piller N, Immink MA, Visentin D, Williams AD. The effect of yoga on women with secondary arm lymphoedema from breast cancer treatment. BMC Complement Altern Med 2012;12:66. Inappropriate Study Design
Ref ID: 92

(271) Lundgren T, Dahl J, Yardi N, Melin L. Acceptance and commitment therapy and yoga for drug-refractory epilepsy: a randomized controlled trial. Epilepsy Behav 2008 July;13(1):102-8. Inappropriate Study Design
Ref ID: 220

(272) Luskin F. Transformative practices for integrating mind-body-spirit. J Altern Complement Med 2004;10 Suppl 1:S15-S23. Inappropriate Study Design
Ref ID: 258

(273) Mailhan L, Papeix C. [Non-medicinal treatments of spasticity in multiple sclerosis]. Rev Neurol (Paris) 2012 April;168 Suppl 3:S57-S61. Inappropriate Study Design
Ref ID: 88

(274) Malchow B, Reich-Erkelenz D, Oertel-Knochel V, Keller K, Hasan A, Schmitt A, Scheewe TW, Cahn W, Kahn RS, Falkai P. The effects of physical exercise in schizophrenia and affective disorders. Eur Arch Psychiatry Clin Neurosci 2013;263(6):451-67. Inappropriate Study Design
Ref ID: 649

(275) Mamtani R, Mamtani R. Ayurveda and yoga in cardiovascular diseases. Cardiol Rev 2004 September;12(5):155-62. Inappropriate Study Design
Ref ID: 257

(276) Mann E, Smith M, Hellier J, Hunter MS. A randomised controlled trial of a cognitive behavioural intervention for women who have menopausal symptoms following breast cancer treatment (MENOS 1): Trial protocol. BMC Cancer 2011;11. Inappropriate Study Design
Ref ID: 650

(277) Manocha R, Semmar B, Black D. A pilot study of a mental silence form of meditation for women in perimenopause. J Clin Psychol Med Settings 2007;14(3):266-73. Inappropriate Study Design
Ref ID: 652

(278) Markham AW, Wilkinson JM. Complementary and Alternative Medicines (CAM) in the management of asthma: An examination of the evidence. J Asthma 2004;41(2):131-9. Inappropriate Study Design
Ref ID: 654

(279) Martinez-Devesa P, Perera R, Theodoulou M, Waddell A. Cognitive behavioural therapy for tinnitus. Cochrane Database Syst Rev 2010;(9):CD005233. Inappropriate Intervention
Ref ID: 162

(280) Martinez DP, Waddell A, Perera R, Theodoulou M. Cognitive behavioural therapy for tinnitus. Cochrane Database Syst Rev 2007;(1):CD005233. Inappropriate Intervention
Ref ID: 241

(281) McCain NL, Gray DP, Elswick RK, Robins JW, Tuck I, Walter JA, Rausch SM, Ketchum JM. A randomized clinical trial of alternative stress management interventions in persons with HIV infection. J Consult Clin Psychol 2008;76(3):431-41. Inappropriate Study Design
Ref ID: 656

(282) McDonnell MN, Smith AE, Mackintosh SF. Aerobic exercise to improve cognitive function in adults with neurological disorders: A systematic review. Arch Phys Med Rehabil2011;92(7):1044-52. Inappropriate Intervention
Ref ID: 657

(283) Mckee KE, Hackney ME. The effects of adapted tango on spatial cognition and disease severity in Parkinson's Disease. J Mot Behav 2013;45(6):519-29. Inappropriate Study Design
Ref ID: 658

(284) McMahon S, Fleury J. External validity of physical activity interventions for community-dwelling older adults with fall risk: a quantitative systematic literature review. J Adv Nurs 2012;68(10):2140-54. Inappropriate Study Design
Ref ID: 659

(285) Merom D, Cumming R, Mathieu E, Anstey KJ, Rissel C, Simpson JM, Morton RL, Cerin E, Sherrington C, Lord SR. Can social dancing prevent falls in older adults? a protocol of the Dance, Aging, Cognition, Economics (DAnCE) fall prevention randomised controlled trial. BMC Public Health 2013;13. Inappropriate Study Design
Ref ID: 660

(286) Michalsen A, Jeitler M, Brunnhuber S, Ludtke R, Bussing A, Musial F, Dobos G, Kessler C. Iyengar yoga for distressed women: a 3-armed randomized controlled trial. Evid Based Complement Alternat Med 2012;2012:408727. Inappropriate Study Design
Ref ID: 325

(287) Michalsen A, Traitteur H, Ludtke R, Brunnhuber S, Meier L, Jeitler M, Bussing A, Kessler C. Yoga for chronic neck pain: a pilot randomized controlled clinical trial. J Pain 2012 November;13(11):1122-30. Inappropriate Study Design
Ref ID: 324

(288) Middleton KR, Ward MM, Haaz S, Velummylum S, Fike A, Acevedo AT, Tataw-Ayuketah G, Dietz L, Mittleman BB, Wallen GR. A pilot study of yoga as self-care for arthritis in minority communities. Health Qual Life Outcomes 2013;11:55. Inappropriate Study Design
Ref ID: 326

(289) Miller RG. Fatigue and therapeutic exercise. J Neurol Sci 2006;242(1-2):37-41. Inappropriate Outcomes
Ref ID: 663

(290) Ming-Chien C, James CR, Sawyer SF, Brismee JM, Xu KT, Poklikuha G, Dunn DM, Chwan-Li S. Effects of tai chi exercise on posturography, gait, physical function and quality of life in postmenopausal women with osteopaenia: a randomized clinical study. Clin Rehabil 2010 December 1;24(12):1080-90. Inappropriate Study Design
Ref ID: 423

(291) Mishra SI, Scherer RW, Snyder C, Geigle PM, Berlanstein DR, Topaloglu O. Exercise interventions on health-related quality of life for people with cancer during active treatment. Cochrane Database Syst Rev 2012;8:CD008465. Inappropriate Intervention
Ref ID: 327

(292) Mishra SI, Scherer RW, Geigle PM, Berlanstein DR, Topaloglu O, Gotay CC, Snyder C. Exercise interventions on health-related quality of life for cancer survivors. Cochrane Database Syst Rev 2012;(8). Inappropriate Study Design
Ref ID: 665

(293) Moadel AB, Shah C, Wylie-Rosett J, Harris MS, Patel SR, Hall CB, Sparano JA. Randomized controlled trial of yoga among a multiethnic sample of breast cancer patients: effects on quality of life. J Clin Oncol 2007 October 1;25(28):4387-95. Inappropriate Study Design
Ref ID: 230

(294) Monti DA, Sufian M, Peterson C. Potential role of mind-body therapies in cancer Survivorship. Cancer 2008;112(11):2607-16. Inappropriate Study Design
Ref ID: 667

(295) Morone NE, Greco CM. Mind-body interventions for chronic pain in older adults: A structured review. Pain Med 2007;8(4):359-75. Inappropriate Study Design
Ref ID: 668

(296) Morrow PK, Mattair DN, Hortobagyi GN. Hot flashes: a review of pathophysiology and treatment modalities. Oncologist 2011;16(11):1658-64. Inappropriate Study Design
Ref ID: 118

(297) Munshi A, Ni LH, Tiwana MS. Complementary and alternative medicine in present day oncology care: Promises and pitfalls. Jpn J Clin Oncol 2008;38(8):512-20. Inappropriate Study Design
Ref ID: 670

(298) Murthy V, Chamberlain RS. Menopausal symptoms in young survivors of breast cancer: a growing problem without an ideal solution. Cancer Control 2012;19(4):317-29. Inappropriate Outcomes
Ref ID: 671

(299) Mustian KM, Katula JA, Gill DL, Roscoe JA, Lang D, Murphy K. Tai chi chuan, health-related quality of life and self-esteem: a randomized trial with breast cancer survivors. Support Care Cancer 2004 December;12(12):871-6. Inappropriate Study Design
Ref ID: 259

(300) Mustian KM, Palesh OG, Flecksteiner SA. Tai chi chuan for breast cancer survivors. Med Sport Sci 2008;52:209-17. Inappropriate Study Design
Ref ID: 218

(301) Mustian KM, Sprod LK, Palesh OG, Peppone LJ, Janelsins MC, Mohile SG, Carroll J. Exercise for the management of side effects and quality of life among cancer survivors. Curr Sports Med Rep 2009;8(6):325-30. Inappropriate Study Design
Ref ID: 673

(302) Mustian KM, Sprod LK, Janelsins M, Peppone LJ, Palesh OG, Chandwani K, Reddy PS, Melnik MK, Heckler C, Morrow GR. Multicenter, randomized controlled trial of yoga for sleep quality among cancer survivors. J Clin Oncol 2013 September 10;31(26):3233-41. Inappropriate Study Design
Ref ID: 328

(303) Narahari SR, Bose KS, Aggithaya MG, Swamy GK, Ryan TJ, Unnikrishnan B, Washington RG, Rao BP, Rajagopala S, Manjula K, Vandana U, Sreemol TA, Rojith M, Salimani SY, Shefuvan M. Community level morbidity control of lymphoedema using self care and integrative treatment in two lymphatic filariasis endemic districts of South India: a non-randomized interventional study. Trans R Soc Trop Med Hyg 2013 September;107(9):566-77. Inappropriate Study Design
Ref ID: 329

(304) Newshan G, Staats JA. Evidence-based pain guidelines in hiv care. J Assoc Nurses AIDS Care 2013;24(1):S112-S126. Inappropriate Outcomes
Ref ID: 675

(305) Ng BH, Tsang HW, Jones AY, So CT, Mok TY. Functional and psychosocial effects of health qigong in patients with COPD: a randomized controlled trial. J Altern Complement Med 2011 March;17(3):243-51. Inappropriate Study Design
Ref ID: 144

(306) Ng HPB. Effect of qigong on physical and psychosocial status of Chinese COPD patients: A randomized controlled trial. Ann Arbor: Hong Kong Polytechnic University (Hong Kong); 2010.Inappropriate Study Design
Ref ID: 30

(307) Ng SM, Wang CW, Ho RT, Ziea TC, He J, Wong VC, Chan CL. Tai chi exercise for patients with heart disease: a systematic review of controlled clinical trials. Altern Ther Health Med 2012 May;18(3):16-22. Inappropriate Study Design
Ref ID: 81

(308) Nguyen HT, Grzywacz JG, Lang W, Walkup M, Arcury TA. effects of complementary therapy on health in a national us sample of older adults. J Altern Complement Med 2010;16(7):701-6. Inappropriate Study Design
Ref ID: 677

(309) Nidhi R, Padmalatha V, Nagarathna R, Amritanshu R. Effect of yoga program on quality of life in adolescent Polycystic Ovarian Syndrome: A randomized control trial. Appl Res Qual Life 2013 September;8(3):373-83. Inappropriate Study Design
Ref ID: 67

(310) Norris JM, Culos-Reed SN, Carlson LE, Aldous SH. Utilizing the TPB for understanding yoga participation in cancer survivors. J Sport Exerc Psychol 2007 July 2;29:S194. Inappropriate Study Design
Ref ID: 424

(311) Norweg A, Collins EG. Evidence for cognitive-behavioral strategies improving dyspnea and related distress in COPD. Int J Chron Obstruct Pulmon Dis 2013;8:439-51. Inappropriate Intervention
Ref ID: 330

(312) Nyer M, Doorley J, Durham K, Yeung AS, Freeman MP, Mischoulon D. What is the role of alternative treatments in late-life depression? Psychiatr Clin North Am 2013;36(4):577. Inappropriate Outcomes
Ref ID: 680

(313) O'Connor E, Patnode CD, Burda BU, Buckley DI, Whitlock EP. unknown 2012 September. Inappropriate Outcomes
Ref ID: 331

(314) O'Math+¦na DnP. Non-specific factors come to the fore in study of biofield therapy. Altern Med Alert 2011 October;14(10):117-8. Inappropriate Study Design
Ref ID: 425

(315) Oh B, Butow P, Mullan B, Clarke S. Medical qigong for cancer patients: pilot study of impact on quality of life, side effects of treatment and inflammation. Am J Chin Med 2008;36(3):459-72. Inappropriate Study Design
Ref ID: 215

(316) Oh B, Butow P, Mullan B, Clarke S, Beale P, Pavlakis N, Kothe E, Lam L, Rosenthal D. Impact of medical qigong on quality of life, fatigue, mood and inflammation in cancer patients: a randomized controlled trial. Ann Oncol 2010 March;21(3):608-14. Inappropriate Study Design
Ref ID: 186

(317) Oh B, Butow P, Mullan B, Hale A, Lee MS, Guo X, Clarke S. A critical review of the effects of medical qigong on quality of life, immune function, and survival in cancer patients. Integr Cancer Ther 2012 June;11(2):101-10. Inappropriate Study Design
Ref ID: 132

(318) Oh B, Butow PN, Mullan BA, Clarke SJ, Beale PJ, Pavlakis N, Lee MS, Rosenthal DS, Larkey L, Vardy J. Effect of medical qigong on cognitive function, quality of life, and a biomarker of inflammation in cancer patients: a randomized controlled trial. Support Care Cancer 2012 June;20(6):1235-42. Inappropriate Study Design
Ref ID: 134

(319) Oh B, Choi S, Inamori A, Rosenthal D, Yeung A. Effects of qigong on depression: a systemic review. Evid Based Complement Alternat Med 2013. Inappropriate Study Design
Ref ID: 685

(320) Oken BS, Zajdel D, Kishiyama S, Flegal K, Dehen C, Haas M, Kraemer DF, Lawrence J, Leyva J. Randomized, controlled, six-month trial of yoga in healthy seniors: effects on cognition and quality of life. Altern Ther Health Med 2006 January;12(1):40-7. Inappropriate Study Design
Ref ID: 249

(321) Oken BS, Fonareva I, Haas M, Wahbeh H, Lane JB, Zajdel D, Amen A. Pilot controlled trial of mindfulness meditation and education for dementia caregivers. J Altern Complement Med 2010;16(10):1031-8. Inappropriate Study Design
Ref ID: 688

(322) Ospina MB, Bond K, Karkhaneh M, Buscemi N, Dryden DM, Barnes V, Carlson LE, Dusek JA, Shannahoff-Khalsa D. Clinical trials of meditation practices in health care: characteristics and quality. J Altern Complement Med 2008 December;14(10):1199-213. Inappropriate Study Design
Ref ID: 205

(323) Overcash J, Will KM, Lipetz DW. The benefits of medical qigong in patients with cancer: A descriptive pilot study. Clin J Oncol Nurs 2013;17(6):654-8. Inappropriate Study Design
Ref ID: 689

(324) Page MJ, O'Connor D, Pitt V, Massy-Westropp N. Exercise and mobilisation interventions for carpal tunnel syndrome. Cochrane Database Syst Rev 2012;6:CD009899. Inappropriate Outcomes
Ref ID: 89

(325) Park IS, Song R, Oh KO, So HY, Kim DS, Kim JI, Kim TS, Kim HL, Ahn SH. Managing cardiovascular risks with tai chi in people with coronary artery disease. J Adv Nurs 2010;66(2):282-92. Inappropriate Study Design
Ref ID: 692

(326) Park J, McCaffrey R, Dunn D, Goodman R. Managing osteoarthritis: comparisons of chair yoga, Reiki, and education (pilot study). Holist Nurs Pract 2011 November;25(6):316-26. Inappropriate Study Design
Ref ID: 124

(327) Park JE, Liu Y, Park T, Hong S, Kim JE, Kim TH, Kim AR, Jung SY, Park H, Choi SM. A trial for the use of qigong in the treatment of pre and mild essential hypertension: a study protocol for a randomized controlled trial. Trials 2011;12:244. Inappropriate Study Design
Ref ID: 111

(328) Patel NK, Newstead AH, Ferrer RL. The effects of yoga on physical functioning and health related quality of life in older adults: a systematic review and meta-analysis. J Altern Complement Med 2012 October;18(10):902-17. Inappropriate Comparison Group
Ref ID: 333

(329) Patel SR. The effects of yoga on mood disturbance and pain in an underserved breast cancer population. Ann Arbor: Yeshiva University; 2004.

Inappropriate Study Design
Ref ID: 31

(330) Patra S, Telles S. Positive impact of cyclic meditation on subsequent sleep. Med Sci Monit 2009;15(7):CR375-CR381. Inappropriate Study Design
Ref ID: 694

(331) Paul A, Cramer H, Lauche R, Altner N, Langhorst J, Dobos GJ. An oncology mind-body medicine day care clinic: concept and case presentation. Integr Cancer Ther 2013;12(6):503-7. Inappropriate Study Design
Ref ID: 695

(332) Peng PWH. Tai chi and chronic pain. Reg Anesth Pain Med 2012;37(4):372-82. Inappropriate Study Design
Ref ID: 696

(333) Pizzigalli L, Filippini A, Ahmaidi S, Jullien H, Rainoldi A. Prevention of falling risk in elderly rpople: the relevance of muscular strength and symmetry of lower limbs in postural stability. J Strength Cond Res 2011;25(2):567-74. Inappropriate Outcomes
Ref ID: 697

(334) Pluchino A, Lee SY, Asfour S, Roos BA, Signorile JF. Pilot study comparing changes in postural control after training using a video game balance board program and 2 standard activity-based balance intervention programs. Arch Phys Med Rehabil 2012;93(7):1138-46. Inappropriate Study Design
Ref ID: 698

(335) Pokladnikova J, Selke-Krulichova I. Effectiveness of a comprehensive lifestyle modification program for asthma patients: a randomized controlled pilot trial. J Asthma 2013;50(3):318-26. Inappropriate Study Design
Ref ID: 699

(336) Posadzki P, Ernst E. Yoga for asthma? A systematic review of randomized clinical trials. J Asthma 2011 August;48(6):632-9. Inappropriate Outcomes
Ref ID: 136

(337) Prem V, Sahoo RC, Adhikari P. Comparison of the effects of Buteyko and pranayama breathing techniques on quality of life in patients with asthma - a randomized controlled trial. Clin Rehabil 2013;27(2):133-41. Inappropriate Study Design
Ref ID: 700

(338) Pullen PR, Nagamia SH, Mehta PK, Thompson WR, Benardot D, Hammoud R, Parrott JM, Sola S, Khan BV. Effects of yoga on inflammation and exercise capacity in patients with chronic heart failure. J Card Fail 2008 June;14(5):407-13. Inappropriate Study Design
Ref ID: 216

(339) Pullen PR, Thompson WR, Benardot D, Brandon LJ, Mehta PK, Rifai L, Vadnais DS, Parrott JM, Khan BV. Benefits of yoga for African American heart failure patients. Med Sci Sports Exerc 2010 April;42(4):651-7. Inappropriate Study Design
Ref ID: 183

(340) Quick M, Kiefer D. Qigong reduces depression in women with breast cancer receiving radiotherapy. Integr Med Alert 2013 May;16(5):54-7. Inappropriate Study Design
Ref ID: 427

(341) Qureshi NA, Al-Bedah AM. Mood disorders and complementary and alternative medicine: a literature review. Neuropsychiatr Dis Treat 2013;9:639-58. Inappropriate Study Design
Ref ID: 703

(342) Rabadi MH. Review of the randomized clinical stroke rehabilitation trials in 2009. Med Sci Monit2011;17(2):RA25-RA43. Inappropriate Outcomes
Ref ID: 704

(343) Rae M. Yoga philosophy - Vikalpa and Kalpana imagery for health. Active Living: Newsletters 2009 November;23-4. Inappropriate Study Design
Ref ID: 428

(344) Raghavendra RM, Nagarathna R, Nagendra HR, Gopinath KS, Srinath BS, Ravi BD, Patil S, Ramesh BS, Nalini R. Effects of an integrated yoga programme on chemotherapy-induced nausea and emesis in breast cancer patients. Eur J Cancer Care (Engl) 2007 November;16(6):462-74. Inappropriate Study Design
Ref ID: 228

(345) Raghavendra RM, Vadiraja HS, Nagarathna R, Nagendra HR, Rekha M, Vanitha N, Gopinath KS, Srinath BS, Vishweshwara MS, Madhavi YS, cortisol rhythm and mood states in early breast cancer patients undergoing adjuvant radiotherapy: a randomized controlled trial. Integr Cancer Ther 2009;8(1):37-46. Inappropriate Study Design
Ref ID: 706

(346) Raja-Khan N, Stener-Victorin E, Wu XK, Legro RS. The physiological basis of complementary and alternative medicines for polycystic ovary syndrome. Am J Physiol Endocrinol Metab 2011;301(1):E1-E10. Inappropriate Outcomes
Ref ID: 707

(347) Rakhshani A, Maharana S, Raghuram N, Nagendra HR, Venkatram P. Effects of integrated yoga on quality of life and interpersonal relationship of pregnant women. Qual Life Res 2010 December;19(10):1447-55. Inappropriate Study Design
Ref ID: 166

(348) Ram A, Raghuram N, Rao RM, Bhargav H, Koka PS, Tripathi S, Nelamangala RV, Kodaganur GS, Ramarao NH. Development and validation of a need-based integrated yoga program for cancer patients: a retrospective study. J Stem Cells 2012;7(4):269-82. Inappropriate Study Design
Ref ID: 107

(349) Ramachandran AK, Rosengren KS, Yang Y, Hsiao-Wecksler ET. Effect of tai chi on gait and obstacle crossing behaviors in middle-aged adults. Gait Posture 2007;26(2):248-55. Inappropriate Study Design
Ref ID: 709

(350) Ramaratnam S, Baker GA, Goldstein LH. Psychological treatments for epilepsy. Cochrane Database Syst Rev 2008. Inappropriate Intervention
Ref ID: 12

(351) Rand D, Miller WC, Yiu J, Eng JJ. Interventions for addressing low balance confidence in older adults: a systematic review and meta-analysis. Age Ageing 2011 May;40(3):297-306. Inappropriate Outcomes
Ref ID: 140

(352) Ransmayr G. Physical, occupational, speech and swallowing therapies and physical exercise in Parkinson's disease. J Neural Transm 2011;118(5):773-81. Inappropriate Study Design
Ref ID: 710

(353) Rao MR, Raghuram N, Nagendra HR, Gopinath KS, Srinath BS, Diwakar RB, Patil S, Bilimagga SR, Rao N, Varambally S. Anxiolytic effects of a yoga program in early breast cancer patients undergoing conventional treatment: A randomized controlled trial. Complement Ther Med 2009;17(1):1-8. Inappropriate Study Design
Ref ID: 711

(354) Rao RM, Nagendra HR, Raghuram N, Vinay C, Chandrashekara S, Gopinath KS, Srinath BS. Influence of yoga on mood states, distress, quality of life and immune outcomes in early stage breast cancer patients undergoing surgery. Int J Yoga 2008 January;1(1):11-20. Inappropriate Study Design
Ref ID: 223

(355) Rausch SM. Evaluating the psychosocial effects of two interventions, tai chi and spiritual growth groups, in women with breast cancer. US: ProQuest Information & Learning; 2008. Inappropriate Study Design
Ref ID: 69

(356) Reed SD, Guthrie KA, Newton KM, Anderson GL, Booth-Laforce C, Caan B, Carpenter JS, Cohen LS, Dunn AL, Ensrud KE, Freeman EW, Hunt JR, Joffe H, Larson JC, Learman LA, Rothenberg R, Seguin RA, Sherman KJ, Sternfeld BS, Lacroix AZ. Menopausal quality of life: RCT of yoga, exercise, and omega-3 supplements. Am J Obstet Gynecol 2014 March;210(3):244. Inappropriate Study Design
Ref ID: 334

(357) Reimers N, Reimers CD. Exercise for lower back pain, hip and knee osteoarthritis, and fibromyalgia: effects on pain - A literature review. Aktuelle Rheumatol 2012;37(3):174-88. Inappropriate Study Design; Ref ID: 712

(358) Ren H, Collins V, Clarke SJ, Han JS, Lam P, Clay F, Williamson L, Choo KHA. Epigenetic changes in response to tai chi practice: A pilot investigation of DNA methylation marks. Evid Based Complement Alternat Med 2012. Inappropriate Study Design; Ref ID: 713

(359) Rendant D, Pach D, Ludtke R, Reisshauer A, Mietzner A, Willich SN, Witt CM. Qigong versus exercise versus no therapy for patients with chronic neck pain: a randomized controlled trial. Spine (Phila Pa 1976) 2011 March 15;36(6):419-27. Inappropriate Study Design; Ref ID: 152

(360) Rinaldi S, Fontani V, Aravagli L, Margotti ML. Psychological and symptomatic stress-related disorders with radio-electric treatment: psychometric evaluation. Stress Health 2010;26(5):350-8. Inappropriate Study Design
Ref ID: 715

(361) Ringdahl E, Pandit S. Treatment of knee osteoarthritis. Am Fam Physician 2011;83(11):1287-92. Inappropriate Study Design; Ref ID: 716

(362) Roa W, Brasher PM, Bauman G, Anthes M, Bruera E, Chan A, Fisher B, Fulton D, Gulavita S, Hao C, Husain S, Murtha A, Petruk K, Stewart D, Tai P, Urtasun R, Cairncross JG, Forsyth P. Abbreviated course of radiation therapy in older patients with glioblastoma multiforme: a prospective randomized clinical trial. J Clin Oncol 2004 May 1;22(9):1583-8. Inappropriate Study Design; Ref ID: 270

(363) Robins JL, McCain NL, Gray DP, Elswick RK, Jr., Walter JM, McDade E. Research on psychoneuroimmunology: tai chi as a stress management approach for individuals with HIV disease. Appl Nurs Res 2006 February;19(1):2-9. Inappropriate Study Design
Ref ID: 248

(364) Robins JL, Elswick RK, McCain NL. The story of the evolution of a unique tai chi form: origins, philosophy, and research. J Holist Nurs 2012 September;30(3):134-46. Inappropriate Study Design; Ref ID: 106

(365) Robins JL, McCain NL, Elswick RK, Jr., Walter JM, Gray DP, Tuck I. Psychoneuroimmunology-Based Stress Management during Adjuvant Chemotherapy for Early Breast Cancer. Evid Based Complement Alternat Med 2013;2013:372908. Inappropriate Study Design; Ref ID: 335

(366) Rogers C, Keller C, Larkey LK. Perceived benefits of meditative movement in older adults. Geriatr Nurs 2010;31(1):37-51. Inappropriate Study Design
Ref ID: 718

(367) Roland KP, Jakobi JM, Powell C, Jones GR. Factors related to functional independence in females with Parkinson's disease: A systematic review. Maturitas 2011;69(4):304-11. Inappropriate Intervention
Ref ID: 393

(368) Ross A, Friedmann E, Bevans M, Thomas S. National survey of yoga practitioners: mental and physical health benefits. Complement Ther Med 2013 August;21(4):313-23. Inappropriate Study Design
Ref ID: 336

(369) Rubenstein LZ, Josephson KR. Falls and their prevention in elderly people: What does the evidence show? Med Clin North Am 2006;90(5):807. Inappropriate Study Design
Ref ID: 719

(370) Sabina AB, Williams AL, Wall HK, Bansal S, Chupp G, Katz DL. Yoga intervention for adults with mild-to-moderate asthma: a pilot study. Ann Allergy Asthma Immunol 2005 May;94(5):543-8. Inappropriate Study Design
Ref ID: 254

(371) Sadja J, Mills PJ. Effects of yoga interventions on fatigue in cancer patients and survivors: A systematic review of randomized controlled trials. Explore (NY)2013;9(4):232-43. Inappropriate Outcomes
Ref ID: 721

(372) Sakuma Y, Sasaki-Otomaru A, Ishida S, Kanoya Y, Arakawa C, Mochizuki Y, Seiishi Y, Sato C. Effect of a home-based simple yoga program in child-care workers: a randomized controlled trial. J Altern Complement Med 2012;18(8):769-76. Inappropriate Study Design
Ref ID: 722

(373) Santaella DF, Devesa CR, Rojo MR, Amato MB, Drager LF, Casali KR, Montano N, Lorenzi-Filho G. Yoga respiratory training improves respiratory function and cardiac sympathovagal balance in elderly subjects: a randomised controlled trial. BMJ Open 2011 January 1;1(1):e000085. Inappropriate Study Design
Ref ID: 123

(374) Saper RB, Sherman KJ, Cullum-Dugan D, Davis RB, Phillips RS, Culpepper L. Yoga for chronic low back pain in a predominantly minority population: a pilot randomized controlled trial. Altern Ther Health Med 2009 November;15(6):18-27. Inappropriate Study Design
Ref ID: 184

(375) Sareen S, Kumari V, Gajebasia KS, Gajebasia NK. Yoga: a tool for improving the quality of life in chronic pancreatitis. World J Gastroenterol 2007 January 21;13(3):391-7. Inappropriate Study Design
Ref ID: 242

(376) Sarenmalm EK, Martensson LB, Holmberg SB, Andersson BA, Oden A, Bergh I. Mindfulness based stress reduction study design of a longitudinal randomized controlled complementary intervention in women with breast cancer. BMC Complement Altern Med 2013;13. Inappropriate Study Design
Ref ID: 726

(377) Saxena VS, Nadkarni VV. Nonpharmacological treatment of epilepsy. Ann Indian Acad Neurol 2011 July;14(3):148-52. Inappropriate Study Design
Ref ID: 121

(378) Schmitz-Hubsch T, Pyfer D, Kielwein K, Fimmers R, Klockgether T, Wullner U. Qigong exercise for the symptoms of Parkinson's disease: a randomized, controlled pilot study. Mov Disord 2006 April;21(4):543-8. Inappropriate Study Design
Ref ID: 252

(379) Scotte F. The importance of supportive care in optimizing treatment outcomes of patients with advanced prostate cancer. Oncologist 2012;17 Suppl 1:23-30. Inappropriate Study Design
Ref ID: 337

(380) Selfridge N. Meditation for fibromyalgia: Yea or nay? Altern Med Alert 2011 March;14(3):34-6. Inappropriate Study Design
Ref ID: 429

(381) Selfridge NJ. Tai chi for fibromyalgia: Marshalling the art of movement against pain. Altern Med Alert 2010 November;13(11):126-7. Inappropriate Study Design
Ref ID: 430

(382) Selfridge NJ. Rehabilitation for chronic stroke: better balance through yoga? Integr Med Alert 2012 October;15(10):117-8. Inappropriate Study Design
Ref ID: 431

(383) Sharafkhaneh A, Velamuri S, Melendez J, Akhtar F, Hirshkowitz M. Medical management of fatigue. Sleep Med Clin 2013;8(2):265-76. Inappropriate Study Design
Ref ID: 394

(384) Shen CL, Chyu MC, Pence BC, Yeh JK, Zhang Y, Felton CK, Doctolero S, Wang JS. Green tea polyphenols supplementation and tai chi exercise for postmenopausal osteopenic women: safety and quality of life report. BMC Complement Altern Med 2010;10:76. Inappropriate Study Design
Ref ID: 155

(385) Shen CL, Chyu MC, Wang JS. Tea and bone health: steps forward in translational nutrition. Am J Clin Nutr 2013;98(6):1694S-9S. Inappropriate Intervention
Ref ID: 731

(386) Shen YH, Nahas R. Complementary and alternative medicine for treatment of irritable bowel syndrome. Can Fam Physician 2009 February;55(2):143-8. Inappropriate Outcomes
Ref ID: 202

(387) Shengelia R, Parker SJ, Ballin M, George T, Reid MC. Complementary therapies for osteoarthritis: are they effective? Pain Manag Nurs 2013;14(4):E274-E288. Inappropriate Study Design
Ref ID: 732

(388) Sherman KJ. Guidelines for developing yoga interventions for randomized trials. Evid Based Complement Alternat Med 2012. Inappropriate Study Design
Ref ID: 733

(389) Siedentopf F, Utz-Billing I, Gairing S, Schoenegg W, Kentenich H, Kollak I. Yoga for patients with early breast cancer and its impact on quality of life - A randomized controlled trial. Geburtshilfe Frauenheilkd 2013;73(4):311-7. Inappropriate Study Design
Ref ID: 735

(390) Simpson J, Mapel T. An investigation into the health benefits of mindfulness-based stress reduction (MBSR) for people living with a range of chronic physical illnesses in New Zealand. N Z Med J 2011 July 8;124(1338):68-75. Inappropriate Study Design
Ref ID: 127

(391) Singh S, Soni R, Singh KP, Tandon OP. Effect of yoga practices on pulmonary function tests including transfer factor of lung for carbon monoxide (TLCO) in asthma patients. Indian J Physiol Pharmacol 2012 January;56(1):63-8. Inappropriate Study Design
Ref ID: 339

(392) Sirohi B, Sinha N, Goel NS, Badwe RA. Angelina's choice: private decision, public impact. Indian J Med Ethics 2014 January;11(1):34-5. Inappropriate Outcomes
Ref ID: 340

(393) Skoglund L, Josephson M, Wahlstedt K, Lampa E, Norback D. Qigong training and effects on stress, neck-shoulder pain and life quality in a computerised office environment. Complement Ther Clin Pract 2011 February;17(1):54-7. Inappropriate Study Design
Ref ID: 153

(394) Skoro-Kondza L, Tai SS, Gadelrab R, Drincevic D, Greenhalgh T. Community based yoga classes for type 2 diabetes: an exploratory randomised controlled trial. BMC Health Serv Res 2009;9:33. Inappropriate Study Design
Ref ID: 201

(395) Slader CA, Reddel HK, Spencer LM, Belousova EG, Armour CL, Bosnic-Anticevich SZ, Thien FCK, Jenkins CR. Double blind randomised controlled trial of two different breathing techniques in the management of asthma. Thorax 2006;61(8):651-6. Inappropriate Study Design
Ref ID: 737

(396) Smith C, Hancock H, Blake-Mortimer J, Eckert K. A randomised comparative trial of yoga and relaxation to reduce stress and anxiety. Complement Ther Med 2007 June;15(2):77-83. Inappropriate Study Design
Ref ID: 236

(397) Smith HS. Painful rheumatoid arthritis. Pain Physician 2011;14(5):E427-E458. Inappropriate Study Design
Ref ID: 396

(398) Smith JE, Richardson J, Hoffman C, Pilkington K. Mindfulness-Based Stress Reduction as supportive therapy in cancer care: systematic review. J Adv Nurs 2005 November;52(3):315-27. Inappropriate Study Design
Ref ID: 253

(399) Smith KB, Pukall CF. An evidence-based review of yoga as a complementary intervention for patients with cancer. Psychooncology 2009;18(5):465-75. Inappropriate Study Design
Ref ID: 739

(400) Sobstyl M, Tkaczuk-Wlach J, Sobstyl J, Jakiel G. Can non-hormonal therapy be effective in management of menopause symptoms? Przeglad Menopauzalny 2013;12(1):92-6. Inappropriate Outcomes
Ref ID: 740

(401) Sodhi C, Singh S, Bery A. Assessment of the quality of life in patients with bronchial asthma, before and after yoga: a randomised trial. Iran J Allergy Asthma Immunol 2014 February;13(1):55-60. Inappropriate Study Design
Ref ID: 341

(402) Song R, Lee EO, Lam P, Bae SC. Effects of a sun-style tai chi exercise on arthritic symptoms, motivation and the performance of health behaviors in women with osteoarthritis. Taehan Kanho Hakhoe Chi 2007 March;37(2):249-56. Inappropriate Study Design
Ref ID: 238

(403) Song R, Roberts BL, Lee EO, Lam P, Bae SC. A randomized study of the effects of t'ai chi on muscle strength, bone mineral density, and fear of falling in women with osteoarthritis. J Altern Complement Med 2010 March;16(3):227-33. Inappropriate Study Design
Ref ID: 178

(404) Speed-Andrews AE, Stevinson C, Belanger LJ, Mirus JJ, Courneya KS. Pilot evaluation of an Iyengar yoga program for breast cancer survivors. Cancer Nurs 2010 September;33(5):369-81. Inappropriate Study Design
Ref ID: 170

(405) Sprod LK, Mohile SG, Demark-Wahnefried W, Janelsins MC, Peppone LJ, Morrow GR, Lord R, Gross H, Mustian KM. Exercise and cancer treatment symptoms in 408 newly diagnosed older cancer patients. J Geriatr Oncol 2012;3(2):90-7. Inappropriate Study Design
Ref ID: 744

(406) Sprod LK, Janelsins MC, Palesh OG, Carroll JK, Heckler CE, Peppone LJ, Mohile SG, Morrow GR, Mustian KM. Health-related quality of life and biomarkers in breast cancer survivors participating in tai chi chuan. J Cancer Surviv 2012 June;6(2):146-54. Inappropriate Study Design
Ref ID: 109

(407) Stan DL, Collins NM, Olsen MM, Croghan I, Pruthi S. The evolution of mindfulness-based physical interventions in breast cancer survivors. Evid Based Complement Alternat Med 2012. Inappropriate Study Design
Ref ID: 745

(408) Stan DL, Rausch SM, Sundt K, Cheville AL, Youdas JW, Krause DA, Boughey JC, Walsh MF, Cha SS, Pruthi S. Pilates for breast cancer survivors: Impact on physical parameters and quality of life after mastectomy. Clin J Oncol Nurs2012 April;16(2):131-41. Inappropriate Study Design
Ref ID: 71

(409) Stenlund T, Lindstrom B, Granlund M, Burell G. Cardiac rehabilitation for the elderly: Qigong and group discussions. Eur J Cardiovasc Prev Rehabil 2005;12(1):5-11. Inappropriate Study Design
Ref ID: 746

(410) Stenlund T, Birgander LS, Lindahl B, Nilsson L, Ahlgren C. Effects of qigong in patients with burnout: a randomized controlled trial. J Rehabil Med 2009 September;41(9):761-7. Inappropriate Study Design
Ref ID: 188

(411) Stephens S, Feldman BM, Bradley N, Schneiderman J, Wright V, Singh-Grewal D, Lefebvre A, Benseler SM, Cameron B, Laxer R, O'Brien C, Schneider R, Silverman E, Spiegel L, Stinson J, Tyrrell PN, Whitney K, Tse SM. Feasibility and effectiveness of an aerobic exercise program in children with fibromyalgia: results of a randomized controlled pilot trial. Arthritis Rheum 2008 October 15;59(10):1399-406. Inappropriate Study Design
Ref ID: 210

(412) Stoller CC, Greuel JH, Cimini LS, Fowler MS, Koomar JA. Effects of sensory-enhanced yoga on symptoms of combat stress in deployed military personnel. Am J Occup Ther 2012 January;66(1):59-68. Inappropriate Study Design
Ref ID: 102

(413) Strijk JE, Proper KI, van der Beek AJ, van Mechelen W. The Vital@Work Study. The systematic development of a lifestyle intervention to improve older workers' vitality and the design of a randomised controlled trial evaluating this intervention. BMC Public Health 2009;9. Inappropriate Study Design
Ref ID: 750

(414) Suksom D, Siripatt A, Lapo P, Patumraj S. Effects of two modes of exercise on physical fitness and endothelial function in the elderly: exercise with a flexible stick versus tai chi. J Med Assoc Thai 2011 January;94(1):123-32. Inappropriate Study Design
Ref ID: 143

(415) Taibi DM. Sleep disturbances in persons living with HIV. J Assoc Nurses AIDS Care 2013;24(1):S72-S85. Inappropriate Outcomes
Ref ID: 751

(416) Taylor-Piliae RE, Froelicher ES. Effectiveness of tai chi exercise in improving aerobic capacity: a meta-analysis. J Cardiovasc Nurs 2004 January;19(1):48-57. Inappropriate Outcomes
Ref ID: 271

(417) Taylor-Piliae RE, Hoke TM, Hepworth JT, Latt LD, Najafi B, Coull BM. The effect of tai chi on physical function, fall rates and quality of life among older stroke survivors. Arch Phys Med Rehabil 2014 January 16. Inappropriate Study Design
Ref ID: 342

(418) Taylor AH, Cable NT, Faulkner G, Hillsdon M, Narici M, Van der Bij AK. Physical activity and older adults: a review of health benefits and the effectiveness of interventions. J Sports Sci 2004;22(8):703-25. Inappropriate Intervention
Ref ID: 752

(419) Teerlink JR. Mind or body: evaluating mind-body therapy efficacy in heart failure trials. Arch Intern Med 2011 April 25;171(8):758-9. Inappropriate Study Design
Ref ID: 138

(420) Tekur P, Chametcha S, Hongasandra RN, Raghuram N. Effect of yoga on quality of life of CLBP patients: A randomized control study. Int J Yoga 2010 January;3(1):10-7. Inappropriate Study Design
Ref ID: 160

(421) Tiedemann A, O'Rourke S, Sesto R, Sherrington C. A 12-week Iyengar yoga program improved balance and mobility in older community-dwelling people: A pilot randomized controlled trial. J Gerontol A Biol Sci Med Sci 2013;68(9):1068-75. Inappropriate Study Design
Ref ID: 753

(422) Toise SC, Sears SF, Schoenfeld MH, Blitzer ML, Marieb MA, Drury JH, Slade MD, Donohue TJ. Psychosocial and cardiac outcomes of yoga for ICD patients: a randomized clinical control trial. Pacing Clin Electrophysiol 2014 January;37(1):48-62. Inappropriate Study Design
Ref ID: 343

(423) Toise SCF. The efficacy of adapted yoga in managing psychosocial risk in implantable cardioverter defibrillator patients. Ann Arbor: Clark University; 2011.

Inappropriate Study Design
Ref ID: 32

(424) Tong GX, Geng QQ, Cheng J, Chai J, Xia Y, Feng R, Zhang L, Wang DB. Effects of psycho-behavioral interventions on immune functioning in cancer patients: a systematic review. J Cancer Res Clin Oncol 2014;140(1):15-33. Inappropriate Outcomes
Ref ID: 755

(425) Tousignant M, Corriveau H, Kairy D, Berg K, Dubois MF, Gosselin S, Swartz RH, Boulanger JM, Danells C. Tai chi-based exercise program provided via telerehabilitation compared to home visits in a post-stroke population who have returned home without intensive rehabilitation: study protocol for a randomized, non-inferiority clinical trial. Trials 2014;15:42. Inappropriate Study Design
Ref ID: 344

(426) Tsang HW, Mok CK, Au Yeung YT, Chan SY. The effect of qigong on general and psychosocial health of elderly with chronic physical illnesses: a randomized clinical trial. Int J Geriatr Psychiatry 2003 May;18(5):441-9. Inappropriate Study Design
Ref ID: 273

(427) Tsang HWH, Tsang WWN, Jones AYM, Fung KMT, Chan AHL, Chan EP, Au DWH. Psycho-physical and neurophysiological effects of qigong on depressed elders with chronic illness. Aging Ment Health 2013;17(3):336-48. Inappropriate Study Design
Ref ID: 757

(428) Tsang T, Orr R, Lam P, Comino EJ, Singh MF. Health benefits of tai chi for older patients with type 2 diabetes: the "Move It For Diabetes study"--a randomized controlled trial. Clin Interv Aging 2007;2(3):429-39. Inappropriate Study Design
Ref ID: 224

(429) Tsang T, Orr R, Lam P, Comino E, Singh MF. Effects of tai chi on glucose homeostasis and insulin sensitivity in older adults with type 2 diabetes: a randomised double-blind sham-exercise-controlled trial. Age Ageing 2008 January;37(1):64-71. Inappropriate Study Design
Ref ID: 227

(430) Uhlig T. Tai chi and yoga as complementary therapies in rheumatologic conditions. Best Pract Res Clin Rheumatol 2012 June;26(3):387-98. Inappropriate Study Design
Ref ID: 82

(431) Umadevi P, Ramachandra, Varambally S, Philip M, Gangadhar BN. Effect of yoga therapy on anxiety and depressive symptoms and quality-of-life among caregivers of in-patients with neurological disorders at a tertiary care center in India: A randomized controlled trial. Indian J Psychiatry 2013 July;55(Suppl 3):S385-S389. Inappropriate Study Design
Ref ID: 345

(432) Uthman OA, van der Windt DA, Jordan JL, Dziedzic KS, Healey EL, Peat GM, Foster NE. Exercise for lower limb osteoarthritis: systematic review incorporating trial sequential analysis and network meta-analysis. BMJ 2013;347. Inappropriate Intervention
Ref ID: 760

(433) Vadiraja HS, Rao MR, Nagarathna R, Nagendra HR, Rekha M, Vanitha N, Gopinath KS, Srinath BS, Vishweshwara MS, Madhavi YS, Ajaikumar BS, Bilimagga SR, Rao N. Effects of yoga program on quality of life and affect in early breast cancer patients undergoing adjuvant radiotherapy: a randomized controlled trial. Complement Ther Med 2009 October;17(5-6):274-80. Inappropriate Study Design
Ref ID: 185

(434) Vadiraja SH, Rao MR, Nagendra RH, Nagarathna R, Rekha M, Vanitha N, Gopinath SK, Srinath B, Vishweshwara M, Madhavi Y, Ajaikumar S, Ramesh SB, Rao N. Effects of yoga on symptom management in breast cancer patients: A randomized controlled trial. Int J Yoga 2009 July;2(2):73-9. Inappropriate Study Design
Ref ID: 193

(435) van der Heijden MMP, van Dooren FEP, Pop VJM, Pouwer F. Effects of exercise training on quality of life, symptoms of depression, symptoms of anxiety and emotional well-being in type 2 diabetes mellitus: a systematic review. Diabetologia 2013;56(6):1210-25. Inappropriate Study Design
Ref ID: 762

(436) van der Kolk NM, King LA. Effects of exercise on mobility in people with Parkinson's disease. Mov Disord 2013;28(11):1587-96. Inappropriate Study Design
Ref ID: 401

(437) Van Malderen L, Mets T, Gorus E. Interventions to enhance the quality of life of older people in residential long-term care: A systematic review. Ageing Res Rev 2013;12(1):141-50. Inappropriate Study Design
Ref ID: 763

(438) van Uden-Kraan CF, Chinapaw MJM, Drossaert CHC, Verdonck-de Leeuw IM, Buffart LM. Cancer patients' experiences with and perceived outcomes of yoga: results from focus groups. Support Care Cancer 2013;21(7):1861-70. Inappropriate Study Design
Ref ID: 764

(439) Vancampfort D, Probst M, Helvik SL, Catalan-Matamoros D, Lundvik-Gyllensten A, Gomez-Conesa A, Ijntema R, De HM. Systematic review of the benefits of Phys Ther within a multidisciplinary care approach for people with schizophrenia. Phys Ther 2012 January;92(1):11-23. Inappropriate Study Design
Ref ID: 115

(440) Vancampfort D, Vansteelandt K, Scheewe T, Probst M, Knapen J, De HA, De HM. Yoga in schizophrenia: a systematic review of randomised controlled trials. Acta Psychiatr Scand 2012 July;126(1):12-20. Inappropriate Study Design
Ref ID: 97

(441) Vancampfort D, Vanderlinden J, De HM, Soundy A, Adamkova M, Skjaerven LH, Catalan-Matamoros D, Lundvik GA, Gomez-Conesa A, Probst M. A systematic review of Phys Ther interventions for patients with anorexia and bulemia nervosa. Disabil Rehabil 2013 July 4. Inappropriate Population
Ref ID: 346

(442) Varambally S, Vidyendaran S, Sajjanar M, Thirthalli J, Hamza A, Nagendra HR, Gangadhar BN. Yoga-based intervention for caregivers of outpatients with psychosis: a randomized controlled pilot study. Asian J Psychiatr 2013 April;6(2):141-5. Inappropriate Study Design
Ref ID: 347

(443) Velikonja O, Curic K, Ozura A, Jazbec SS. Influence of sports climbing and yoga on spasticity, cognitive function, mood and fatigue in patients with multiple sclerosis. Clin Neurol Neurosurg 2010 September;112(7):597-601. Inappropriate Study Design
Ref ID: 174

(444) Vempati R, Bijlani RL, Deepak KK. The efficacy of a comprehensive lifestyle modification programme based on yoga in the management of bronchial asthma: a randomized controlled trial. BMC Pulm Med 2009;9:37. Inappropriate Study Design
Ref ID: 191

(445) Vera FM, Manzaneque JM, Maldonado EF, Carranque GA, Rodriguez FM, Blanca MJ, Morell M. Subjective Sleep quality and hormonal modulation in long-term yoga practitioners. Biological Psychology 2009;81(3):164-8. Inappropriate Study Design
Ref ID: 768

(446) Visceglia E, Lewis S. Yoga therapy as an adjunctive treatment for schizophrenia: a randomized, controlled pilot study. J Altern Complement Med 2011 July;17(7):601-7. Inappropriate Study Design
Ref ID: 133

(447) Vogler J, O'Hara L, Gregg J, Burnell F. The impact of a short-term iyengar yoga program on the health and well-being of physically inactive older adults. Int J Yoga Therap 2011;(21):61-72. Inappropriate Study Design
Ref ID: 151

(448) von TP, Wiedemann AM, Ludtke R, Reishauer A, Willich SN, Witt CM. Qigong and exercise therapy for elderly patients with chronic neck pain (QIBANE): a randomized controlled study. J Pain 2009 May;10(5):501-8. Inappropriate Study Design
Ref ID: 200

(449) Voukelatos A, Metcalfe A. Central Sydney tai chi trial: Methodology. N S W Public Health Bull 2002 January;13(1-2):19. Inappropriate Study Design
Ref ID: 275

(450) Voukelatos A, Merom D, Rissel C, Sherrington C, Watson W, Waller K. The effect of walking on falls in older people: the 'Easy Steps to Health' randomized controlled trial study protocol. BMC Public Health 2011;11. Inappropriate Study Design
Ref ID: 771

(451) Wang C, Schmid CH, Hibberd PL, Kalish R, Roubenoff R, Rones R, Okparavero A, McAlindon T. Tai chi for treating knee osteoarthritis: designing a long-term follow up randomized controlled trial. BMC Musculoskelet Disord 2008;9:108. Inappropriate Study Design
Ref ID: 213

(452) Wang C, Schmid CH, Hibberd PL, Kalish R, Roubenoff R, Rones R, McAlindon T. Tai chi is effective in treating knee osteoarthritis: a randomized controlled trial. Arthritis Rheum 2009 November 15;61(11):1545-53. Inappropriate Study Design
Ref ID: 187

(453) Wang C, Schmid CH, Rones R, Kalish R, Yinh J, Goldenberg DL, Lee Y, McAlindon T. A randomized trial of tai chi for fibromyalgia. N Engl J Med 2010 August 19;363(8):743-54. Inappropriate Study Design
Ref ID: 163

(454) Wang CC, Collet JP, Lau J. The effect of tai chi on health outcomes in patients with chronic conditions - A systematic review. Arch Intern Med 2004;164(5):493-501. Inappropriate Study Design
Ref ID: 772

(455) Wang CC, Bannuru R, Ramel J, Kupelnick B, Scott T, Schmid CH. Tai chi on psychological well-being: systematic review and meta-analysis. BMC Complement Altern Med 2010;10. Inappropriate Outcomes
Ref ID: 776

(456) Wang CC. Tai chi and rheumatic diseases. Rheum Dis Clin North Am 2011;37(1):19. Inappropriate Outcomes
Ref ID: 777

(457) Wang CC. Role of tai chi in the treatment of rheumatologic diseases. Curr Rheumatol Rep 2012;14(6):598-603. Inappropriate Study Design
Ref ID: 778

(458) Wang W, Sawada M, Noriyama Y, Arita K, Ota T, Sadamatsu M, Kiyotou R, Hirai M, Kishimoto T. Tai chi exercise versus rehabilitation for the elderly with cerebral vascular disorder: a single-blinded randomized controlled trial. Psychogeriatrics 2010 September;10(3):160-6. Inappropriate Study Design
Ref ID: 161

(459) Wang WC, Zhang AL, Rasmussen B, Lin LW, Dunning T, Kang SW, Park BJ, Lo SK. The effect of tai chi on psychosocial well-being: a systematic review of randomized controlled trials. J Acupunct Meridian Stud 2009 September;2(3):171-81. Inappropriate Study Design
Ref ID: 165

(460) Wang XQ, Huang LY, Liu Y, Li JX, Wu X, Li HP, Wang L. Effects of tai chi program on neuromuscular function for patients with knee osteoarthritis: study protocol for a randomized controlled trial. Trials 2013;14. Inappropriate Study Design
Ref ID: 781

(461) Wayne PM, Krebs DE, Wolf SL, Gill-Body KM, Scarborough DM, McGibbon CA, Kaptchuk TJ, Parker SW. Can Tai Chi improve vestibulopathic postural control? Arch Phys Med Rehabil 2004 January;85(1):142-52. Inappropriate Study Design
Ref ID: 272

(462) Wayne PM, Kiel DP, Krebs DE, Davis RB, Savetsky-German J, Connelly M, Buring JE. The effects of tai chi on bone mineral density in postmenopausal women: A systematic review. Arch Phys Med Rehabil2007;88(5):673-80. Inappropriate Outcomes
Ref ID: 783

(463) Wayne PM, Buring JE, Davis RB, Connors EM, Bonato P, Patritti B, Fischer M, Yeh GY, Cohen CJ, Carroll D, Kiel DP. Tai Chi for osteopenic women: design and rationale of a pragmatic randomized controlled trial. BMC Musculoskelet Disord 2010;11:40. Inappropriate Study Design
Ref ID: 177

(464) Wayne PM, Kiel DP, Buring JE, Connors EM, Bonato P, Yeh GY, Cohen CJ, Mancinelli C, Davis RB. Impact of Tai chi exercise on multiple fracture-related risk factors in post-menopausal osteopenic women: a pilot pragmatic, randomized trial. BMC Complement Altern Med 2012;12:7. Inappropriate Study Design
Ref ID: 105

(465) Wayne PM, Manor B, Novak V, Costa MD, Hausdorff JM, Goldberger AL, Ahn AC, Yeh GY, Peng CK, Lough M, Davis RB, Quilty MT, Lipsitz LA. A systems biology approach to studying tai chi, physiological complexity and healthy aging: design and rationale of a pragmatic randomized controlled trial. Contemp Clin Trials 2013 January;34(1):21-34. Inappropriate Study Design
Ref ID: 348

(466) Wenneberg S, Gunnarsson LG, Ahlstrom G. Using a novel exercise programme for patients with muscular dystrophy. Part II: a quantitative study. Disabil Rehabil 2004 May 20;26(10):595-602. Inappropriate Study Design
Ref ID: 262

(467) Wiedemann AM, von TP, Ludtke R, Reisszlihauer A, Willich SN, Witt CM. Developing a qigong intervention and an exercise therapy for elderly patients with chronic neck pain and the study protocol. Forsch Komplementmed 2008 August;15(4):195-202. Inappropriate Study Design
Ref ID: 211

(468) Williams KA, Petronis J, Smith D, Goodrich D, Wu J, Ravi N, Doyle EJ, Jr., Gregory JR, Munoz KM, Gross R, Steinberg L. Effect of Iyengar yoga therapy for chronic low back pain. Pain 2005 May;115(1-2):107-17. Inappropriate Study Design
Ref ID: 255

(469) Windle G, Hughes D, Linck P, Russell I, Woods B. Is exercise effective in promoting mental well-being in older age? A systematic review. Aging Ment Health 2010;14(6):652-69. Inappropriate Intervention
Ref ID: 788

(470) Wolever RQ, Bobinet KJ, McCabe K, Mackenzie ER, Fekete E, Kusnick CA, Baime M. Effective and viable mind-body stress reduction in the workplace: A randomized controlled trial. J Occup Health Psychol 2012;17(2):246-58. Inappropriate Study Design
Ref ID: 790

(471) Wolff M, Sundquist K, Lonn SL, Midlov P. Impact of yoga on blood pressure and quality of life in patients with hypertension - a controlled trial in primary care, matched for systolic blood pressure. BMC Cardiovasc Disord 2013;13. Inappropriate Study Design
Ref ID: 791

(472) Woltz PC, Chapa DW, Friedmann E, Son H, Akintade B, Thomas SA. Effects of interventions on depression in heart failure: A systematic review. Heart Lung 2012;41(5):469-83. Inappropriate Study Design
Ref ID: 792

(473) Woods NF, Mitchell ES, Schnall JG, Cray L, Ismail R, Taylor-Swanson L, Thomas A. Effects of mind-body therapies on symptom clusters during the menopausal transition. Climacteric 2014;17(1):10-22. Inappropriate Outcomes
Ref ID: 793

(474) Woolery A, Myers H, Sternlieb B, Zeltzer L. A yoga intervention for young adults with elevated symptoms of depression. Altern Ther Health Med 2004 March;10(2):60-3. Inappropriate Study Design
Ref ID: 268

(475) Wooton AC. An integrative review of tai chi research an alternative form of physical activity to improve balance and prevent falls in older adults. Orthopaedic Nursing 2010;29(2):108-16. Inappropriate Study Design
Ref ID: 794

(476) Wren AA, Wright MA, Carson JW, Keefe FJ. Yoga for persistent pain: New findings and directions for an ancient practice. Pain 2011;152(3):477-80. Inappropriate Study Design
Ref ID: 795

(477) Wu Y, Wang YT, Burgess EO, Wu J. The effects of tai chi exercise on cognitive function in older adults: A meta-analysis. J Sport Health Sci 2013;2(4):193-203. Inappropriate Outcomes
Ref ID: 796

(478) Xiang YT, Weng YZ, Leung CM, Tang WK, Chan SSM, Wang CY, Han B, Ungvari GS. Gender differences in sociodemographic and clinical characteristic and the quality of life of Chinese schizophrenia patients. Aust N Z J Psychiatry 2010;44(5):450-5. Inappropriate Study Design
Ref ID: 76

(479) Xiao-Dan L, Hong-Zhu J, Hin-Po Ng B, Yi-Huang G, Yun-Chuan W, Gan L. Therapeutic effects of qigong in patients with COPD: A randomized controlled trial. Hong Kong J Occup Ther 2012 June;22(1):38-46. Inappropriate Study Design
Ref ID: 436

(480) Xu YH, Wang JH, Li HF, Zhu XH, Wang G. [Efficacy of integrative respiratory rehabilitation training in exercise ability and quality of life of patients with chronic obstructive pulmonary disease in stable phase: a randomized controlled trial]. Zhong Xi Yi Jie He Xue Bao 2010 May;8(5):432-7. Inappropriate Study Design
Ref ID: 172

(481) Yan JH, Gu WJ, Sun J, Zhang WX, Li BW, Pan L. Efficacy of tai chi on pain, stiffness and function in patients with osteoarthritis: A meta-analysis. PLoS ONE 2013;8(4). Inappropriate Outcomes
Ref ID: 798

(482) Yan JH, Guo YZ, Yao HM, Pan L. Effects of tai chi in patients with chronic obstructive pulmonary disease: preliminary evidence. PLoS One 2013;8(4):e61806. Inappropriate Outcomes
Ref ID: 350

(483) Yang PY, Ho KH, Chen HC, Chien MY. Exercise training improves sleep quality in middle-aged and older adults with sleep problems: a systematic review. J Physiother 2012;58(3):157-63. Inappropriate Intervention
Ref ID: 799

(484) Yardi N. Yoga for control of epilepsy. Seizure 2001 January;10(1):7-12. Inappropriate Study Design
Ref ID: 279

(485) Yeh GY, Wood MJ, Lorell BH, Stevenson LW, Eisenberg DM, Wayne PM, Goldberger AL, Davis RB, Phillips RS. Effects of tai chi mind-body movement therapy on functional status and exercise capacity in patients with chronic heart failure: a randomized controlled trial. Am J Med 2004 October 15;117(8):541-8. Inappropriate Study Design
Ref ID: 260

(486) Yeh GY, Mietus JE, Peng CK, Phillips RS, Davis RB, Wayne PM, Goldberger AL, Thomas RJ. Enhancement of sleep stability with tai chi exercise in chronic heart failure: preliminary findings using an ECG-based spectrogram method. Sleep Med 2008 July;9(5):527-36. Inappropriate Study Design
Ref ID: 231

(487) Yeh GY, Wayne PM, Phillips RS. Tai chi exercise in patients with chronic heart failure. Med Sport Sci 2008;52:195-208. Inappropriate Study Design
Ref ID: 219

(488) Yeh GY, Roberts DH, Wayne PM, Davis RB, Quilty MT, Phillips RS. Tai chi exercise for patients with chronic obstructive pulmonary disease: a pilot study. Respir Care 2010 November;55(11):1475-82. Inappropriate Study Design
Ref ID: 158

(489) Yeh GY, McCarthy EP, Wayne PM, Stevenson LW, Wood MJ, Forman D, Davis RB, Phillips RS. Tai chi exercise in patients with chronic heart failure: a randomized clinical trial. Arch Intern Med 2011 April 25;171(8):750-7. Inappropriate Study Design
Ref ID: 139

(490) Yeh GY, Wood MJ, Wayne PM, Quilty MT, Stevenson LW, Davis RB, Phillips RS, Forman DE. Tai chi in patients with heart failure with preserved ejection fraction. Congest Heart Fail 2013 March;19(2):77-84. Inappropriate Study Design
Ref ID: 351

(491) Yeom HA, Keller C, Fleury J. Interventions for promoting mobility in community-dwelling older adults. J Am Acad Nurse Pract 2009;21(2):95-100. Inappropriate Study Design
Ref ID: 805

(492) Zahavich ANR, Robinson JA, Paskevich D, Culos-Reed SN. Examining a therapeutic yoga program for prostate cancer survivors. Integr Cancer Ther2013;12(2):113-25. Inappropriate Study Design
Ref ID: 806

(493) Zeeuwe PE, Verhagen AP, Bierma-Zeinstra SM, van RE, Faber MJ, Koes BW. The effect of Tai chi chuan in reducing falls among elderly people: design of a randomized clinical trial in the Netherlands [ISRCTN98840266]. BMC Geriatr 2006;6:6. Inappropriate Study Design
Ref ID: 352

(494) Zernicke KA, Campbell TS, Blustein PK, Fung TS, Johnson JA, Bacon SL, Carlson LE. Mindfulness-based stress reduction for the treatment of irritable bowel syndrome symptoms: A randomized wait-list controlled trial. Int J Behav Med 2013;20(3):385-96. Inappropriate Study Design
Ref ID: 807

(495) Zernicke KA, Campbell TS, Speca M, McCabe-Ruff K, Flowers S, Dirkse DA, Carlson LE. The eCALM Trial-eTherapy for cancer appLying mindfulness: online mindfulness-based cancer recovery program for underserved individuals living with cancer in Alberta: protocol development for a randomized wait-list controlled clinical trial. BMC Complement Altern Med 2013;13. Inappropriate Study Design
Ref ID: 808

(496) Zhang F, Kong LL, Zhang YY, Li SC. Evaluation of impact on health-related quality of life and cost effectiveness of traditional Chinese medicine: A systematic review of randomized clinical trials. J Altern Complement Med 2012 December;18(12):1108-20. Inappropriate Study Design
Ref ID: 354

(497) Zhang L, Layne C, Lowder T, Liu J. A review focused on the psychological effectiveness of tai chi on different populations. Evid Based Complement Alternat Med 2012;1-9. Inappropriate Study Design
Ref ID: 811

(498) Zhang YJ, Wang R, Chen PJ, Yu DH. Effects of tai chi chuan training on cellular immunity in post-surgical non-small cell lung cancer survivors: A randomized pilot trial. J Sport Health Sci 2013;2(2):104-8. Inappropriate Study Design
Ref ID: 812

(499) Zhuang SM, An SH, Zhao Y. Yoga effects on mood and quality of life in Chinese women undergoing heroin detoxification: a randomized controlled trial. Nurs Res 2013 July;62(4):260-8. Inappropriate Study Design
Ref ID: 356

(500) Ziv A, Vogel O, Keret D, Pintov S, Bodenstein E, Wolkomir K, Doenyas K, Mirovski Y, Efrati S. Comprehensive Approach to Lower Blood Pressure (CALM-BP): A randomized controlled trial of a multifactorial lifestyle intervention. J Hum Hypertens 2013 October;27(10):594-600. Inappropriate Study Design
Ref ID: 357
