# Supplementary material for: Meditative Movement Therapies and Health-Related Quality-of-Life in Adults: A Systematic Review of Meta-Analyses
Source: PLoS One. 2015 Jun 8;10(6):e0129181. doi: 10.1371/journal.pone.0129181 (PMC4459806; doi:10.1371/journal.pone.0129181)
Supplement: S1 Table — This table includes the results of the AMSTAR assessment for each item from each study. (DOCX) [file pone.0129181.s003.docx]

**Table S1.** Item by item results using the AMSTAR assessment instrument [17–20].

| **Reference** | **‘A priori’ design provided?** | **Duplicate study selection and data extraction?** | **Comprehensive literature search?** | **Status of publication avoided as inclusion criterion?** | **List of included/ excluded studies provided?** | **Characteristics of studies provided?** | **Scientific quality assessed?** | **Scientific quality of studies used appropriately in conclusions?** | **Methods for combining studies appropriate?** | **Publication bias assessed?** | **Conflict of interest stated** | **Total score (%)^a^** |
| --- | --- | --- | --- | --- | --- | --- | --- | --- | --- | --- | --- | --- |
| Buffart et al.[37] | Yes | No | No | No | No | Yes | Yes | Yes | Yes | CA | No | 50 |
| Cramer et al.[38] | Yes | Yes | Yes | No | No | Yes | Yes | Yes | Yes | NA | No | 70 |
| Cramer et al.[39] | Yes | Yes | Yes | No | No | Yes | Yes | Yes | Yes | NA | No | 70 |
| Cramer et al.[40] | Yes | CA | Yes | No | No | Yes | Yes | Yes | Yes | NA | No | 67 |
| Lin et al.[41] | Yes | Yes | No | No | No | Yes | Yes | Yes | Yes | NA | No | 60 |
| Pan et al.[42] | Yes | Yes | Yes | No | No | Yes | Yes | Yes | Yes | NA | No | 70 |
| Shneerson et al.[43] | Yes | Yes | Yes | No | No | Yes | Yes | Yes | Yes | NA | No | 70 |
| Wang et al.[44] | Yes | Yes | Yes | No | No | Yes | Yes | Yes | Yes | NA | No | 70 |
| Zeng et al.[45] | Yes | Yes | Yes | No | No | Yes | Yes | Yes | Yes | CA | No | 70 |
| Zhang et al.[46] | Yes | CA | Yes | No | No | Yes | Yes | Yes | Yes | NA | No | 67 |

Notes: CA, can’t answer; NA, not applicable; Possible responses were “Yes”, “No”, “Can’t Answer”, “Not Applicable”. “Can’t Answer” chosen when item is relevant but not described; “Not Applicable” chosen when item is not relevant (for example, insufficient number of studies to assess publication bias)[17–20]; ^a^, scores adjusted for “can’t answer’ and “not applicable” responses.
